# Age-related changes in the primary auditory cortex of newborn, adults and aging bottlenose dolphins (*Tursiops truncatus*) are located in the upper cortical layers

**DOI:** 10.3389/fnana.2023.1330384

**Published:** 2024-01-05

**Authors:** Jean-Marie Graïc, Livio Corain, Livio Finos, Valentina Vadori, Enrico Grisan, Tommaso Gerussi, Ksenia Orekhova, Cinzia Centelleghe, Bruno Cozzi, Antonella Peruffo

**Affiliations:** ^1^Department of Comparative Biomedicine and Food Science, University of Padova, Legnaro, Italy; ^2^Department of Management and Engineering, University of Padova, Vicenza, Italy; ^3^Department of Statistical Sciences, University of Padova, Padua, Italy; ^4^Department of Computer Science and Informatics, London South Bank University, London, United Kingdom

**Keywords:** auditory cortex, bottlenose dolphin (*Tursiops truncatus*), neuromorphology, aging, development, cytoarchitecture

## Abstract

**Introduction:**

The auditory system of dolphins and whales allows them to dive in dark waters, hunt for prey well below the limit of solar light absorption, and to communicate with their conspecific. These complex behaviors require specific and sufficient functional circuitry in the neocortex, and vicarious learning capacities. Dolphins are also precocious animals that can hold their breath and swim within minutes after birth. However, diving and hunting behaviors are likely not innate and need to be learned. Our hypothesis is that the organization of the auditory cortex of dolphins grows and mature not only in the early phases of life, but also in adults and aging individuals. These changes may be subtle and involve sub-populations of cells specificall linked to some circuits.

**Methods:**

In the primary auditory cortex of 11 bottlenose dolphins belonging to three age groups (calves, adults, and old animals), neuronal cell shapes were analyzed separately and by cortical layer using custom computer vision and multivariate statistical analysis, to determine potential minute morphological differences across these age groups.

**Results:**

The results show definite changes in interneurons, characterized by round and ellipsoid shapes predominantly located in upper cortical layers. Notably, neonates interneurons exhibited a pattern of being closer together and smaller, developing into a more dispersed and diverse set of shapes in adulthood.

**Discussion:**

This trend persisted in older animals, suggesting a continuous development of connections throughout the life of these marine animals. Our findings further support the proposition that thalamic input reach upper layers in cetaceans, at least within a cortical area critical for their survival. Moreover, our results indicate the likelihood of changes in cell populations occurring in adult animals, prompting the need for characterization.

## 1 Introduction

The auditory system in cetaceans is crucial to their survival. In an environment essentially deprived of light below 150 m, cetaceans have evolved to rely heavily on echolocation and audition for hunting, avoiding hazards, and social communication, to an extent that is only matched by bats. The auditory system of cetaceans is poorly understood, although it has been the subject of moderate attention up to this day. Due to the nature of these exceptional mammals, functional studies have been made to grade their hearing capacities (Mooney et al., 2012; Southall et al., 2019; Guan and Brookens, 2023). Pioneering studies used a direct mapping technique to precisely delineate the auditory area in the cortex of the bottlenose dolphin *Tursiops truncatus* (Bullock et al., 1968; Ladygina and Supin, 1970; Sokolov et al., 1972; Bullock and Gurevich, 1979; Popov et al., 1986). However, the bulk of the investigations has been carried out in the form of cytoarchitectural studies on fixed post-mortem material (Morgane and Glezer, 1990; Glezer et al., 1992, 1995, 1998; Hof et al., 1992, 2000). More broadly, the primary auditory cortex (A1) is one of the major sensitive pathways that benefitted from extensive research, notably in animal models such as in guinea pig (Hellweg et al., 1977), rat (Roger and Arnault, 1989; Rutkowski et al., 2003), mouse (Tsukano et al., 2016), cat (Merzenich et al., 1975; Winer, 1984, 1985) and primates (Merzenich and Brugge, 1973), reviewed in Malmierca (2015). Its cytoarchitecture is characterized by granular cells, notably in layers II and IV, interspersed with the presence of pyramidal neurons, especially in layers III and V, a high degree of myelination, a relatively strong expression of immunocytochemical markers, and a particularly cell-dense, broad layer IV (Pandya and Sanides, 1973; Imig et al., 1977; Galaburda and Pandya, 1983; Luethke et al., 1989; Morel and Kaas, 1992; Morel et al., 1993; Hackett et al., 1998, 2001; Hof et al., 1999; Jones, 2003; de la Mothe et al., 2006).

The precise organization of the auditory cortex in the dolphin remains largely unexplained. Some authors now plead for the argument of a functionally complex social brain (Hof et al., 2005; Marino et al., 2007, 2008) despite the fact that it presents structurally primordial features (Glezer et al., 1988) which do not fit well with recent brain scaling models (Herculano-Houzel et al., 2014), with some similarities to the hypertrophied auditory system of echolocating bats (and hedgehogs), such as the presence of extraverted neurons in a dense layer II (Morgane et al., 1985, 1986; Sherwood et al., 2005; Salles et al., 2019). Other authors have a more conservative view of the cetacean brain, which should be considered withing its ecological context (Huggenberger, 2008; Barrett and Würsig, 2014).

In this respect, the potentiality of parallel or convergent evolution reaching complex cognition necessary to social relationships, based on a different paradigm from the terrestrial one, would be of tremendous help to uncover the basis of cognition itself.

One key way to study a sensory system, including its cortical part, is to observe its development and comparative anatomy. Neuroscience of aging has been at the center of recent focus, being linked to degenerating diseases (Alzheimer: Davies et al., 1987; Terry, 2006; Yang et al., 2019 and Parkinson: Collier et al., 2017) but also from a neurobiological perspective (Grady, 2012). Cortical neurons in man undergo a maturation from birth to adulthood, from their migration into respective cortical layers within the first year of life to their final position, an expansion of the repertoire of expressed functional proteins (Moore and Guan, 2001), and subsequent changes from adulthood to later elderliness, which are notably different [for review and discussion of age-related volumetric cortical changes see Gennatas et al. (2017)]. During aging, physiological processes include cortical thinning, focal thinning of the mini-column width, relatively increased volumes of cerebrospinal fluid and a decrease in GABAergic signaling, which leads to excitatory-inhibitory imbalance (Van Veluw et al., 2012; Jayakody et al., 2018; Slade et al., 2020). Numerical and area ratio of neurons and capillary vessels have been reported to decrease in aging human brains (Wang et al., 2004), while myelin integrity and dendritic length and arborization diminish (Dickstein et al., 2007).

In the present study, we analyzed neuronal morphological changes in the primary auditory area in young, adult, and aging bottlenose dolphins, one of the most auditory-oriented extant mammalian species.

## 2 Materials and methods

### 2.1 Animal samples

For the present study we used samples from 11 bottlenose dolphins (*Tursiops truncatus*, Montagu, 1821) kept under human care (*n* = 7) or stranded along the Italian coastline (*n* = 4). The animals were divided into three age classes: four newborns (from 1 to 29 days old); four adults (from 10 to 25 years old); and three very old animals (32, 37 and 40 years old respectively). Details are summarized in Table 1. Bodies of all the animals were delivered to the necropsy room of the Department of Comparative Biomedicine and Food Science of the University of Padova for postmortem diagnostic procedures or the necropsy was performed on field by specialized personnel from the same department. Under these circumstances, no ethical permission is required, as the animals were not part of any experiment. Causes of death did not include neuropathology. Brains from the different animals were fixed by immersion in 4% phosphate-buffered formalin and collected at the Mediterranean Marine Mammals Tissue Bank.^1^ The time interval between death and removal of the brain of the cetaceans cannot be determined precisely for all individuals but varied between two and 12 h.

**Table 1:** Data on the sampled specimen.

ID	Age	Age category	Sex	Length (cm)	Weight (kg)	Origin
ID004	37 years	Old	F	—	—	Delfinarium
ID114	9 days	Calf	M	—	—	Delfinarium
ID124	30 days	Calf	M	—	—	Delfinarium
ID133	>30 years	Old	F	248	159.5	Delfinarium
ID159	40 years	Old	M	328	261	Delfinarium
ID192	Adult	Adult	F	240	178.5	Wild—Rosolina (RO)
ID196	Adult	Adult	M	300	219	Wild—Cervia (RA)
ID203	Adult	Adult	M	284	188	Wild—Rimini (RI)
ID123	6 days	Calf	F	—	—	Delfinarium
ID319	Adult	Adult	M	310	—	Wild—Goro (FE)
ID343	1 day	Calf	F	—	—	Delfinarium

### 2.2 Sampling and histology

Identification of A1 in the bottlenose dolphin was based on available references (Kesarev et al., 1977; Supin et al., 1978; Cozzi et al., 2017), mostly based on electrophysiological data. Despite the changes that occurred in the cetacean brain during evolution including intense gyrification, it is notable that primary projection fields of the neocortex are nonetheless arranged somewhat in the same sequence as in many other mammals. The auditory cortex is located in the parieto-temporal lobe, and it seems to broadly correspond with the situation in artiodactyls (Cozzi et al., 2017).

Blocks of nervous tissue were first left in buffered formalin, then washed in phosphate-buffered saline (PBS) 0.1 M, pH 7.4 to be processed for paraffin embedding. Embedded tissue samples were cut into 6 μm-thick sections on which standard Nissl staining was performed. Briefly, sections were deparaffinized in xylene, followed by a scale of hydration in absolute alcohol, 95% alcohol solution, 70% alcohol, and 50% alcohol. After passing in distilled water, sections were put in thionine 0.1% pH 4 at 60°C for 10 min. Then, after rinsing the sections in distilled water, an ascending scale of dehydration was followed by immersion in xylene. The sections were then coverslipped.

### 2.3 Data acquisition and image analysis

Quantitative cytoarchitectonic features were examined in the bottlenose dolphin brain sections using a semi-automated procedure. Briefly, for each animal two digitalized Nissl-stained coronal sections of the auditory cortex were segmented into layers by trained operators (J-MG, AP, TG, KO, and BC) to be processed independently. In each layer, a custom image analysis pipeline based in Matlab (MathWorks, Inc., United States) recognized each neuron (objects as small as glia were ignored) and recorded its morphometric data (Grisan et al., 2018; Vadori et al., 2023).

To identify and delineate neuronal cells we used the method called MR-NOM, which was specifically tested on Nissl-stained histological slices. MR-NOM exploits a multi-scale approach to deliberately over-segment the cells into superpixels and subsequently merge them via a classifier based on shape, structure, and intensity features. It involves six key steps: pre-processing, foreground extraction, marker definition, watershed transform, supervised superpixels merging, post-processing. Briefly, the image processing begins by converting each image to greyscale, applying a 2D Gaussian smoothing filter, and enhancing the image’s contrast. The average neuropil intensity is standardized using a correction factor. The foreground is extracted by employing a multi-scale approach based on Laplacian of Gaussian (LoG) scale-space representations. This involves convolving the pre-processed input image with a set of LoG filters and summing the results to generate a combined multi-scale map, where the regions of interest correspond to high responses. This multi-scale map is added to the input image and thresholded using the triangle method to extract the foreground, which is further refined using mathematical morphology operations. The markers for a watershed-based over-segmentation are obtained by computing a second combined multi-scale map where local maxima are selected via the extended h-maxima transform (we refer to the paper for details). The markers are used to over-segment the cells into superpixels via the marker-controlled watershed transform applied to a gray-scale map that integrates a combined multi-scale map with gradient information. A random forest classifier trained on a manually annotated dataset containing rotation-invariant morphological, structural and intensity features decides whether a pair of adjacent superpixels should be merged because they are part of the same cell. Hole-filling, morphological opening and reconstructions are applied next, followed by the removal of objects smaller than a threshold. Finally, the Chan-Vese model for active contours is exploited to refine the cell shapes and a second random forest classifier filters out false positive findings.

**Figure 1:**
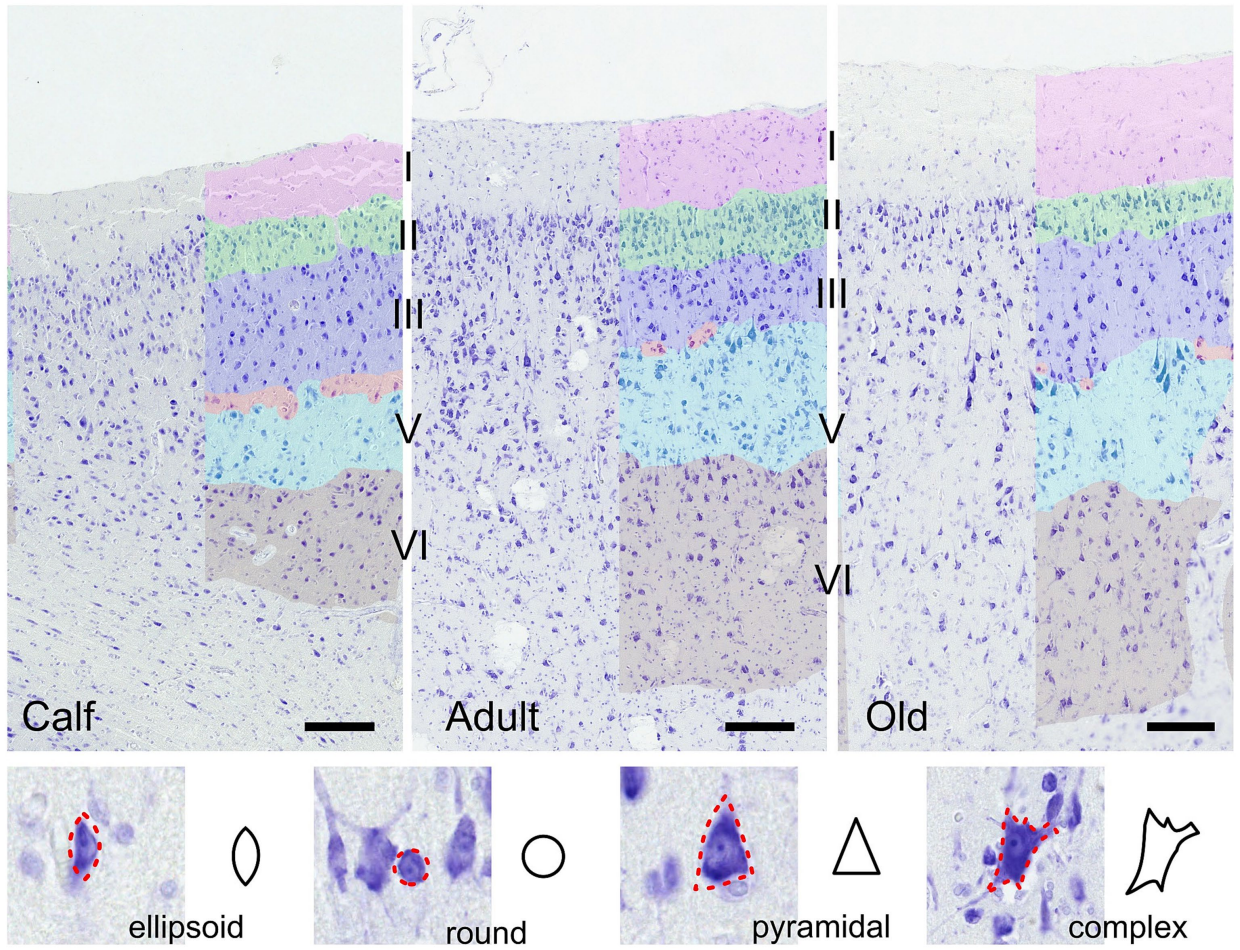
Illustration of the auditory cortex in 3 age groups (calves, adults and old). Layers used in the analysis are colored manually. Bar = 200 μm. Note the presence of cells in the molecular layer in all ages, and the layer IV (red) which is discontinuous, composed of smaller granular or pyramidal cells in a relatively cell-poor line between medium-sized (layer III, dark blue) and large (layer V, light blue) pyramidal cells. Layer II is the most cell-rich, as in all animals. In the section below are shown the four cell types recognized algorithmically and examples of cortical neurons of each type.

Morphometric descriptors were extracted for each delineated neuronal cell. Using these morphometric measurements, each cell was assigned a specific shape—round, ellipsoid, pyramidal, or complex—according to a predefined set of rules (Figure 1). Morphometric descriptors of neural cell morphology were binned in 3 domains: size, shape, and density. The size domain included area, major and minor axis lengths, and perimeter. The shape domain included the inverse aspect ratio (invAR), extent, eccentricity, solidity, and convex circularity. The density domain was assessed by the number of neurons detected within either a 50 or 100 μm radius of each neuron [for reference, see Corain et al. (2020) and Graïc et al. (2023)].

### 2.4 Statistical analysis

The resulting thousands of entries were then funneled to a multivariate statistical analysis pipeline in R (R Core Team, 2021) adapted to large sampling analysis. The analysis compared calf, adult and old populations for each domain for the 6 layers and 4 shape types. The multiple cells recorded in each image are not independent, therefore, resampling to account for repeated measures was used based on the method proposed by Finos and Basso (2014). The method is the following: in each image the mean of each feature is computed and used as a pseudo-observation. The pseudo-observations are hence independent, but not homoscedastic. They are randomly permuted among groups, while an adequate test statistics account for the heteroscedasticity. The *p*-value is the proportion of the test statistics computed on randomly permuted pseudo-observations that exceeds the one computed on observed data. This approach takes into account the joint distribution of the tests and allows for the multivariate inference via nonparametric Fisher combination of the univariate test. The tests are combined by morphometric descriptors, layer and cell type. Significance level was set to alpha = 0.05. The analysis was performed with R software (R Core Team, 2021) and the flip package (Finos, 2018).

To account for the multiplicity of the tested hypotheses, we opted for a correction of the family-wise error rate within each analysis (i.e., domain-wise, layers-wise and type-wise). For example, when comparing the three populations in the six layers, we perform six combined tests, and the multiplicity considered is six. Specifically, we apply a resampling-based approach (Westfall and Young, 1993) that allows us to perform max-t correction, which accounts for dependence among tests and results in less conservative estimates than Bonferroni–Holm procedure when multiple comparisons are involved. This method ensures that the probability of making at least one type I error is controlled at a specified level, such as 0.05, despite conducting multiple tests.

## 3 Results

### 3.1 The auditory cortex layering is characteristically different from that in terrestrial mammals

As already reported by Furutani (2008), thickness varies across cortical areas, and among those, the auditory cortex is the thickest one. The layering of the cortex was nonetheless characteristically different from that of terrestrial mammals. Notably, a thin band of layer IV was hardly seen, without continuity, and characterized by small granule-like cells dispersed between layers III and V, thereby qualifying as quasi-agranular, although primary sensory, cortex (Figure 1). Overall, the column comprised a medium to thick layer I, with few cells present throughout, followed by a very dense layer II including small pyramidal neurons, a layer III characterized by small to medium pyramidal somata which were even larger in layer V. Few small granular cells which could constitute a basis for a layer IV were present discontinuously in a cell-poor line between layers III and V. Most pyramidal neurons were rather round and large compared to typical pyramidal neurons (Figure 1). Layer II was the most cell-rich, as in all mammals. All images considered together, no evident difference could be seen between age classes.

### 3.2 Statistical analysis

#### 3.2.1 Size, shape and density domains combined by layer for all age classes

Cumulative *p*-values for all cell forms showed that most layers contained substantial differences across age classes.

For the domain size, significant differences were found in the layers I, II, V, and VI among calf, adult and old populations. Neurons in the auditory cortex of the layers I, II, V, and VI showed significant differences (*p* = 0.0024, *p* = 0.0124, *p* = 0.0462, and *p* = 0.0324 respectively), in size, among the calf, adult and old populations.

For the domain shape, significant differences were found in the layers I, II, III, V, and VI among calf, adult and old populations. Neurons in the auditory cortex of the layers I, II, III V, and VI showed significant differences (*p* = 0.0004, *p* = 0.0186, *p* = 0.0186, *p* = 0.005, and *p* = 0.0172 respectively), in shape among the calf, adult and old populations.

For the domain density, significant differences were found in the layers II, III, V, and VI among calf, adult and old populations. Neurons in the auditory cortex of the layers II, III V, and VI showed significant differences (*p* = 0.0176, *p* = 0.0186, *p* = 0.0014, *p* = 0.0034, and *p* = 0.0058 respectively), in density among the calf, adult and old populations.

Hence, layer I showed statistical significance in size (*p* ≤ 0.01) and shape (*p* ≤ 0.01), while layer II was significantly different in calves, adults or old in all three size, shape, and density domains (*p* ≤ 0.05 in all cases). Layer III was also significantly different across ages in shape (*p* ≤ 0.05) and density (*p* ≤ 0.01). Layer IV did not yield any specific statistical significance. Layer V also contained significant differences in all three domains size, shape, and density (*p* ≤ 0.05, *p* ≤ 0.01, and *p* ≤ 0.01 respectively), as well as layer VI (*p* ≤ 0.05, *p* ≤ 0.05, and *p* ≤ 0.01 respectively).

The detailed results of the statistical analysis are displayed in the Supplementary material.

#### 3.2.2 Results combined by cell type and layer

In the size domain, statistically significant differences were found for ellipsoid cells in layer I (*p* = 0.006), and in layers I, II, and VI for round cells (*p* = 0.0004, *p* = 0.017, and *p* = 0.0434 respectively).

In the shape domain, statistically significant differences were found for round cells in layers I, II, III, V, and VI (*p* = 0.0002, *p* = 0.024, *p* = 0.0294, *p* = 0.012, and *p* = 0.0158 respectively).

In the density domain, statistically significant differences were found in layers II, III, V, and VI for all cell types (ellipsoid cells: *p* = 0.0284, *p* = 0.0044, *p* = 0.0082, and *p* = 0.0086 respectively; round cells: *p* = 0.0318, *p* = 0.0020, *p* = 0.0066, and *p* = 0.0110 respectively; pyramidal cells: *p* = 0.0254, *p* = 0.0046, *p* = 0.0048, and *p* = 0.0082 respectively; complex cells: *p* = 0.0274, *p* = 0.0048, *p* = 0.0086, and *p* = 0.0102 respectively).

Hence, comparisons by cell type and layer resulted in several notable differences. While ellipsoid cells were significantly different across age classes in layer I (*p* ≤ 0.001) for size only, round cells were markedly different for size and shape in layer I (*p* ≤ 0.001), layers II and VI (*p* ≤ 0.05). Regarding density, all 4 types of cells yielded statistical differences across most layers except layers I and IV, the most salient of which in layers III and V (*p* ≤ 0.01 for all cell types).

#### 3.2.3 Results for each cell type separately by layer

**Figure 2:**
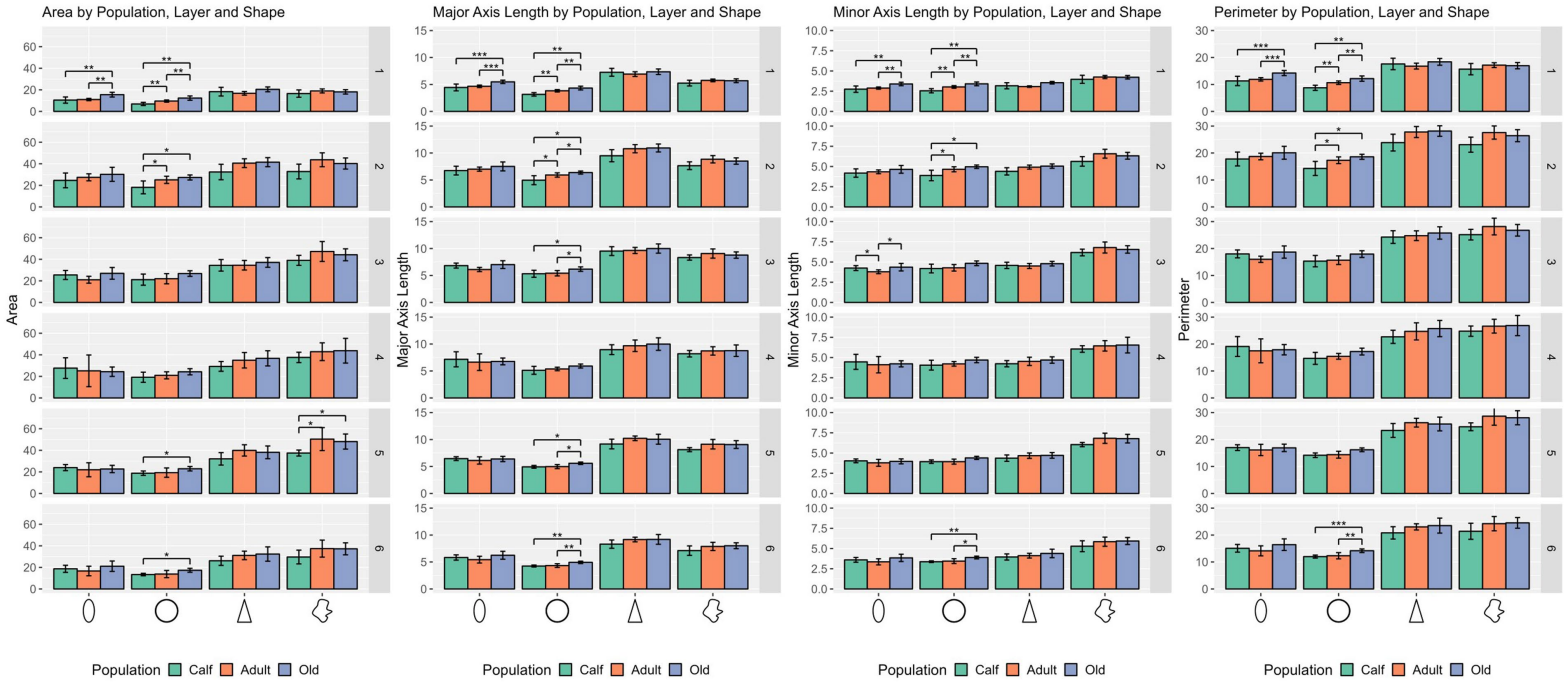
Statistical multivariate analysis results for the domain size, including cell area, cell major axis length, minor axis length and cell perimeter, separated by cell type (from left to right, ellipsoid, round, pyramidal and complex), and the three age classes. Bars are standard deviation. Asterisks represent statistical significance (^*^p ≤ 0.05, ^**^p ≤ 0.01, and ^***^p ≤ 0.001).

**Figure 3:**
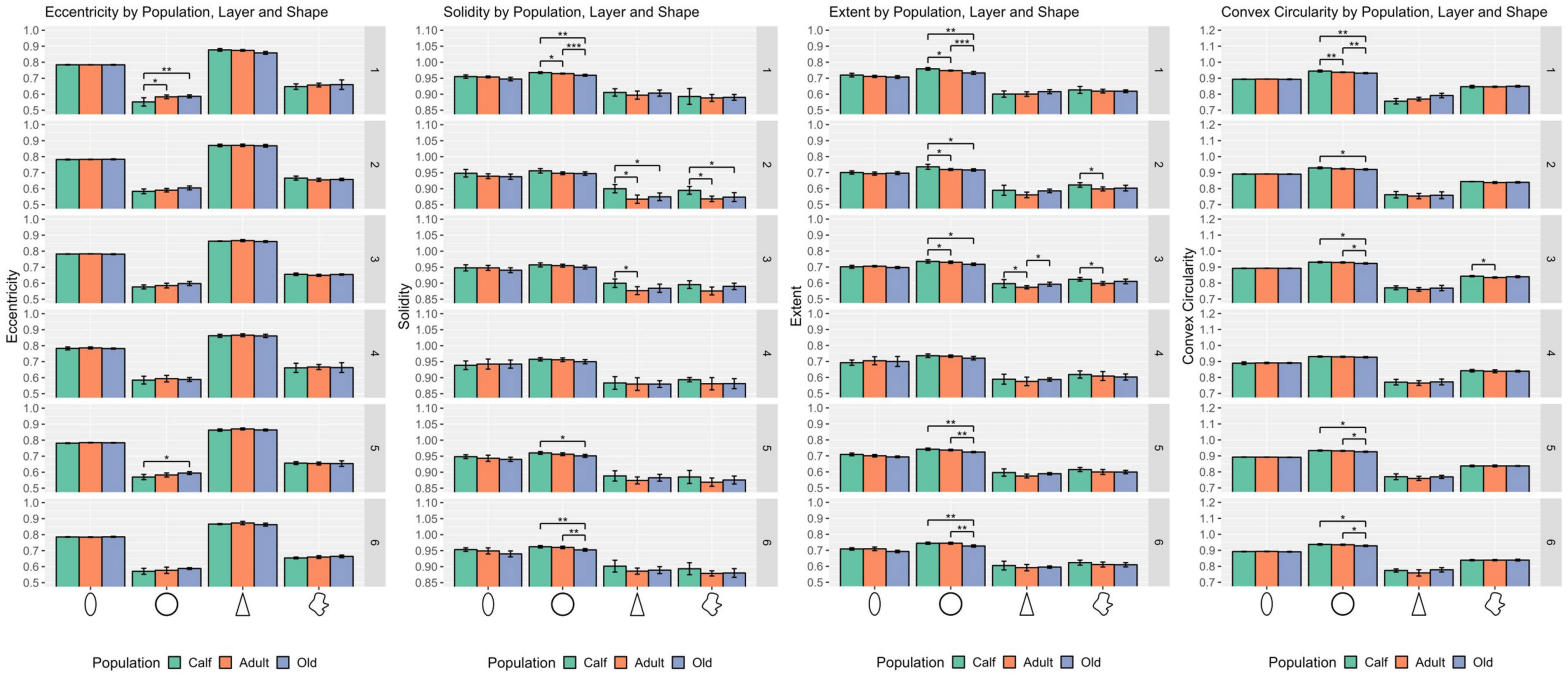
Statistical multivariate analysis results for the domain shape, including cell eccentricity, solidity, extent, and convex circularity, separated by cell type (from left to right, ellipsoid, round, pyramidal and complex), and the three age classes. All values are within a [0–1] range. Bars are standard deviation. Asterisks represent statistical significance (^*^p ≤ 0.05, ^**^p ≤ 0.01, and ^***^p ≤ 0.001).

Round cells were all very well segregated statistically (*p* ≤ 0.001) in layer I, with smaller cells in calves, larger cells in adults, and the largest ones in older subjects (*p* ≤ 0.01 in all cases). Older animals also had significantly larger round cells in layers V and VI. Interestingly, round cells in layer I of older and younger animals were significantly isolated also in shape descriptors (solidity, extent, convex circularity), as well as in layer VI for old specimens. Overall, in most layers, old dolphins’ round cells were less round than adults and calves. This is mirrored in the inverse aspect ratio and eccentricity for upper cortical layers, and extent and solidity for lower layers (Figures 2, 3).

Ellipsoid cells were larger in old animals in layers I and III with strong statistical significance (*p* ≤ 0.001 in major axis length) compared to young and adult ones. In layer III, adults also displayed larger cells than calves for all size descriptors (*p* ≤ 0.05). Old animals had a notably lesser morphological extent in layer VI and solidity in layer I (Figures 2, 3).

Pyramidal cells were significantly shorter in layer V in calves (*p* = 0.049). Layers II, III, and VI showed that calves pyramidal cells had a more restricted shape, centered close to the barycenter of the cell (solidity), while adults and old animals had cells that had more extended shapes (Figures 2, 3).

Complex cells, usually larger than other cells, were notably smaller in layer V in calves, and had a more constricted shape in layers II and III. The most notable difference was in layer V, where calves had the smallest cell area (*p* ≤ 0.05), while all shape parameters remained constant across ages (Figures 2, 3).

**Figure 4:**
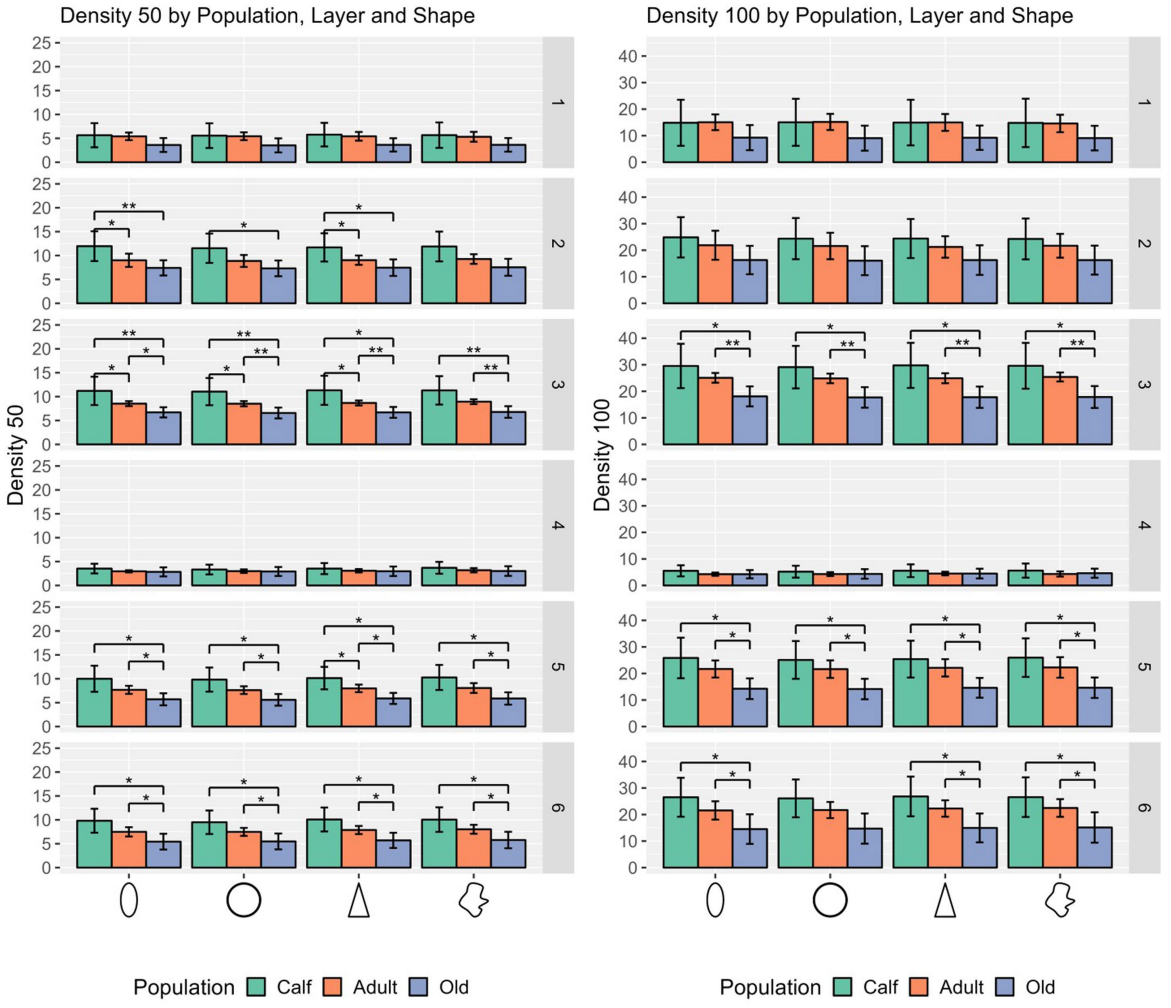
Statistical multivariate analysis results for the domain density, including cell count in a 50 μm radius and a 100 μm radius, separated by cell type (from left to right, ellipsoid, round, pyramidal and complex), and the three age classes. Bars are standard deviation. Asterisks represent statistical significance (^*^p ≤ 0.05, ^**^p ≤ 0.01, and ^***^p ≤ 0.001).

In most cases, calves had denser cortical columns, while adults had an intermediate position, and older animals a sparser distribution of cells, both in a 50 μm and a 100 μm radius. The notable exceptions regard layer I, where most of cell types in calves were not significantly densely packed from adults or old animals, as in layer II in some cases and particularly in layer IV (Figure 4).

## 4 Discussion

This study is the first to apply quantitative multivariate analysis to potential changes occurring in the brain of cetaceans across their lifespan. Changes, observed in the upper layers (I, II, III) and to a lesser extent in lower layers (V and VI), primarily affecting interneurons characterized by round and ellipsoid cell shapes, without significant involvement of pyramidal cells. These modifications may substantially impact the circuitry commonly associated with dolphin brains (Morgane et al., 1985; Hof et al., 2000); reviewed in Cozzi et al. (2017).

### 4.1 The topographical anatomy of the primary auditory cortex (A1) is different from terrestrial mammals

The auditory cortex of the bottlenose dolphin has been the focus of considerable attention from research groups in the past (Hof et al., 1992, 2005; Fung et al., 2005; Furutani, 2008; Kern et al., 2011; Knopf et al., 2016; van Kann et al., 2017); for review see Cozzi et al. (2017) which resulted in a relatively deeper knowledge of its anatomy and cytoarchitecture compared to other areas. Despite these efforts, the overall homogeneity of the cetacean cortex coupled with the limited electrophysiological investigations has not yet permitted to delineate secondary auditory areas accurately, and a large portion of the cortical surface remains unassigned, although it is hypothesized that these areas may be association cortices (Morgane et al., 1980). Recent works using newer methodology have pointed to interesting cortical locations towards the temporal lobe for different functions (Berns et al., 2015; Wright et al., 2018; Gerussi et al., 2023). There are mammalian features particularly reminiscent of other cetartiodactyls, placing the auditory cortex on the parieto-temporal border (Rose, 1942; Michaloudi et al., 1986), although nomenclature in cetaceans is notably different from that of terrestrial mammals due to the modifications of the topography that occurred during their evolution.

Since the precise limits of A1 and the putative A2 are not known, tonotopical maps and associative areas commonly known in cats, rodents and primates remain to be histologically and topographically characterized in cetaceans.

### 4.2 The general A1 cytoarchitecture is peculiar in dolphins

The structure of the auditory cortex in cetaceans is somewhat different from that of other mammals for a number of reasons. First, this primary sensory cortex does not show the expected extensive granulation found in most terrestrial mammals relying on a developed auditory sensitivity (Winer, 1984, 1985). As mentioned earlier, this is true of all sensory cortices in cetaceans (Morgane et al., 1986) where, to summarize, thalamic inputs are believed to access the gray matter through layer I, and descending to layers II then III, ultimately establishing primary outputs in layer V/VI (Cozzi et al., 2017). This implies that the main thalamo-cortical target cells are neurons in layer II, the outer granular layer. Secondly, the cortical thickness in A1, although reported as the thickest within the cetacean brain, is remarkably thin, amounting to about 1.85 mm in the bottlenose dolphin (Furutani, 2008). The cortex of cetaceans has been reported to be homogeneous throughout, with a reduced lamination, exhibiting features akin to that of primordial mammals including extraverted pyramidal neurons (Morgane et al., 1985, 1988; Hof et al., 1992), a notion partially rebuked today (Hof et al., 1999, 2000, 2005; Marino et al., 2007). With this in mind, the auditory cortex in our specimen respected the established structure for the bottlenose dolphin (Cozzi et al., 2017; van Kann et al., 2017; Graïc et al., 2021).

Across all ages, the external molecular layer was relatively large and mostly devoid of cells except for a few (inhibitory) stellate cells. The external granular layer (II) showed a clean dense band of rather medium to small neurons with multipolar somata. The border between layers II and III was uneven but evident with small, dense pyramidal cells forming a narrow band, while neurons in the relatively thin external pyramidal layer (III) were larger and exhibited a more pyramidal appearance. The neurons present in layer II are known to comprise both pyramidal and non-pyramidal cells (Furutani, 2008) and to exhibit the so-called extraverted neurons, a typical primordial feature (Morgane et al., 1988). Below, despite the absence of a clear layer IV, groups of small granular cells were spotted, distinguishable against the upper border of the large layer V, in which pyramidal neurons seemed the largest. Cell surface density seemed appreciably lower in this layer, as well as in layer VI, which could be due to the ascending myelinated fiber bundles reaching for higher cortical target layers, or alternatively to efferent axons leaving A1. Here we also emphasize the potential role of layers V and VI in the descending projections to the inferior colliculus, a pathway yet to be mapped and clarified in dolphins (Vaithiyalingam Chandra Sekaran et al., 2021). Cells of layer VI were arranged rather sparsely with multiform, middle-sized neurons. Although there is commonly no consistent layer IV in cetaceans, it was detected in V1 by Garey and Leuba (1986) in brains of an 18 days-old and 3 years-old dolphins, and more subtly by Morgane et al. (1988) in adults. Considering its inconsistency and the rare cells forming it, the statistical analyses from layer IV did not yield significant results. The absence of a layer IV in sensory cortical areas in cetaceans is not new (Garey et al., 1985; Glezer et al., 1992; Furutani, 2008) although it was reported in the visual cortex (Morgane et al., 1988; Graïc et al., 2021). This implies that such a core cortical area receives inputs through a different circuitry than that considered for most mammals except bats and hedgehogs (Glezer et al., 1992; Cozzi et al., 2017), namely through layer I and II where most of the calcium-binding GABAergic interneurons are found in cetaceans (Glezer et al., 1992; Hof et al., 2000; Graïc et al., 2022). This configuration is indirectly similar to that of the paleocortex, and hence of primordial mammals, common ancestors of both cetaceans and terrestrial mammals over 50 million years ago (Cabrera et al., 2021). The appearance of a proper granular layer in sensory cortices, especially primates (Haug, 1987) in terrestrial mammals likely never happened in the parallel course of cetacean evolution. This, however, poses significant questions about cortical performance and trade-offs to reach the observed auditory features of dolphins.

We observed a less diverse morphology compared to the primary visual cortex (V1) (Graïc et al., 2021), which is not new (Smith and Populin, 2001) and can have a variety of explanations. One is that the auditory cortex reflects the manifestation of binaurality (and the formation of an auditory space map) at a lower level, such as that of the very large caudal (inferior) colliculi in the brainstem, compared to the formation of binocularity, which is formed at the cortical level, and might warrant for a more complex cortical column (Smith and Populin, 2001). An alternative explanation, considering the current top-down input theory in which thalamo-cortical inputs reach layer II from layer I, would be that this rather primordial feature could force the surface extension of the cortex and hence reduce the concentration of sensitive patterns, evening them out over a larger portion of cortex. Additionally, it should be noted that the canonical cortical circuit (column) is based on the striate cortex, which is an extreme, exceptional example of the cortex compared to most mammals and other cortical areas (Constantinople and Bruno, 2013; Godlove et al., 2014; Beul and Hilgetag, 2015). The auditory cortex of delphinids, like several non koniocortical areas in mammals, should be regarded accordingly.

### 4.3 Maturation of A1 from newborn to adult in the bottlenose dolphin

The main differences between newborns and adults were mostly presented by round and ellipsoid cells, although pyramidal cells were also notably more closely centered and circumscribed in newborns.

The molecular layer of newborns, with much fewer cells than other layers, displayed round and ellipsoid cells according to our algorithm, pertaining mostly to the few interneurons and stellate cells commonly described in mammals. However, no subpalial granule layer was observed, which is coherent with the fact that no specimen was of fetal origin (Judaš and Pletikos, 2010). These layer I cells have been correlated to associative learning and attention (Gilbert and Sigman, 2007), receiving input from M-type thalamic cells (Rubio-Garrido et al., 2009). The development across our age classes may be consistent with increased connections in number and distance over time, needing a larger metabolism to sustain them. The cells measured were consistently smaller, more round and denser in newborns with respect to adults, in particular in the case of round cells. It is important to note that pyramidal cells are not found in the molecular layer. Our algorithm, however, detected shapes, which, in some cases, were indeed pyramidal in layer I.

Salient differences were also found in layer II (outer granular), III (outer pyramidal) and V (inner pyramidal) in round, ellipsoid and pyramidal cells. Layer II was determinant in that cells in calves were consistently rounder, smaller, and more dense. These tended to be overall larger but also more slender in adults (see major axis length, extent values), particularly in layer V. Pyramidal neurons followed the same trend, with larger cells in layers III, and V (area and major axis length, *p* ≤ 0.05). Complex cells, representing usually larger cells, were notably larger in newborns with respect to adults and old animals.

Hence, the main differences in size and shape between newborn and adult specimens were formed by round cells in upper layers. Adults had lower invAR values in pyramidal cells although without statistical significance, which would correspond to more slender, elongated cells. Calf round cells were also smaller in all layers. In layers V and VI, round cells in the adult showed the widest standard deviation, while in layers II and III, round cells varied more widely in newborns. Standard deviation in pyramidal cells also showed a trend towards larger and more diverse cells in older animals. These elements point towards a diversification of the different cell types with age.

Regarding the change of surface density, the high values in calves compared to adults correspond to trends in neuronal densities measured in previous studies (Garey and Leuba, 1986), although no steep decline could be seen with increasing age. In the same study, V1 neuronal density was almost twice as high in 18 days old calf compared to adults (ranging from 3–33 years old). A clear developmental drop in density in dolphins is still unclear for any cortical area, when the peak of developmental apoptosis should take place (e.g., mainly within the first 6 months in humans, Huttenlocher, 1979). This study could be seen as a first approach in measuring this for the bottlenose dolphin auditory cortex, and understanding how the decrease in neuronal density may go hand in hand with the simultaneous increase in neuronal complexity. With the exception of layer I, all layers exhibit significantly higher neuronal density in the calves compared to adults. However, it should not be overlooked that the study of Haug et al. (1984) demonstrated that tissue processing shrunk young specimens tissue significantly more, resulting in greater densities compared to older specimens.

### 4.4 Changes in A1 of the bottlenose dolphin in aging animals compared to adults

It is now known that in aging, neuronal loss does not occur in a widespread, general manner as once thought (Morrison and Hof, 1997; Burke and Barnes, 2006; Pannese, 2011; Reuter-Lorenz and Park, 2014). Systematic studies on larger numbers of human brains have concluded that age-related neuronal loss is insignificant (unlike the case in Alzheimer’s disease, where neuronal loss is evident), and that previously reported declines may have arisen from the different tendencies for processing-induced brain shrinkage in tissues from young versus older subjects (Haug et al., 1984). Peters et al. (1998) point out that the environmental parameters have a potential influence on neuronal counts in the brains of same-aged individuals of different generations. Of course, the inability to measure the same individual’s neuronal counts at different ages remains a great limitation. According to this series of confounding factors, we did not attempt to measure any cell loss or gain.

Round and ellipsoid cells of layer I were larger, with a longer perimeter, and a more slender shape in old animals. Stellate and bitufted cells in the molecular layer have been reported to receive specific input from the thalamus, deemed critical to associative learning (Rubio-Garrido et al., 2009) and complex vocalization (Montes-Lourido et al., 2021). The fact that associative learning and memory functions have been, in turn, named as the most sensitive to senescence (Burke and Barnes, 2006), implies that changes observed in these cells deserve further attention. Layer I cells are also essential in intra-cortical connections (D’Souza and Burkhalter, 2017), which in cetaceans could be central to the large primary auditory area.

Aging animals exhibited larger cells with a much larger extent, notably in round and ellipsoid cells, compared to adults in layers V and VI. Again, differences in these types of cell shapes could be related to differences in interneuronal morphology linked to alterations to their dendritic tree and synapses.

The apparent diminishing surface density from adults to older animals is consistent with age-related cortical thinning in primates (Fjell et al., 2009), although there are crucial differences across grey matter nuclei, and layer- and cell type-specific patterns of neuronal loss (Haug et al., 1984).

There seems to be a recent reconsideration of senescence at the cellular level in the brain (Tan et al., 2014), noting that neurons, a non-dividing cell, exhibited hallmark metabolic signs of senescence (Moreno-Blas et al., 2019). These alterations are likely to have minute morphological consequences which could potentially be measured using immunocytochemistry or single-cell morphometrics. Our results point towards larger and more slender cells in old animals, which could be coherent with the accumulation of material such as lipofuscin, or tau protein. The overall effect of these changes together with well-accepted alterations in dendritic trees (Pannese, 2011; Mota et al., 2019) is hard to quantify. However, current aging models do take them into account (Park and Reuter-Lorenz, 2009; Reuter-Lorenz and Park, 2014). Under such a model, aging brains cope in a variety of ways to perform under more stringent conditions. Interestingly, higher cognitive function circuits such as the “default mode” network seem to be affected much more than basic function and primary information processing (Geerligs et al., 2015), such as the present primary auditory cortex. However, there is some evidence for the potential reduction of acoustic/echolocating acuity of older dolphins (Mann et al., 2010), although no consideration can yet be made on potential cortical thinning and other structural changes associated with hearing loss, as reported in humans (Ha et al., 2020; Roseman and Thiel, 2020).

Resolution and consequent software limits suggested us to exclude glial cells from the present study. However, glial cell gene expression in humans was found to correlate with grey matter volume changes in both development and aging (Vidal-Pineiro et al., 2020), thus neuronal-glial interrelationship may play a role in animals with a relatively high degree of myelination (Ridgway et al., 2019).

### 4.5 Tissue fixation and limitations

For decades the preservation of tissue has been a point of importance. The fixation by immersion technique, even though widely used for rodents, has been argued to be unsuitable for large specimens, given the speed of formalin diffusion. Perfusion fixation is therefore preferable, where formalin or a substitute is injected into a main vessel to remove the intravascular blood form the whole brain before its extraction and immersion. Despite our efforts, this is seldom feasible from a practical point of view, since animals must undergo diagnostic necropsy and therefore cannot be perfused beforehand, and major vessels are cut afterwards. More importantly, in cetaceans, the reduced importance of the internal carotid and—on the contrary—the relevant role of the *retia mirabilia* in blood supply to the brain adds to the challenge (Nagel et al., 1968; Cozzi et al., 2017; Bonato et al., 2019). Hence, if whole-body perfusion is not possible, brain only perfusion proves arduous. Additionally, the time after stranding, *a fortiori* the post-mortem interval, affect vascular permeability and blood clotting, and therefore perfusion is not guaranteed to reach the capillary level despite repeated washing. The effects of fixation have also been discussed (Garman, 1990; McInnes, 2005, 2012; Garman, 2011).

Tissue autolysis typically entails pyknotic cells with densely, retracted somata, and the vacuolization of the external cellular matrix, which can be coherent with increased distance between cells, and increased solidity and extent. These features appear with various intensities depending on circumstances of death and postmortem interval conditions (Ujihira et al., 1993; Sheleg et al., 2008).

An important artifact to consider is that of tissue shrinkage (Quester and Schröder, 1997), which may result from combination of poor fixation and subsequent clearing in xylene. In our collection, the average percentage of CNS-tissue shrinkage of bottlenose dolphin samples falls below 20% for any of the three dimensions of the tissue, but in terms of volume, the tissue may shrink by 40% on average. As all the herein investigated tissues were sampled and processed in the same way, shrinkage was not accounted for.

The accumulation of the above-mentioned effects can amount to variations in cell morphology, to which large sample numbers can be of help. Nonetheless, to this day, studies including rare, wild specimen samples seldom allow perfusion, hence results have to be taken considering those factors. Thus, further systematic studies including a larger sample size would greatly contribute to corroborating the present results.

Alternative approaches in cetaceans could include measuring cortical gray matter volume (Bajaj et al., 2017) in decomposition code 1 (fresh), formalin-fixed post-mortem brains, as an established, less invasive technique, with the advantage of giving an overview over a whole hemisphere. Interestingly, Roe et al. (2021) demonstrated that in humans, comparatively thicker cortical areas exhibit a relatively stronger reduction in volume than thinner ones, and that this happens asymmetrically among different brain regions. Hence, focusing on the relatively thicker A1 of dolphins is likely to yield the most significant difference when evaluating larger sample populations. In the meantime, immunohistological, *in situ* hybridization, and molecular techniques could help recognize age-related changes in brain neurochemistry.

## 5 Perspectives and concluding remarks

Overall, with the necessary caution, our results seem to point towards age-related changes in interneurons in upper cortical layers, which is coherent with the importance of these layers in the current thalamo-cortical input model for cetaceans (Cozzi et al., 2017) and how well these layers are conserved phylogenetically in mammals.

In particular, the functional significance of layer II in large-brained mammals, which comprises immature neurons (La Rosa et al., 2020) further validates the present results. Interestingly, the presence of these idle immature neurons at birth, which can activate later in life, would also alleviate the need for neuron number changes in the cortex, which do not seem to occur in other mammals significantly. Doublecortin has been used to find evidence of these idle cells throughout the lifespan of rodents (Benedetti et al., 2023) and humans (Li et al., 2023). Further consideration could be taken regarding the presence of Doublecortin in cetaceans, as it could represent a key in brain plasticity (Bonfanti et al., 2023).

Important thalamo-cortical inputs reaching A1 likely first reach layer II, and could receive inhibitory signals in layer I and II. In this view, the peculiar brain of cetaceans offers a very specific evolutionary example among mammals, regarding the interaction between size, neuron quantity and overall cognitive capacities. Further characterization of these layers, potentially including glial cells such as astrocytes, will help understand thoroughly the functional circuitry of the auditory cortex in cetaceans associated with cross-modal learning from behavioral data (Bruck and Pack, 2022).

The differential study of cell types in the cortical column through computer vision algorithms and multivariate statistics has yielded results in various species and brain areas (Corain et al., 2020; Graïc et al., 2021; Vadori et al., 2023). The progressive accumulation of data regarding separate ages, sex, species, and other factors will help identify key features in the cytoarchitecture of the central nervous system in various contexts, such as that of evolution, or pathophysiology. The addition of immunocytochemical information on interneuron populations would also add to the complexity. Robust sampling numbers, systematic counting (Schleicher et al., 2000) and repetitions would greatly benefit such efforts. This includes the standardization work being done at the cellular level (Luengo-Sanchez et al., 2015; Wang et al., 2020).

## Data availability statement

The raw data supporting the conclusions of this article will be made available by the authors, without undue reservation.

## Ethics statement

Ethical approval was not required for the study involving animals in accordance with the local legislation and institutional requirements because the animals used in this work were obtained by the Department of Comparative Biomedicine and Food Science of Padua for post-mortem necropsy following stranding of death in a zoological park. Under these circumstances, no ethical permission is required, as the animals were not part of any experiment.

## Author contributions

J-MG: Data curation, Formal analysis, Investigation, Project administration, Software, Visualization, Writing – original draft, Writing – review & editing. LC: Conceptualization, Data curation, Formal analysis, Investigation, Methodology, Writing – review & editing. LF: Conceptualization, Data curation, Formal analysis, Methodology, Writing – review & editing. VV: Data curation, Formal analysis, Methodology, Software, Visualization, Writing – review & editing. EG: Conceptualization, Formal analysis, Investigation, Resources, Software, Supervision, Visualization, Writing – review & editing. TG: Investigation, Methodology, Writing – review & editing. KO: Investigation, Methodology, Validation, Writing – review & editing. CC: Conceptualization, Data curation, Formal analysis, Investigation, Resources, Validation, Visualization, Writing – review & editing. BC: Conceptualization, Project administration, Resources, Supervision, Validation, Writing – review & editing. AP: Conceptualization, Data curation, Funding acquisition, Methodology, Project administration, Resources, Supervision, Validation, Writing – review & editing.

## Footnotes

^1^https://www.marinemammals.eu/index.php?lang=en
