# Supplementary material for: Age-related changes in the primary auditory cortex of newborn, adults and aging bottlenose dolphins (Tursiops truncatus) are located in the upper cortical layers
Source: Front Neuroanat. 2024 Jan 5;17:1330384. doi: 10.3389/fnana.2023.1330384 (PMC10796513; doi:10.3389/fnana.2023.1330384)
Supplement: Supplementary file 1 [file Data_Sheet_1.pdf]

Analysis Size

- Descriprives
  - means by subject
    - Area
    - MajorAxisLength
    - MinorAxisLength
    - Perimeter
  - Radar Plot
  - Density
  - Principal Components
    - Layer 1
    - Layer 2
    - Layer 3
    - Layer 4
    - Layer 5
    - Layer 6
- Inferential Analysis
  - Univariate
  - Cobined by aspects
  - Cobined by Shape and Layer
  - Cobined by Layer
  - Pairwise comparisons

Descriprives

means by subject

Area

Population	Shape	Layer_ID	mean	sd
Adult	ellipsoid	1	11.04	1.06
Calf	ellipsoid	1	10.52	2.89
Old	ellipsoid	1	15.54	2.18
Adult	ellipsoid	2	27.44	3.25
Calf	ellipsoid	2	24.62	6.86
Old	ellipsoid	2	30.24	6.52
Adult	ellipsoid	3	21.03	3.20
Calf	ellipsoid	3	25.47	4.17
Old	ellipsoid	3	26.97	5.43
Adult	ellipsoid	4	25.16	14.69
Calf	ellipsoid	4	27.67	9.56
Old	ellipsoid	4	24.34	4.37
Adult	ellipsoid	5	21.96	6.48
Calf	ellipsoid	5	23.97	2.81
Old	ellipsoid	5	22.66	3.39
Adult	ellipsoid	6	16.65	4.47
Calf	ellipsoid	6	18.67	3.27
Old	ellipsoid	6	21.09	4.84
Adult	round	1	9.59	1.02
Calf	round	1	6.92	1.48
Old	round	1	12.37	2.01
Adult	round	2	25.14	3.35
Calf	round	2	18.15	5.94

Population	Shape	Layer_ID	mean	sd
Old	round	2	27.34	2.33
Adult	round	3	21.97	4.73
Calf	round	3	21.14	5.19
Old	round	3	26.78	2.62
Adult	round	4	20.98	3.27
Calf	round	4	19.20	4.72
Old	round	4	24.26	2.88
Adult	round	5	19.31	4.35
Calf	round	5	18.78	2.02
Old	round	5	22.91	2.06
Adult	round	6	13.80	3.36
Calf	round	6	13.35	1.16
Old	round	6	17.31	1.83
Adult	pyramidal	1	16.79	1.68
Calf	pyramidal	1	18.31	4.02
Old	pyramidal	1	20.47	2.25
Adult	pyramidal	2	40.58	4.06
Calf	pyramidal	2	32.40	7.17
Old	pyramidal	2	41.53	4.20
Adult	pyramidal	3	34.49	4.44
Calf	pyramidal	3	34.41	5.37
Old	pyramidal	3	37.10	4.51
Adult	pyramidal	4	34.96	7.19
Calf	pyramidal	4	29.26	4.53
Old	pyramidal	4	36.71	7.04
Adult	pyramidal	5	39.94	5.25
Calf	pyramidal	5	32.08	5.76
Old	pyramidal	5	38.14	5.91
Adult	pyramidal	6	31.10	3.92
Calf	pyramidal	6	26.13	4.27
Old	pyramidal	6	32.38	6.64
Adult	complex	1	19.00	1.88
Calf	complex	1	16.55	3.39
Old	complex	1	18.14	2.04
Adult	complex	2	43.76	6.44
Calf	complex	2	32.83	6.86
Old	complex	2	40.25	5.13
Adult	complex	3	47.25	9.23
Calf	complex	3	39.02	4.62
Old	complex	3	44.21	5.54
Adult	complex	4	42.90	8.23
Calf	complex	4	37.58	4.79
Old	complex	4	43.80	11.46
Adult	complex	5	50.42	10.66
Calf	complex	5	37.47	2.77
Old	complex	5	48.13	7.00

Population	Shape	Layer_ID	mean	sd
Adult	complex	6	37.41	7.90
Calf	complex	6	29.60	6.39
Old	complex	6	37.23	5.56

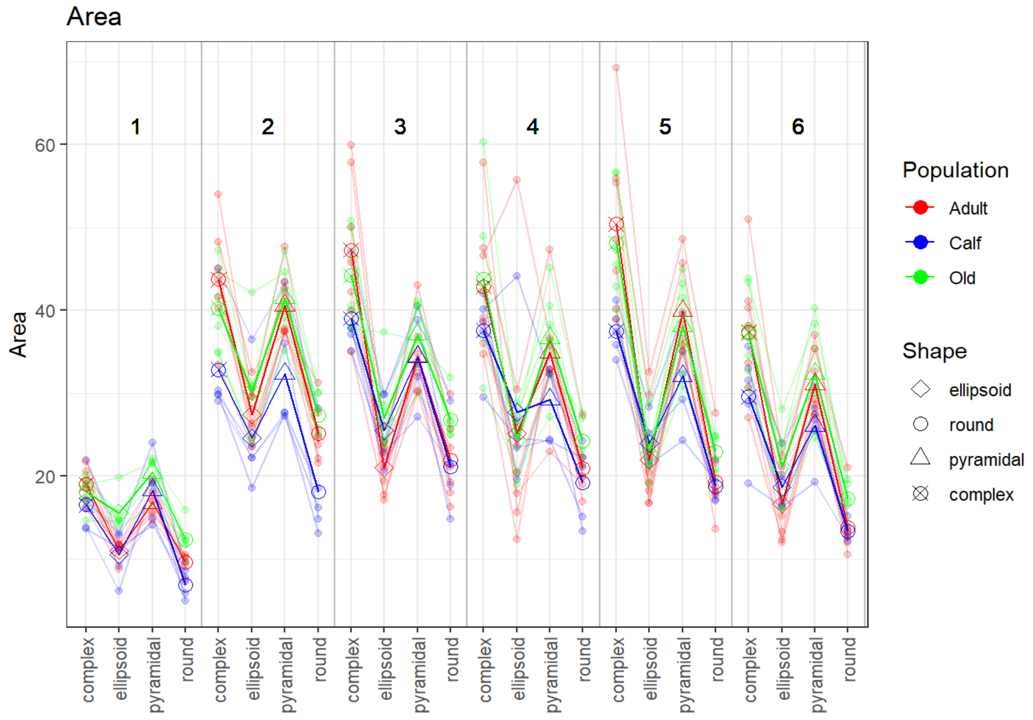

MajorAxisLength

Population	Shape	Layer_ID	mean	sd
Adult	ellipsoid	1	4.66	0.23
Calf	ellipsoid	1	4.44	0.63
Old	ellipsoid	1	5.49	0.31
Adult	ellipsoid	2	7.01	0.38
Calf	ellipsoid	2	6.74	0.81
Old	ellipsoid	2	7.51	0.83
Adult	ellipsoid	3	6.11	0.39
Calf	ellipsoid	3	6.83	0.46
Old	ellipsoid	3	7.02	0.72
Adult	ellipsoid	4	6.64	1.55
Calf	ellipsoid	4	7.17	1.40
Old	ellipsoid	4	6.77	0.63
Adult	ellipsoid	5	6.12	0.68
Calf	ellipsoid	5	6.46	0.35
Old	ellipsoid	5	6.39	0.48
Adult	ellipsoid	6	5.44	0.62
Calf	ellipsoid	6	5.83	0.49
Old	ellipsoid	6	6.25	0.73
Adult	round	1	3.83	0.22
Calf	round	1	3.16	0.33
Old	round	1	4.33	0.35
Adult	round	2	5.92	0.40
Calf	round	2	4.96	0.83
Old	round	2	6.38	0.28

Population	Shape	Layer_ID	mean	sd
Adult	round	3	5.42	0.49
Calf	round	3	5.32	0.65
Old	round	3	6.18	0.41
Adult	round	4	5.38	0.32
Calf	round	4	5.12	0.74
Old	round	4	5.91	0.37
Adult	round	5	4.98	0.37
Calf	round	5	4.94	0.26
Old	round	5	5.59	0.23
Adult	round	6	4.34	0.35
Calf	round	6	4.25	0.18
Old	round	6	4.94	0.22
Adult	pyramidal	1	6.92	0.44
Calf	pyramidal	1	7.26	0.73
Old	pyramidal	1	7.36	0.51
Adult	pyramidal	2	10.80	0.76
Calf	pyramidal	2	9.50	1.12
Old	pyramidal	2	10.93	0.74
Adult	pyramidal	3	9.64	0.57
Calf	pyramidal	3	9.52	0.82
Old	pyramidal	3	10.00	0.85
Adult	pyramidal	4	9.68	1.07
Calf	pyramidal	4	8.96	0.91
Old	pyramidal	4	9.99	1.16
Adult	pyramidal	5	10.23	0.43
Calf	pyramidal	5	9.17	0.90
Old	pyramidal	5	10.06	0.92
Adult	pyramidal	6	9.17	0.43
Calf	pyramidal	6	8.31	0.77
Old	pyramidal	6	9.19	0.91
Adult	complex	1	5.75	0.24
Calf	complex	1	5.25	0.53
Old	complex	1	5.69	0.35
Adult	complex	2	8.84	0.68
Calf	complex	2	7.65	0.71
Old	complex	2	8.51	0.59
Adult	complex	3	9.07	0.86
Calf	complex	3	8.33	0.48
Old	complex	3	8.79	0.59
Adult	complex	4	8.72	0.78
Calf	complex	4	8.20	0.60
Old	complex	4	8.77	1.07
Adult	complex	5	9.13	0.88
Calf	complex	5	8.13	0.37
Old	complex	5	9.07	0.72
Adult	complex	6	7.89	0.76

Population	Shape	Layer_ID	mean	sd
Calf	complex	6	7.11	0.88
Old	complex	6	8.02	0.55

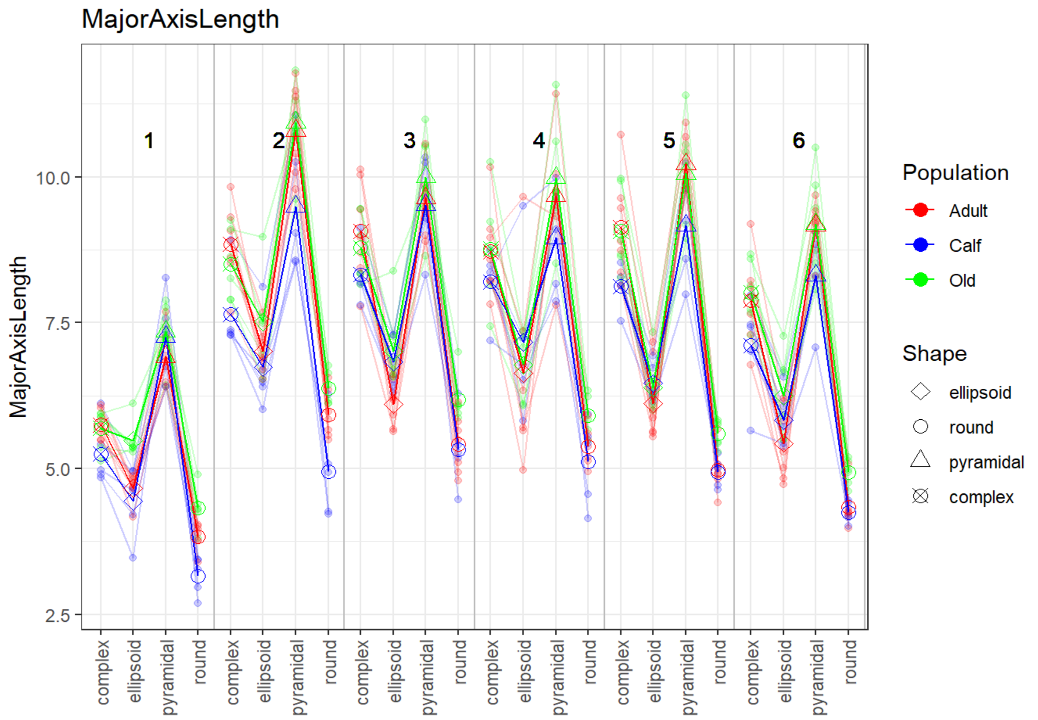

MinorAxisLength

Population	Shape	Layer_ID	mean	sd
Adult	ellipsoid	1	2.89	0.14
Calf	ellipsoid	1	2.75	0.39
Old	ellipsoid	1	3.39	0.21
Adult	ellipsoid	2	4.34	0.23
Calf	ellipsoid	2	4.19	0.52
Old	ellipsoid	2	4.65	0.48
Adult	ellipsoid	3	3.79	0.24
Calf	ellipsoid	3	4.25	0.30
Old	ellipsoid	3	4.36	0.47
Adult	ellipsoid	4	4.11	1.01
Calf	ellipsoid	4	4.46	0.93
Old	ellipsoid	4	4.22	0.38
Adult	ellipsoid	5	3.79	0.43
Calf	ellipsoid	5	4.03	0.23
Old	ellipsoid	5	3.96	0.31
Adult	ellipsoid	6	3.36	0.38
Calf	ellipsoid	6	3.60	0.30
Old	ellipsoid	6	3.85	0.45
Adult	round	1	3.03	0.16
Calf	round	1	2.55	0.26
Old	round	1	3.41	0.23
Adult	round	2	4.66	0.31
Calf	round	2	3.89	0.65
Old	round	2	4.97	0.21
Adult	round	3	4.28	0.40

Population	Shape	Layer_ID	mean	sd
Calf	round	3	4.19	0.54
Old	round	3	4.84	0.29
Adult	round	4	4.21	0.28
Calf	round	4	4.06	0.61
Old	round	4	4.69	0.33
Adult	round	5	3.93	0.31
Calf	round	5	3.93	0.22
Old	round	5	4.40	0.20
Adult	round	6	3.44	0.29
Calf	round	6	3.37	0.11
Old	round	6	3.89	0.17
Adult	pyramidal	1	3.07	0.09
Calf	pyramidal	1	3.17	0.38
Old	pyramidal	1	3.55	0.16
Adult	pyramidal	2	4.92	0.25
Calf	pyramidal	2	4.40	0.45
Old	pyramidal	2	5.05	0.27
Adult	pyramidal	3	4.51	0.31
Calf	pyramidal	3	4.58	0.39
Old	pyramidal	3	4.79	0.28
Adult	pyramidal	4	4.52	0.51
Calf	pyramidal	4	4.23	0.38
Old	pyramidal	4	4.69	0.39
Adult	pyramidal	5	4.67	0.33
Calf	pyramidal	5	4.37	0.40
Old	pyramidal	5	4.71	0.35
Adult	pyramidal	6	4.11	0.29
Calf	pyramidal	6	3.95	0.38
Old	pyramidal	6	4.39	0.53
Adult	complex	1	4.26	0.19
Calf	complex	1	3.97	0.50
Old	complex	1	4.22	0.22
Adult	complex	2	6.58	0.55
Calf	complex	2	5.63	0.59
Old	complex	2	6.32	0.42
Adult	complex	3	6.79	0.68
Calf	complex	3	6.17	0.41
Old	complex	3	6.55	0.46
Adult	complex	4	6.44	0.64
Calf	complex	4	6.05	0.41
Old	complex	4	6.54	0.97
Adult	complex	5	6.82	0.63
Calf	complex	5	6.04	0.26
Old	complex	5	6.78	0.53
Adult	complex	6	5.84	0.57
Calf	complex	6	5.28	0.68

Population	Shape	Layer_ID	mean	sd
Old	complex	6	5.93	0.43

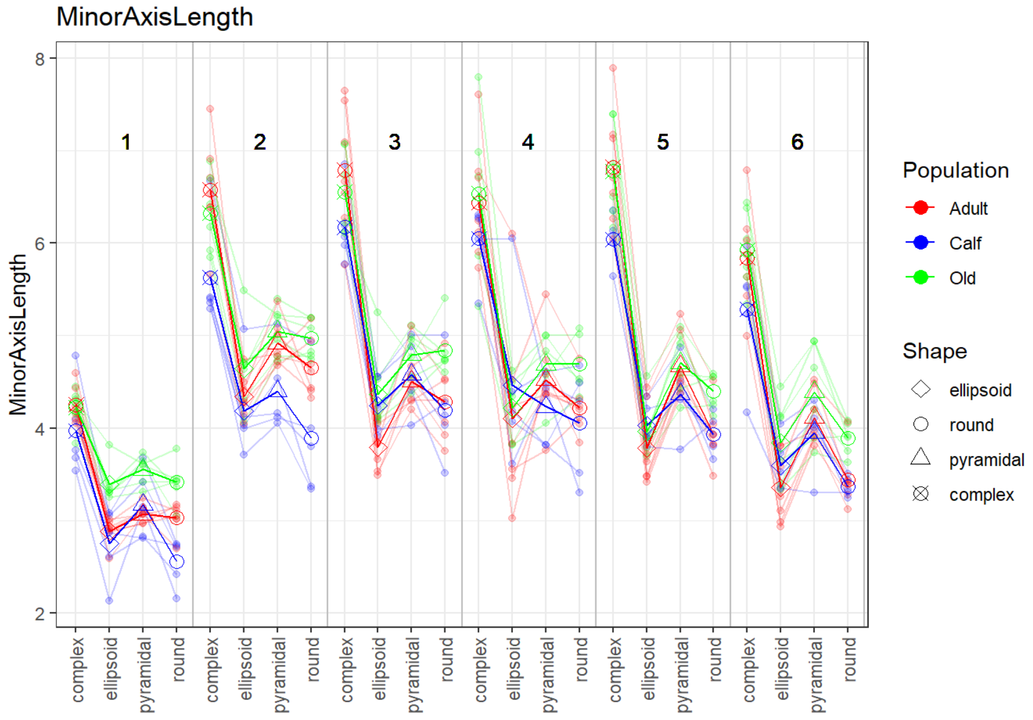

Perimeter

Population	Shape	Layer_ID	mean	sd
Adult	ellipsoid	1	11.89	0.63
Calf	ellipsoid	1	11.31	1.73
Old	ellipsoid	1	14.25	0.94
Adult	ellipsoid	2	18.71	1.22
Calf	ellipsoid	2	17.79	2.57
Old	ellipsoid	2	20.09	2.40
Adult	ellipsoid	3	16.00	1.15
Calf	ellipsoid	3	18.00	1.47
Old	ellipsoid	3	18.69	2.26
Adult	ellipsoid	4	17.47	4.44
Calf	ellipsoid	4	19.09	3.68
Old	ellipsoid	4	17.89	1.91
Adult	ellipsoid	5	16.14	2.09
Calf	ellipsoid	5	17.00	1.07
Old	ellipsoid	5	16.92	1.38
Adult	ellipsoid	6	14.14	1.86
Calf	ellipsoid	6	15.08	1.43
Old	ellipsoid	6	16.43	2.15
Adult	round	1	10.66	0.63
Calf	round	1	8.76	0.97
Old	round	1	12.17	0.98
Adult	round	2	17.30	1.25
Calf	round	2	14.27	2.61
Old	round	2	18.59	0.93
Adult	round	3	15.67	1.60
Calf	round	3	15.32	2.11

Population	Shape	Layer_ID	mean	sd
Old	round	3	17.93	1.25
Adult	round	4	15.44	1.07
Calf	round	4	14.68	2.22
Old	round	4	17.21	1.26
Adult	round	5	14.38	1.27
Calf	round	5	14.17	0.86
Old	round	5	16.21	0.69
Adult	round	6	12.32	1.16
Calf	round	6	12.00	0.55
Old	round	6	14.18	0.67
Adult	pyramidal	1	16.80	1.09
Calf	pyramidal	1	17.61	2.12
Old	pyramidal	1	18.39	1.26
Adult	pyramidal	2	27.79	2.04
Calf	pyramidal	2	23.86	3.12
Old	pyramidal	2	28.15	1.95
Adult	pyramidal	3	24.76	1.80
Calf	pyramidal	3	24.24	2.35
Old	pyramidal	3	25.76	2.29
Adult	pyramidal	4	24.70	3.18
Calf	pyramidal	4	22.67	2.44
Old	pyramidal	4	25.74	3.00
Adult	pyramidal	5	26.26	1.62
Calf	pyramidal	5	23.37	2.58
Old	pyramidal	5	25.77	2.54
Adult	pyramidal	6	23.04	1.15
Calf	pyramidal	6	20.81	2.29
Old	pyramidal	6	23.51	2.78
Adult	complex	1	17.16	0.89
Calf	complex	1	15.68	2.10
Old	complex	1	16.96	1.17
Adult	complex	2	27.59	2.45
Calf	complex	2	23.09	2.79
Old	complex	2	26.43	2.23
Adult	complex	3	28.16	3.09
Calf	complex	3	25.15	1.97
Old	complex	3	26.77	2.14
Adult	complex	4	26.62	2.56
Calf	complex	4	24.77	1.91
Old	complex	4	26.85	3.75
Adult	complex	5	28.67	3.34
Calf	complex	5	24.74	1.45
Old	complex	5	28.08	2.62
Adult	complex	6	24.23	2.65
Calf	complex	6	21.40	2.99
Old	complex	6	24.52	1.98

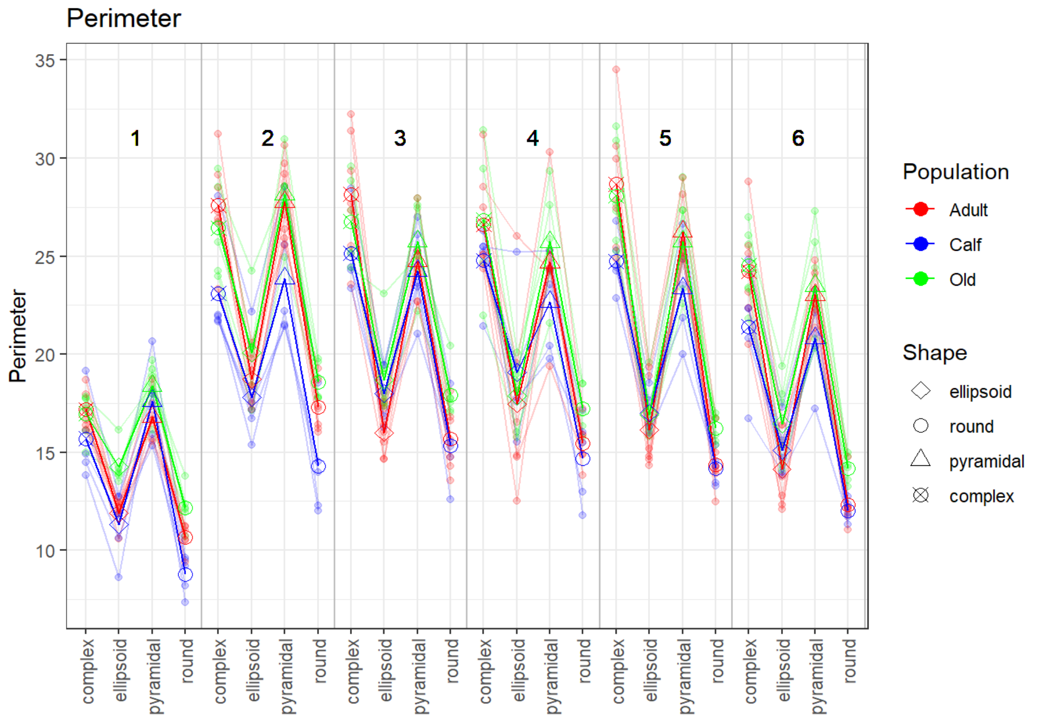

Radar Plot

Density

Principal Components

Layer 1

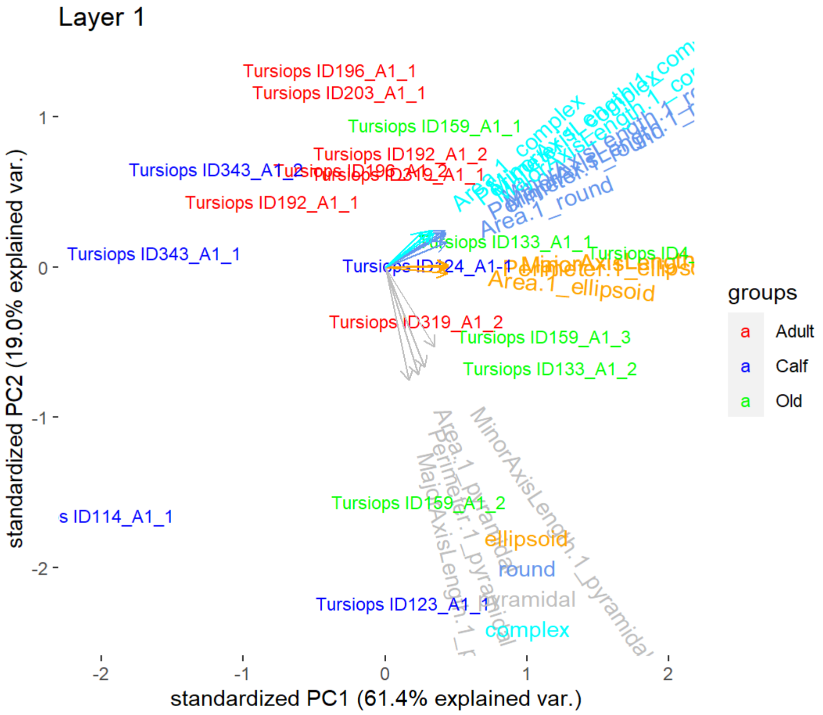

Layer 2

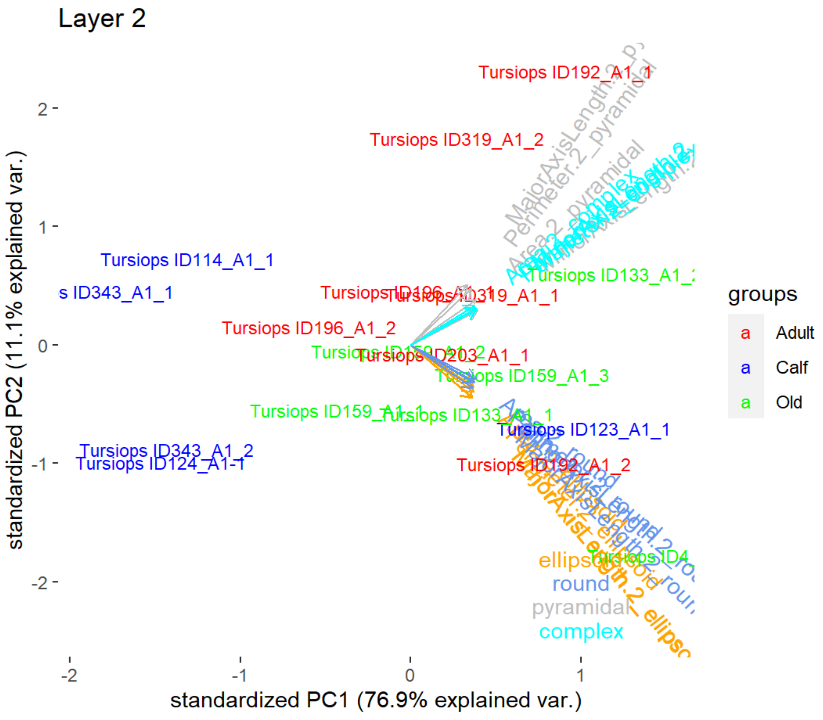

Layer 3

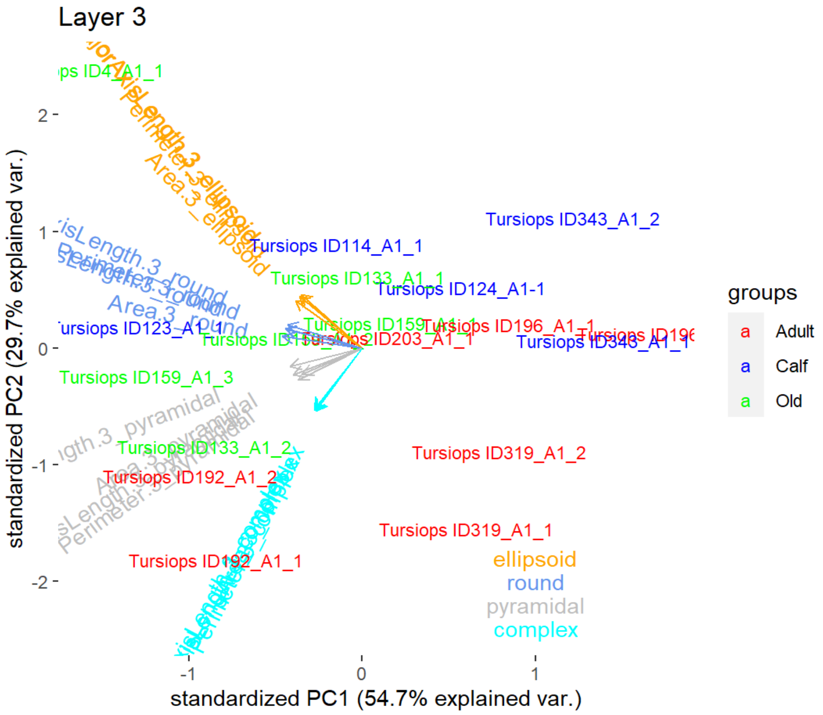

Layer 4

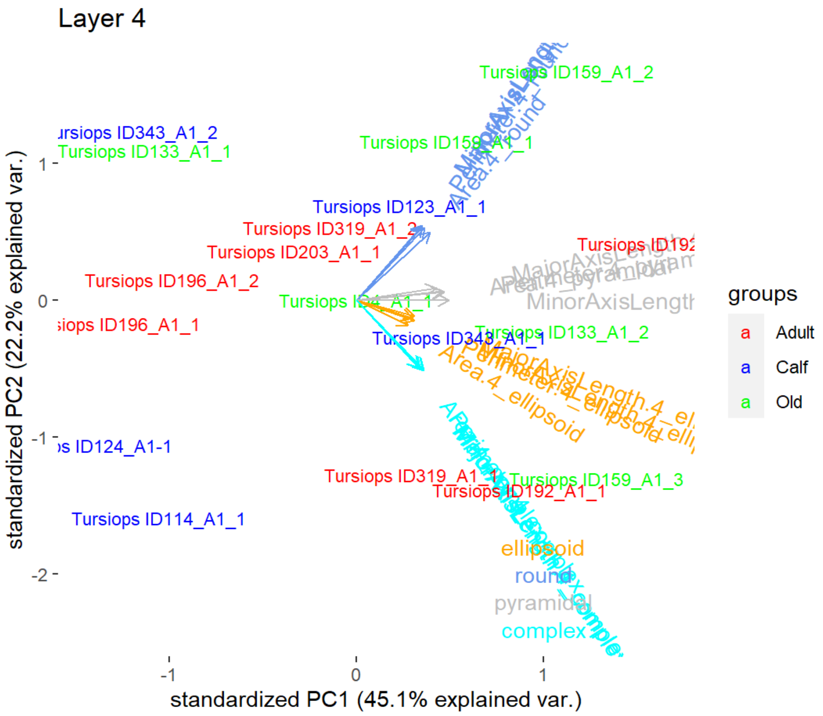

Layer 5

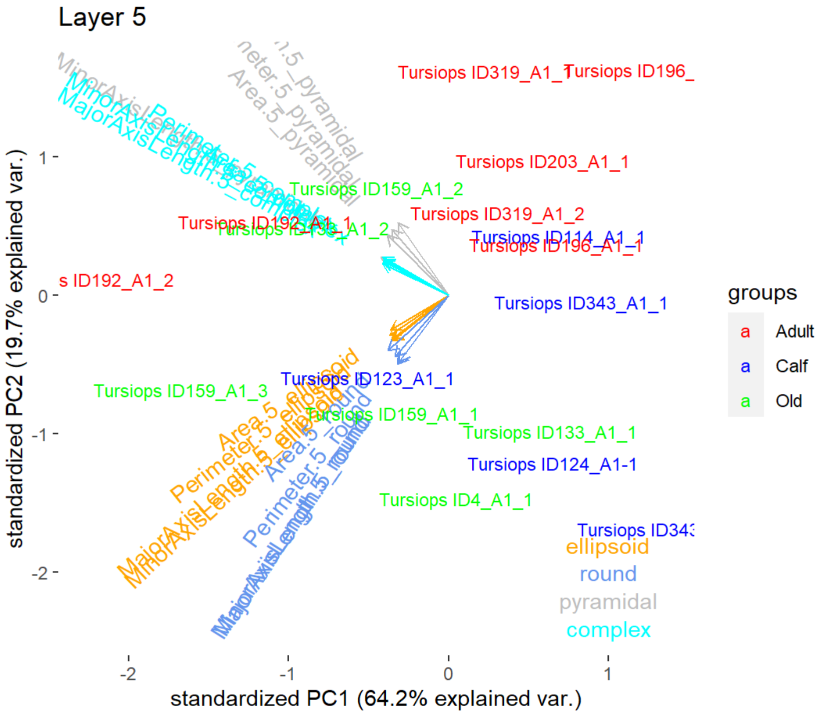

Layer 6

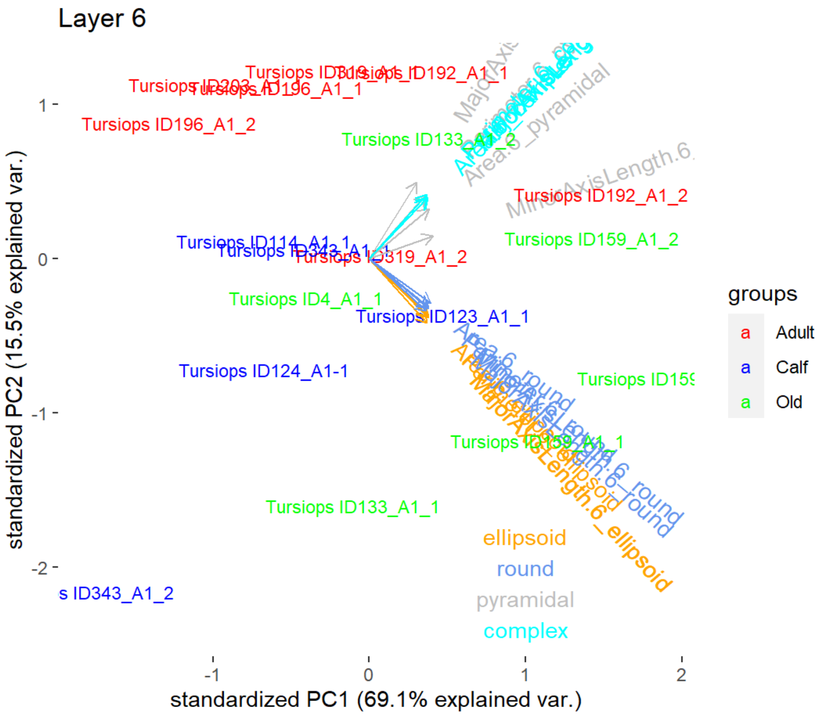

Inferential Analysis

Univariate

```
FALSE  Call:
FALSE  flip(Y = . ~ Population, data = DW, perms = nperms)
FALSE 4999 permutations.
FALSE
FALSE                                         Test    Stat tail
FALSE Area.1_ellipsoid_|_Population.Calf.             t -1.6811   ><
FALSE Area.1_ellipsoid_|_Population.Old.              t  4.5537   ><
FALSE MajorAxisLength.1_ellipsoid_|_Population.Calf.    t -2.0687   ><
FALSE MajorAxisLength.1_ellipsoid_|_Population.Old.     t  4.4804   ><
FALSE MinorAxisLength.1_ellipsoid_|_Population.Calf.    t -2.0370   ><
FALSE MinorAxisLength.1_ellipsoid_|_Population.Old.     t  4.3729   ><
FALSE Perimeter.1_ellipsoid_|_Population.Calf.          t -2.0356   ><
FALSE Perimeter.1_ellipsoid_|_Population.Old.           t  4.5424   ><
FALSE Area.2_ellipsoid_|_Population.Calf.             t -1.3725   ><
FALSE Area.2_ellipsoid_|_Population.Old.              t  1.3992   ><
FALSE MajorAxisLength.2_ellipsoid_|_Population.Calf.    t -1.3361   ><
FALSE MajorAxisLength.2_ellipsoid_|_Population.Old.     t  1.7958   ><
FALSE MinorAxisLength.2_ellipsoid_|_Population.Calf.    t -1.2870   ><
FALSE MinorAxisLength.2_ellipsoid_|_Population.Old.     t  1.7678   ><
FALSE Perimeter.2_ellipsoid_|_Population.Calf.          t -1.3703   ><
FALSE Perimeter.2_ellipsoid_|_Population.Old.           t  1.6780   ><
FALSE Area.3_ellipsoid_|_Population.Calf.             t  0.6349   ><
FALSE Area.3_ellipsoid_|_Population.Old.              t  1.7302   ><
FALSE MajorAxisLength.3_ellipsoid_|_Population.Calf.    t  0.8371   ><
FALSE MajorAxisLength.3_ellipsoid_|_Population.Old.     t  1.9519   ><
FALSE MinorAxisLength.3_ellipsoid_|_Population.Calf.    t  0.8378   ><
FALSE MinorAxisLength.3_ellipsoid_|_Population.Old.     t  1.9165   ><
FALSE Perimeter.3_ellipsoid_|_Population.Calf.          t  0.6941   ><
FALSE Perimeter.3_ellipsoid_|_Population.Old.           t  1.9571   ><
FALSE Area.4_ellipsoid_|_Population.Calf.             t  0.4921   ><
FALSE Area.4_ellipsoid_|_Population.Old.              t -0.3058   ><
FALSE MajorAxisLength.4_ellipsoid_|_Population.Calf.    t  0.7017   ><
FALSE MajorAxisLength.4_ellipsoid_|_Population.Old.     t -0.1180   ><
FALSE MinorAxisLength.4_ellipsoid_|_Population.Calf.    t  0.6957   ><
FALSE MinorAxisLength.4_ellipsoid_|_Population.Old.     t -0.0709   ><
FALSE Perimeter.4_ellipsoid_|_Population.Calf.          t  0.7585   ><
FALSE Perimeter.4_ellipsoid_|_Population.Old.           t -0.1260   ><
FALSE Area.5_ellipsoid_|_Population.Calf.             t  0.6714   ><
FALSE Area.5_ellipsoid_|_Population.Old.              t -0.0563   ><
FALSE MajorAxisLength.5_ellipsoid_|_Population.Calf.    t  0.7440   ><
FALSE MajorAxisLength.5_ellipsoid_|_Population.Old.     t  0.4569   ><
FALSE MinorAxisLength.5_ellipsoid_|_Population.Calf.    t  0.8600   ><
FALSE MinorAxisLength.5_ellipsoid_|_Population.Old.     t  0.4262   ><
FALSE Perimeter.5_ellipsoid_|_Population.Calf.          t  0.5724   ><
FALSE Perimeter.5_ellipsoid_|_Population.Old.           t  0.4982   ><
FALSE Area.6_ellipsoid_|_Population.Calf.             t -0.0129   ><
FALSE Area.6_ellipsoid_|_Population.Old.              t  1.6336   ><
FALSE MajorAxisLength.6_ellipsoid_|_Population.Calf.    t  0.0536   ><
FALSE MajorAxisLength.6_ellipsoid_|_Population.Old.     t  1.9828   ><
FALSE MinorAxisLength.6_ellipsoid_|_Population.Calf.    t  0.0753   ><
FALSE MinorAxisLength.6_ellipsoid_|_Population.Old.     t  1.9506   ><
FALSE Perimeter.6_ellipsoid_|_Population.Calf.          t -0.1066   ><
FALSE Perimeter.6_ellipsoid_|_Population.Old.           t  1.9953   ><
FALSE Area.1_round_|_Population.Calf.                 t -3.7611   ><
FALSE Area.1_round_|_Population.Old.                  t  4.0326   ><
FALSE MajorAxisLength.1_round_|_Population.Calf.        t -4.5405   ><
FALSE MajorAxisLength.1_round_|_Population.Old.         t  3.7151   ><
FALSE MinorAxisLength.1_round_|_Population.Calf.        t -4.4636   ><
FALSE MinorAxisLength.1_round_|_Population.Old.         t  3.8738   ><
FALSE Perimeter.1_round_|_Population.Calf.              t -4.4666   ><
FALSE Perimeter.1_round_|_Population.Old.               t  3.8569   ><
FALSE Area.2_round_|_Population.Calf.                 t -3.7257   ><
FALSE Area.2_round_|_Population.Old.                  t  2.0394   ><
FALSE MajorAxisLength.2_round_|_Population.Calf.        t -3.9663   ><
FALSE MajorAxisLength.2_round_|_Population.Old.         t  2.5308   ><
FALSE MinorAxisLength.2_round_|_Population.Calf.        t -4.0127   ><
FALSE MinorAxisLength.2_round_|_Population.Old.         t  2.3927   ><
FALSE Perimeter.2_round_|_Population.Calf.              t -3.9278   ><
FALSE Perimeter.2_round_|_Population.Old.               t  2.4022   ><
FALSE Area.3_round_|_Population.Calf.                 t -1.2033   ><
FALSE Area.3_round_|_Population.Old.                  t  2.4026   ><
FALSE MajorAxisLength.3_round_|_Population.Calf.        t -1.3699   ><
FALSE MajorAxisLength.3_round_|_Population.Old.         t  3.1063   ><
FALSE MinorAxisLength.3_round_|_Population.Calf.        t -1.3657   ><
FALSE MinorAxisLength.3_round_|_Population.Old.         t  2.8799   ><
FALSE Perimeter.3_round_|_Population.Calf.              t -1.3616   ><
FALSE Perimeter.3_round_|_Population.Old.               t  2.9067   ><
FALSE Area.4_round_|_Population.Calf.                 t -1.5348   ><
```

```
FALSE Area.4_round_|_Population.Old.                    t  1.9388   ><
FALSE MajorAxisLength.4_round_|_Population.Calf.        t -1.6840   ><
FALSE MajorAxisLength.4_round_|_Population.Old.         t  2.2727   ><
FALSE MinorAxisLength.4_round_|_Population.Calf.        t -1.4702   ><
FALSE MinorAxisLength.4_round_|_Population.Old.         t  2.3021   ><
FALSE Perimeter.4_round_|_Population.Calf.              t -1.6596   ><
FALSE Perimeter.4_round_|_Population.Old.               t  2.3735   ><
FALSE Area.5_round_|_Population.Calf.                   t -1.1634   ><
FALSE Area.5_round_|_Population.Old.                    t  2.4089   ><
FALSE MajorAxisLength.5_round_|_Population.Calf.        t -1.4867   ><
FALSE MajorAxisLength.5_round_|_Population.Old.         t  4.1849   ><
FALSE MinorAxisLength.5_round_|_Population.Calf.        t -1.2264   ><
FALSE MinorAxisLength.5_round_|_Population.Old.         t  3.6707   ><
FALSE Perimeter.5_round_|_Population.Calf.              t -1.5284   ><
FALSE Perimeter.5_round_|_Population.Old.               t  3.8164   ><
FALSE Area.6_round_|_Population.Calf.                   t -1.3412   ><
FALSE Area.6_round_|_Population.Old.                    t  3.0143   ><
FALSE MajorAxisLength.6_round_|_Population.Calf.        t -1.7724   ><
FALSE MajorAxisLength.6_round_|_Population.Old.         t  4.6426   ><
FALSE MinorAxisLength.6_round_|_Population.Calf.        t -1.7827   ><
FALSE MinorAxisLength.6_round_|_Population.Old.         t  4.3087   ><
FALSE Perimeter.6_round_|_Population.Calf.              t -1.8191   ><
FALSE Perimeter.6_round_|_Population.Old.               t  4.5031   ><
FALSE Area.1_pyramidal_|_Population.Calf.               t -0.1096   ><
FALSE Area.1_pyramidal_|_Population.Old.                t  2.2185   ><
FALSE MajorAxisLength.1_pyramidal_|_Population.Calf.    t  0.4443   ><
FALSE MajorAxisLength.1_pyramidal_|_Population.Old.     t  1.0266   ><
FALSE MinorAxisLength.1_pyramidal_|_Population.Calf.    t -0.7171   ><
FALSE MinorAxisLength.1_pyramidal_|_Population.Old.     t  3.8761   ><
FALSE Perimeter.1_pyramidal_|_Population.Calf.          t  0.0837   ><
FALSE Perimeter.1_pyramidal_|_Population.Old.           t  1.6416   ><
FALSE Area.2_pyramidal_|_Population.Calf.               t -3.1978   ><
FALSE Area.2_pyramidal_|_Population.Old.                t  1.3942   ><
FALSE MajorAxisLength.2_pyramidal_|_Population.Calf.    t -3.0061   ><
FALSE MajorAxisLength.2_pyramidal_|_Population.Old.     t  1.3096   ><
FALSE MinorAxisLength.2_pyramidal_|_Population.Calf.    t -3.3915   ><
FALSE MinorAxisLength.2_pyramidal_|_Population.Old.     t  1.7490   ><
FALSE Perimeter.2_pyramidal_|_Population.Calf.          t -3.3121   ><
FALSE Perimeter.2_pyramidal_|_Population.Old.           t  1.3753   ><
FALSE Area.3_pyramidal_|_Population.Calf.               t -0.5010   ><
FALSE Area.3_pyramidal_|_Population.Old.                t  1.1223   ><
FALSE MajorAxisLength.3_pyramidal_|_Population.Calf.    t -0.7069   ><
FALSE MajorAxisLength.3_pyramidal_|_Population.Old.     t  1.0895   ><
FALSE MinorAxisLength.3_pyramidal_|_Population.Calf.    t -0.3136   ><
FALSE MinorAxisLength.3_pyramidal_|_Population.Old.     t  1.5641   ><
FALSE Perimeter.3_pyramidal_|_Population.Calf.          t -0.8587   ><
FALSE Perimeter.3_pyramidal_|_Population.Old.           t  1.1416   ><
FALSE Area.4_pyramidal_|_Population.Calf.               t -1.8866   ><
FALSE Area.4_pyramidal_|_Population.Old.                t  1.0738   ><
FALSE MajorAxisLength.4_pyramidal_|_Population.Calf.    t -1.5372   ><
FALSE MajorAxisLength.4_pyramidal_|_Population.Old.     t  1.0122   ><
FALSE MinorAxisLength.4_pyramidal_|_Population.Calf.    t -1.5429   ><
FALSE MinorAxisLength.4_pyramidal_|_Population.Old.     t  1.1294   ><
FALSE Perimeter.4_pyramidal_|_Population.Calf.          t -1.5926   ><
FALSE Perimeter.4_pyramidal_|_Population.Old.           t  1.1149   ><
FALSE Area.5_pyramidal_|_Population.Calf.               t -2.3573   ><
FALSE Area.5_pyramidal_|_Population.Old.                t  0.4489   ><
FALSE MajorAxisLength.5_pyramidal_|_Population.Calf.    t -2.4467   ><
FALSE MajorAxisLength.5_pyramidal_|_Population.Old.     t  0.6170   ><
FALSE MinorAxisLength.5_pyramidal_|_Population.Calf.    t -1.7088   ><
FALSE MinorAxisLength.5_pyramidal_|_Population.Old.     t  0.8454   ><
FALSE Perimeter.5_pyramidal_|_Population.Calf.          t -2.2640   ><
FALSE Perimeter.5_pyramidal_|_Population.Old.           t  0.5567   ><
FALSE Area.6_pyramidal_|_Population.Calf.               t -2.0745   ><
FALSE Area.6_pyramidal_|_Population.Old.                t  1.2148   ><
FALSE MajorAxisLength.6_pyramidal_|_Population.Calf.    t -2.3237   ><
FALSE MajorAxisLength.6_pyramidal_|_Population.Old.     t  0.9444   ><
FALSE MinorAxisLength.6_pyramidal_|_Population.Calf.    t -1.3006   ><
FALSE MinorAxisLength.6_pyramidal_|_Population.Old.     t  1.6818   ><
FALSE Perimeter.6_pyramidal_|_Population.Calf.          t -2.1779   ><
FALSE Perimeter.6_pyramidal_|_Population.Old.           t  1.1969   ><
FALSE Area.1_complex_|_Population.Calf.                 t -1.5929   ><
FALSE Area.1_complex_|_Population.Old.                  t  0.1175   ><
FALSE MajorAxisLength.1_complex_|_Population.Calf.      t -2.4008   ><
FALSE MajorAxisLength.1_complex_|_Population.Old.       t  0.6884   ><
FALSE MinorAxisLength.1_complex_|_Population.Calf.      t -1.6654   ><
FALSE MinorAxisLength.1_complex_|_Population.Old.       t  0.5293   ><
FALSE Perimeter.1_complex_|_Population.Calf.            t -1.8855   ><
```

```
FALSE Perimeter.1_complex_|_Population.Old.            t  0.5415   ><
FALSE Area.2_complex_|_Population.Calf.                t -2.7832   ><
FALSE Area.2_complex_|_Population.Old.                 t  0.2672   ><
FALSE MajorAxisLength.2_complex_|_Population.Calf.     t -2.9207   ><
FALSE MajorAxisLength.2_complex_|_Population.Old.      t  0.3859   ><
FALSE MinorAxisLength.2_complex_|_Population.Calf.     t -2.9767   ><
FALSE MinorAxisLength.2_complex_|_Population.Old.      t  0.4336   ><
FALSE Perimeter.2_complex_|_Population.Calf.           t -2.9799   ><
FALSE Perimeter.2_complex_|_Population.Old.            t  0.4542   ><
FALSE Area.3_complex_|_Population.Calf.                t -1.8030   ><
FALSE Area.3_complex_|_Population.Old.                 t  0.0980   ><
FALSE MajorAxisLength.3_complex_|_Population.Calf.     t -1.6683   ><
FALSE MajorAxisLength.3_complex_|_Population.Old.      t  0.0716   ><
FALSE MinorAxisLength.3_complex_|_Population.Calf.     t -1.7297   ><
FALSE MinorAxisLength.3_complex_|_Population.Old.      t  0.0681   ><
FALSE Perimeter.3_complex_|_Population.Calf.           t -1.7310   ><
FALSE Perimeter.3_complex_|_Population.Old.            t -0.0973   ><
FALSE Area.4_complex_|_Population.Calf.                t -1.2790   ><
FALSE Area.4_complex_|_Population.Old.                 t  0.6402   ><
FALSE MajorAxisLength.4_complex_|_Population.Calf.     t -1.2559   ><
FALSE MajorAxisLength.4_complex_|_Population.Old.      t  0.5542   ><
FALSE MinorAxisLength.4_complex_|_Population.Calf.     t -1.1733   ><
FALSE MinorAxisLength.4_complex_|_Population.Old.      t  0.6527   ><
FALSE Perimeter.4_complex_|_Population.Calf.           t -1.3302   ><
FALSE Perimeter.4_complex_|_Population.Old.            t  0.6239   ><
FALSE Area.5_complex_|_Population.Calf.                t -2.8076   ><
FALSE Area.5_complex_|_Population.Old.                 t  0.6332   ><
FALSE MajorAxisLength.5_complex_|_Population.Calf.     t -2.5835   ><
FALSE MajorAxisLength.5_complex_|_Population.Old.      t  0.8403   ><
FALSE MinorAxisLength.5_complex_|_Population.Calf.     t -2.7822   ><
FALSE MinorAxisLength.5_complex_|_Population.Old.      t  0.9014   ><
FALSE Perimeter.5_complex_|_Population.Calf.           t -2.5676   ><
FALSE Perimeter.5_complex_|_Population.Old.            t  0.6571   ><
FALSE Area.6_complex_|_Population.Calf.                t -2.1642   ><
FALSE Area.6_complex_|_Population.Old.                 t  0.8068   ><
FALSE MajorAxisLength.6_complex_|_Population.Calf.     t -2.1569   ><
FALSE MajorAxisLength.6_complex_|_Population.Old.      t  1.1207   ><
FALSE MinorAxisLength.6_complex_|_Population.Calf.     t -2.0275   ><
FALSE MinorAxisLength.6_complex_|_Population.Old.      t  1.0421   ><
FALSE Perimeter.6_complex_|_Population.Calf.           t -2.2109   ><
FALSE Perimeter.6_complex_|_Population.Old.            t  1.0387   ><
FALSE                                              p-value sig.
FALSE Area.1_ellipsoid_|_Population.Calf.             0.1066
FALSE Area.1_ellipsoid_|_Population.Old.              0.0002  ***
FALSE MajorAxisLength.1_ellipsoid_|_Population.Calf.  0.0440    *
FALSE MajorAxisLength.1_ellipsoid_|_Population.Old.   0.0004  ***
FALSE MinorAxisLength.1_ellipsoid_|_Population.Calf.  0.0470    *
FALSE MinorAxisLength.1_ellipsoid_|_Population.Old.   0.0004  ***
FALSE Perimeter.1_ellipsoid_|_Population.Calf.        0.0492    *
FALSE Perimeter.1_ellipsoid_|_Population.Old.         0.0004  ***
FALSE Area.2_ellipsoid_|_Population.Calf.             0.1806
FALSE Area.2_ellipsoid_|_Population.Old.              0.1726
FALSE MajorAxisLength.2_ellipsoid_|_Population.Calf.  0.1920
FALSE MajorAxisLength.2_ellipsoid_|_Population.Old.   0.0824
FALSE MinorAxisLength.2_ellipsoid_|_Population.Calf.  0.2056
FALSE MinorAxisLength.2_ellipsoid_|_Population.Old.   0.0888
FALSE Perimeter.2_ellipsoid_|_Population.Calf.        0.1836
FALSE Perimeter.2_ellipsoid_|_Population.Old.         0.1052
FALSE Area.3_ellipsoid_|_Population.Calf.             0.5232
FALSE Area.3_ellipsoid_|_Population.Old.              0.0870
FALSE MajorAxisLength.3_ellipsoid_|_Population.Calf.  0.4058
FALSE MajorAxisLength.3_ellipsoid_|_Population.Old.   0.0530
FALSE MinorAxisLength.3_ellipsoid_|_Population.Calf.  0.4108
FALSE MinorAxisLength.3_ellipsoid_|_Population.Old.   0.0552
FALSE Perimeter.3_ellipsoid_|_Population.Calf.        0.4876
FALSE Perimeter.3_ellipsoid_|_Population.Old.         0.0500    *
FALSE Area.4_ellipsoid_|_Population.Calf.             0.6728
FALSE Area.4_ellipsoid_|_Population.Old.              0.7888
FALSE MajorAxisLength.4_ellipsoid_|_Population.Calf.  0.5014
FALSE MajorAxisLength.4_ellipsoid_|_Population.Old.   0.9128
FALSE MinorAxisLength.4_ellipsoid_|_Population.Calf.  0.5100
FALSE MinorAxisLength.4_ellipsoid_|_Population.Old.   0.9470
FALSE Perimeter.4_ellipsoid_|_Population.Calf.        0.4588
FALSE Perimeter.4_ellipsoid_|_Population.Old.         0.8980
FALSE Area.5_ellipsoid_|_Population.Calf.             0.5036
FALSE Area.5_ellipsoid_|_Population.Old.              0.9572
FALSE MajorAxisLength.5_ellipsoid_|_Population.Calf.  0.4560
FALSE MajorAxisLength.5_ellipsoid_|_Population.Old.   0.6480
```

```
FALSE MinorAxisLength.5_ellipsoid_|_Population.Calf.  0.3890
FALSE MinorAxisLength.5_ellipsoid_|_Population.Old.   0.6716
FALSE Perimeter.5_ellipsoid_|_Population.Calf.        0.5616
FALSE Perimeter.5_ellipsoid_|_Population.Old.         0.6226
FALSE Area.6_ellipsoid_|_Population.Calf.             0.9870
FALSE Area.6_ellipsoid_|_Population.Old.              0.1140
FALSE MajorAxisLength.6_ellipsoid_|_Population.Calf.  0.9518
FALSE MajorAxisLength.6_ellipsoid_|_Population.Old.   0.0556
FALSE MinorAxisLength.6_ellipsoid_|_Population.Calf.  0.9346
FALSE MinorAxisLength.6_ellipsoid_|_Population.Old.   0.0608
FALSE Perimeter.6_ellipsoid_|_Population.Calf.        0.9138
FALSE Perimeter.6_ellipsoid_|_Population.Old.         0.0546
FALSE Area.1_round_|_Population.Calf.                 0.0004  ***
FALSE Area.1_round_|_Population.Old.                  0.0006  ***
FALSE MajorAxisLength.1_round_|_Population.Calf.      0.0004  ***
FALSE MajorAxisLength.1_round_|_Population.Old.       0.0024   **
FALSE MinorAxisLength.1_round_|_Population.Calf.      0.0004  ***
FALSE MinorAxisLength.1_round_|_Population.Old.       0.0024   **
FALSE Perimeter.1_round_|_Population.Calf.            0.0004  ***
FALSE Perimeter.1_round_|_Population.Old.             0.0022   **
FALSE Area.2_round_|_Population.Calf.                 0.0020   **
FALSE Area.2_round_|_Population.Old.                  0.0572
FALSE MajorAxisLength.2_round_|_Population.Calf.      0.0018   **
FALSE MajorAxisLength.2_round_|_Population.Old.       0.0204    *
FALSE MinorAxisLength.2_round_|_Population.Calf.      0.0018   **
FALSE MinorAxisLength.2_round_|_Population.Old.       0.0276    *
FALSE Perimeter.2_round_|_Population.Calf.            0.0018   **
FALSE Perimeter.2_round_|_Population.Old.             0.0280    *
FALSE Area.3_round_|_Population.Calf.                 0.2366
FALSE Area.3_round_|_Population.Old.                  0.0272    *
FALSE MajorAxisLength.3_round_|_Population.Calf.      0.1816
FALSE MajorAxisLength.3_round_|_Population.Old.       0.0068   **
FALSE MinorAxisLength.3_round_|_Population.Calf.      0.1852
FALSE MinorAxisLength.3_round_|_Population.Old.       0.0104    *
FALSE Perimeter.3_round_|_Population.Calf.            0.1842
FALSE Perimeter.3_round_|_Population.Old.             0.0096   **
FALSE Area.4_round_|_Population.Calf.                 0.1434
FALSE Area.4_round_|_Population.Old.                  0.0650
FALSE MajorAxisLength.4_round_|_Population.Calf.      0.1000
FALSE MajorAxisLength.4_round_|_Population.Old.       0.0316    *
FALSE MinorAxisLength.4_round_|_Population.Calf.      0.1548
FALSE MinorAxisLength.4_round_|_Population.Old.       0.0316    *
FALSE Perimeter.4_round_|_Population.Calf.            0.1072
FALSE Perimeter.4_round_|_Population.Old.             0.0276    *
FALSE Area.5_round_|_Population.Calf.                 0.2566
FALSE Area.5_round_|_Population.Old.                  0.0214    *
FALSE MajorAxisLength.5_round_|_Population.Calf.      0.1494
FALSE MajorAxisLength.5_round_|_Population.Old.       0.0004  ***
FALSE MinorAxisLength.5_round_|_Population.Calf.      0.2248
FALSE MinorAxisLength.5_round_|_Population.Old.       0.0016   **
FALSE Perimeter.5_round_|_Population.Calf.            0.1422
FALSE Perimeter.5_round_|_Population.Old.             0.0012   **
FALSE Area.6_round_|_Population.Calf.                 0.1880
FALSE Area.6_round_|_Population.Old.                  0.0058   **
FALSE MajorAxisLength.6_round_|_Population.Calf.      0.0892
FALSE MajorAxisLength.6_round_|_Population.Old.       0.0008  ***
FALSE MinorAxisLength.6_round_|_Population.Calf.      0.0894
FALSE MinorAxisLength.6_round_|_Population.Old.       0.0014   **
FALSE Perimeter.6_round_|_Population.Calf.            0.0812
FALSE Perimeter.6_round_|_Population.Old.             0.0010  ***
FALSE Area.1_pyramidal_|_Population.Calf.             0.9100
FALSE Area.1_pyramidal_|_Population.Old.              0.0324    *
FALSE MajorAxisLength.1_pyramidal_|_Population.Calf.  0.6602
FALSE MajorAxisLength.1_pyramidal_|_Population.Old.   0.3012
FALSE MinorAxisLength.1_pyramidal_|_Population.Calf.  0.4718
FALSE MinorAxisLength.1_pyramidal_|_Population.Old.   0.0014   **
FALSE Perimeter.1_pyramidal_|_Population.Calf.        0.9384
FALSE Perimeter.1_pyramidal_|_Population.Old.         0.1082
FALSE Area.2_pyramidal_|_Population.Calf.             0.0056   **
FALSE Area.2_pyramidal_|_Population.Old.              0.1796
FALSE MajorAxisLength.2_pyramidal_|_Population.Calf.  0.0096   **
FALSE MajorAxisLength.2_pyramidal_|_Population.Old.   0.2018
FALSE MinorAxisLength.2_pyramidal_|_Population.Calf.  0.0044   **
FALSE MinorAxisLength.2_pyramidal_|_Population.Old.   0.0988
FALSE Perimeter.2_pyramidal_|_Population.Calf.        0.0058   **
FALSE Perimeter.2_pyramidal_|_Population.Old.         0.1856
FALSE Area.3_pyramidal_|_Population.Calf.             0.6084
FALSE Area.3_pyramidal_|_Population.Old.              0.2630
```

```
FALSE MajorAxisLength.3_pyramidal_|_Population.Calf.  0.4856
FALSE MajorAxisLength.3_pyramidal_|_Population.Old.   0.2714
FALSE MinorAxisLength.3_pyramidal_|_Population.Calf.  0.7394
FALSE MinorAxisLength.3_pyramidal_|_Population.Old.   0.1284
FALSE Perimeter.3_pyramidal_|_Population.Calf.        0.3982
FALSE Perimeter.3_pyramidal_|_Population.Old.         0.2516
FALSE Area.4_pyramidal_|_Population.Calf.             0.0720
FALSE Area.4_pyramidal_|_Population.Old.              0.2928
FALSE MajorAxisLength.4_pyramidal_|_Population.Calf.  0.1334
FALSE MajorAxisLength.4_pyramidal_|_Population.Old.   0.3122
FALSE MinorAxisLength.4_pyramidal_|_Population.Calf.  0.1352
FALSE MinorAxisLength.4_pyramidal_|_Population.Old.   0.2622
FALSE Perimeter.4_pyramidal_|_Population.Calf.        0.1242
FALSE Perimeter.4_pyramidal_|_Population.Old.         0.2678
FALSE Area.5_pyramidal_|_Population.Calf.             0.0318    *
FALSE Area.5_pyramidal_|_Population.Old.              0.6546
FALSE MajorAxisLength.5_pyramidal_|_Population.Calf.  0.0236    *
FALSE MajorAxisLength.5_pyramidal_|_Population.Old.   0.5416
FALSE MinorAxisLength.5_pyramidal_|_Population.Calf.  0.0934
FALSE MinorAxisLength.5_pyramidal_|_Population.Old.   0.3852
FALSE Perimeter.5_pyramidal_|_Population.Calf.        0.0380    *
FALSE Perimeter.5_pyramidal_|_Population.Old.         0.5764
FALSE Area.6_pyramidal_|_Population.Calf.             0.0512
FALSE Area.6_pyramidal_|_Population.Old.              0.2352
FALSE MajorAxisLength.6_pyramidal_|_Population.Calf.  0.0286    *
FALSE MajorAxisLength.6_pyramidal_|_Population.Old.   0.3532
FALSE MinorAxisLength.6_pyramidal_|_Population.Calf.  0.1952
FALSE MinorAxisLength.6_pyramidal_|_Population.Old.   0.1022
FALSE Perimeter.6_pyramidal_|_Population.Calf.        0.0412    *
FALSE Perimeter.6_pyramidal_|_Population.Old.         0.2438
FALSE Area.1_complex_|_Population.Calf.               0.1180
FALSE Area.1_complex_|_Population.Old.                0.9060
FALSE MajorAxisLength.1_complex_|_Population.Calf.    0.0242    *
FALSE MajorAxisLength.1_complex_|_Population.Old.     0.4908
FALSE MinorAxisLength.1_complex_|_Population.Calf.    0.1014
FALSE MinorAxisLength.1_complex_|_Population.Old.     0.5944
FALSE Perimeter.1_complex_|_Population.Calf.          0.0664
FALSE Perimeter.1_complex_|_Population.Old.           0.5848
FALSE Area.2_complex_|_Population.Calf.               0.0162    *
FALSE Area.2_complex_|_Population.Old.                0.7810
FALSE MajorAxisLength.2_complex_|_Population.Calf.    0.0116    *
FALSE MajorAxisLength.2_complex_|_Population.Old.     0.6960
FALSE MinorAxisLength.2_complex_|_Population.Calf.    0.0086   **
FALSE MinorAxisLength.2_complex_|_Population.Old.     0.6650
FALSE Perimeter.2_complex_|_Population.Calf.          0.0104    *
FALSE Perimeter.2_complex_|_Population.Old.           0.6518
FALSE Area.3_complex_|_Population.Calf.               0.0814
FALSE Area.3_complex_|_Population.Old.                0.9274
FALSE MajorAxisLength.3_complex_|_Population.Calf.    0.1102
FALSE MajorAxisLength.3_complex_|_Population.Old.     0.9380
FALSE MinorAxisLength.3_complex_|_Population.Calf.    0.0996
FALSE MinorAxisLength.3_complex_|_Population.Old.     0.9394
FALSE Perimeter.3_complex_|_Population.Calf.          0.0954
FALSE Perimeter.3_complex_|_Population.Old.           0.9228
FALSE Area.4_complex_|_Population.Calf.               0.2058
FALSE Area.4_complex_|_Population.Old.                0.5260
FALSE MajorAxisLength.4_complex_|_Population.Calf.    0.2162
FALSE MajorAxisLength.4_complex_|_Population.Old.     0.5784
FALSE MinorAxisLength.4_complex_|_Population.Calf.    0.2490
FALSE MinorAxisLength.4_complex_|_Population.Old.     0.5104
FALSE Perimeter.4_complex_|_Population.Calf.          0.1898
FALSE Perimeter.4_complex_|_Population.Old.           0.5262
FALSE Area.5_complex_|_Population.Calf.               0.0126    *
FALSE Area.5_complex_|_Population.Old.                0.5356
FALSE MajorAxisLength.5_complex_|_Population.Calf.    0.0178    *
FALSE MajorAxisLength.5_complex_|_Population.Old.     0.4064
FALSE MinorAxisLength.5_complex_|_Population.Calf.    0.0146    *
FALSE MinorAxisLength.5_complex_|_Population.Old.     0.3694
FALSE Perimeter.5_complex_|_Population.Calf.          0.0204    *
FALSE Perimeter.5_complex_|_Population.Old.           0.5108
FALSE Area.6_complex_|_Population.Calf.               0.0418    *
FALSE Area.6_complex_|_Population.Old.                0.4150
FALSE MajorAxisLength.6_complex_|_Population.Calf.    0.0404    *
FALSE MajorAxisLength.6_complex_|_Population.Old.     0.2698
FALSE MinorAxisLength.6_complex_|_Population.Calf.    0.0518
FALSE MinorAxisLength.6_complex_|_Population.Old.     0.3034
FALSE Perimeter.6_complex_|_Population.Calf.          0.0372    *
FALSE Perimeter.6_complex_|_Population.Old.           0.2982
```

Cobined by aspects

```
FALSE  Call:
FALSE  npc(permTP = res, subsets = ss_asp)
FALSE  permutations.
FALSE
FALSE                 comb.funct nVar  Stat p-value Adjust:maxT sig.
FALSE Area                Fisher   48 111.8  0.0084      0.0084   **
FALSE MajorAxisLength     Fisher   48 124.7  0.0008      0.0032   **
FALSE MinorAxisLength     Fisher   48 125.6  0.0022      0.0032   **
FALSE Perimeter           Fisher   48 122.8  0.0024      0.0036   **
```

Cobined by Shape and Layer

```
FALSE  Call:
FALSE  npc(permTP = res, subsets = ids)
FALSE  permutations.
FALSE
FALSE             comb.funct nVar   Stat p-value Adjust:maxT sig.
FALSE ellipsoid_1     Fisher    8 43.421  0.0006      0.0060   **
FALSE ellipsoid_2     Fisher    8 15.565  0.1052      0.6018
FALSE ellipsoid_3     Fisher    8 14.429  0.1234      0.6204
FALSE ellipsoid_4     Fisher    8  3.030  0.8176      0.8788
FALSE ellipsoid_5     Fisher    8  4.342  0.6864      0.8788
FALSE ellipsoid_6     Fisher    8 10.989  0.2376      0.7082
FALSE round_1         Fisher    8 56.899  0.0002      0.0004  ***
FALSE round_2         Fisher    8 39.093  0.0010      0.0170    *
FALSE round_3         Fisher    8 24.333  0.0228      0.2338
FALSE round_4         Fisher    8 21.576  0.0372      0.3328
FALSE round_5         Fisher    8 31.536  0.0066      0.0786
FALSE round_6         Fisher    8 34.773  0.0036      0.0434    *
FALSE pyramidal_1     Fisher    8 14.749  0.1098      0.6200
FALSE pyramidal_2     Fisher    8 27.723  0.0102      0.1406
FALSE pyramidal_3     Fisher    8  8.514  0.3608      0.7598
FALSE pyramidal_4     Fisher    8 13.781  0.1372      0.6204
FALSE pyramidal_5     Fisher    8 15.378  0.1032      0.6018
FALSE pyramidal_6     Fisher    8 17.530  0.0692      0.4958
FALSE complex_1       Fisher    8 12.726  0.1650      0.6342
FALSE complex_2       Fisher    8 19.347  0.0554      0.4140
FALSE complex_3       Fisher    8  9.652  0.2938      0.7408
FALSE complex_4       Fisher    8  8.669  0.3432      0.7598
FALSE complex_5       Fisher    8 19.714  0.0470      0.4004
FALSE complex_6       Fisher    8 17.228  0.0728      0.5014
```

Cobined by Layer

```
FALSE  Call:
FALSE  npc(permTP = res, subsets = ss_Layers)
FALSE  permutations.
FALSE
FALSE   comb.funct nVar   Stat p-value Adjust:maxT sig.
FALSE 1     Fisher   32 127.79  0.0002      0.0024   **
FALSE 2     Fisher   32 101.73  0.0074      0.0124    *
FALSE 3     Fisher   32  56.93  0.0646      0.0942
FALSE 4     Fisher   32  47.06  0.1150      0.1150
FALSE 5     Fisher   32  70.97  0.0298      0.0462    *
FALSE 6     Fisher   32  80.52  0.0172      0.0324    *
```

Pairwise comparisons

```
FALSE
FALSE         _-_-_-_-_-_-_-_- VARIABLE  Area.1_ellipsoid _-_-_-_-_-_-_-_-
FALSE
FALSE         _-_-_-_-_-_-_-_- Layer  1 _-_-_-_-_-_-_-_-
FALSE                         Raw (upper)
FALSE Adjusted (lower) Adult   Calf   Old
FALSE            Adult    NA -0.691 0.001
FALSE            Calf  0.691     NA 0.002
FALSE            Old   0.002  0.002    NA
FALSE
FALSE         _-_-_-_-_-_-_-_- Layer  2 _-_-_-_-_-_-_-_-
FALSE                         Raw (upper)
FALSE Adjusted (lower) Adult   Calf   Old
FALSE            Adult    NA -0.372 0.365
FALSE            Calf  0.607     NA 0.202
FALSE            Old   0.607  0.607    NA
FALSE
FALSE         _-_-_-_-_-_-_-_- Layer  3 _-_-_-_-_-_-_-_-
FALSE                         Raw (upper)
FALSE Adjusted (lower) Adult  Calf   Old
FALSE            Adult    NA 0.063 0.017
FALSE            Calf  0.063    NA 0.674
FALSE            Old   0.050 0.674    NA
FALSE
FALSE         _-_-_-_-_-_-_-_- Layer  4 _-_-_-_-_-_-_-_-
FALSE                         Raw (upper)
FALSE Adjusted (lower) Adult Calf    Old
FALSE            Adult    NA 0.74 -0.958
FALSE            Calf      1   NA -0.596
FALSE            Old       1 1.00     NA
FALSE
FALSE         _-_-_-_-_-_-_-_- Layer  5 _-_-_-_-_-_-_-_-
FALSE                         Raw (upper)
FALSE Adjusted (lower) Adult  Calf    Old
FALSE            Adult    NA 0.517  0.828
FALSE            Calf      1    NA -0.493
FALSE            Old       1 1.000     NA
FALSE
FALSE         _-_-_-_-_-_-_-_- Layer  6 _-_-_-_-_-_-_-_-
FALSE                         Raw (upper)
FALSE Adjusted (lower) Adult  Calf   Old
FALSE            Adult    NA 0.412 0.117
FALSE            Calf  0.412    NA 0.404
FALSE            Old   0.350 0.404    NA
FALSE
FALSE         _-_-_-_-_-_-_-_- VARIABLE  MajorAxisLength.1_ellipsoid _-_-_-_-_-_-_-_-
FALSE
FALSE         _-_-_-_-_-_-_-_- Layer  1 _-_-_-_-_-_-_-_-
FALSE                         Raw (upper)
FALSE Adjusted (lower) Adult   Calf   Old
FALSE            Adult    NA -0.443 0.001
FALSE            Calf  0.443     NA 0.000
FALSE            Old   0.001  0.001    NA
FALSE
FALSE         _-_-_-_-_-_-_-_- Layer  2 _-_-_-_-_-_-_-_-
FALSE                         Raw (upper)
FALSE Adjusted (lower) Adult   Calf   Old
FALSE            Adult    NA -0.459 0.172
FALSE            Calf  0.459     NA 0.145
FALSE            Old   0.436  0.436    NA
FALSE
FALSE         _-_-_-_-_-_-_-_- Layer  3 _-_-_-_-_-_-_-_-
FALSE                         Raw (upper)
FALSE Adjusted (lower) Adult  Calf   Old
FALSE            Adult    NA 0.022 0.005
FALSE            Calf  0.022    NA 0.687
FALSE            Old   0.014 0.687    NA
FALSE
FALSE         _-_-_-_-_-_-_-_- Layer  4 _-_-_-_-_-_-_-_-
FALSE                         Raw (upper)
FALSE Adjusted (lower) Adult  Calf    Old
FALSE            Adult    NA 0.538  0.842
FALSE            Calf      1    NA -0.638
FALSE            Old       1 1.000     NA
FALSE
FALSE         _-_-_-_-_-_-_-_- Layer  5 _-_-_-_-_-_-_-_-
FALSE                         Raw (upper)
FALSE Adjusted (lower) Adult  Calf    Old
```

```
FALSE           Adult    NA 0.316  0.426
FALSE           Calf  0.947    NA -0.794
FALSE           Old   0.947 0.947     NA
FALSE
FALSE        _-_-_-_-_-_-_-_-_-_- Layer  6 _-_-_-_-_-_-_-_-_-_-
FALSE                         Raw (upper)
FALSE Adjusted (lower) Adult  Calf   Old
FALSE           Adult    NA 0.265 0.059
FALSE           Calf  0.265    NA 0.307
FALSE           Old   0.176 0.307    NA
FALSE
FALSE        _-_-_-_-_-_-_-_-_-_- VARIABLE  MinorAxisLength.1_ellipsoid _-_-_-_-_-_-_-_-_-_-
FALSE
FALSE        _-_-_-_-_-_-_-_-_-_- Layer  1 _-_-_-_-_-_-_-_-_-_-
FALSE                         Raw (upper)
FALSE Adjusted (lower) Adult   Calf   Old
FALSE           Adult    NA -0.464 0.001
FALSE           Calf  0.464     NA 0.002
FALSE           Old   0.002  0.002    NA
FALSE
FALSE        _-_-_-_-_-_-_-_-_-_- Layer  2 _-_-_-_-_-_-_-_-_-_-
FALSE                         Raw (upper)
FALSE Adjusted (lower) Adult   Calf   Old
FALSE           Adult    NA -0.502 0.168
FALSE           Calf  0.502     NA 0.164
FALSE           Old   0.493  0.493    NA
FALSE
FALSE        _-_-_-_-_-_-_-_-_-_- Layer  3 _-_-_-_-_-_-_-_-_-_-
FALSE                         Raw (upper)
FALSE Adjusted (lower) Adult  Calf   Old
FALSE           Adult    NA 0.016 0.003
FALSE           Calf  0.016    NA 0.695
FALSE           Old   0.010 0.695    NA
FALSE
FALSE        _-_-_-_-_-_-_-_-_-_- Layer  4 _-_-_-_-_-_-_-_-_-_-
FALSE                         Raw (upper)
FALSE Adjusted (lower) Adult  Calf    Old
FALSE           Adult    NA 0.529  0.818
FALSE           Calf      1    NA -0.697
FALSE           Old       1 1.000     NA
FALSE
FALSE        _-_-_-_-_-_-_-_-_-_- Layer  5 _-_-_-_-_-_-_-_-_-_-
FALSE                         Raw (upper)
FALSE Adjusted (lower) Adult  Calf    Old
FALSE           Adult    NA 0.265  0.407
FALSE           Calf  0.796    NA -0.719
FALSE           Old   0.796 0.796     NA
FALSE
FALSE        _-_-_-_-_-_-_-_-_-_- Layer  6 _-_-_-_-_-_-_-_-_-_-
FALSE                         Raw (upper)
FALSE Adjusted (lower) Adult  Calf   Old
FALSE           Adult    NA 0.262 0.059
FALSE           Calf  0.262    NA 0.328
FALSE           Old   0.177 0.328    NA
FALSE
FALSE        _-_-_-_-_-_-_-_-_-_- VARIABLE  Perimeter.1_ellipsoid _-_-_-_-_-_-_-_-_-_-
FALSE
FALSE        _-_-_-_-_-_-_-_-_-_- Layer  1 _-_-_-_-_-_-_-_-_-_-
FALSE                         Raw (upper)
FALSE Adjusted (lower) Adult   Calf Old
FALSE           Adult    NA -0.456   0
FALSE           Calf  0.456     NA   0
FALSE           Old   0.001  0.001  NA
FALSE
FALSE        _-_-_-_-_-_-_-_-_-_- Layer  2 _-_-_-_-_-_-_-_-_-_-
FALSE                         Raw (upper)
FALSE Adjusted (lower) Adult   Calf   Old
FALSE           Adult    NA -0.409 0.202
FALSE           Calf  0.527     NA 0.176
FALSE           Old   0.527  0.527    NA
FALSE
FALSE        _-_-_-_-_-_-_-_-_-_- Layer  3 _-_-_-_-_-_-_-_-_-_-
FALSE                         Raw (upper)
FALSE Adjusted (lower) Adult  Calf   Old
FALSE           Adult    NA 0.031 0.007
FALSE           Calf  0.031    NA 0.650
FALSE           Old   0.020 0.650    NA
FALSE
```

```
FALSE         _-_-_-_-_-_-_-_-_-_- Layer  4 _-_-_-_-_-_-_-_-_-_-
FALSE                        Raw (upper)
FALSE Adjusted (lower) Adult  Calf    Old
FALSE            Adult    NA 0.522  0.834
FALSE            Calf      1    NA -0.586
FALSE            Old       1 1.000     NA
FALSE
FALSE         _-_-_-_-_-_-_-_-_-_- Layer  5 _-_-_-_-_-_-_-_-_-_-
FALSE                        Raw (upper)
FALSE Adjusted (lower) Adult  Calf    Old
FALSE            Adult    NA 0.402  0.453
FALSE            Calf      1    NA -0.911
FALSE            Old       1 1.000     NA
FALSE
FALSE         _-_-_-_-_-_-_-_-_-_- Layer  6 _-_-_-_-_-_-_-_-_-_-
FALSE                        Raw (upper)
FALSE Adjusted (lower) Adult  Calf   Old
FALSE            Adult    NA 0.352 0.072
FALSE            Calf  0.352    NA 0.256
FALSE            Old   0.217 0.256    NA
FALSE
FALSE         _-_-_-_-_-_-_-_-_-_- VARIABLE  Area.1_round _-_-_-_-_-_-_-_-_-_-
FALSE
FALSE         _-_-_-_-_-_-_-_-_-_- Layer  1 _-_-_-_-_-_-_-_-_-_-
FALSE                        Raw (upper)
FALSE Adjusted (lower) Adult   Calf   Old
FALSE            Adult    NA -0.004 0.003
FALSE            Calf  0.008     NA 0.003
FALSE            Old   0.008  0.008    NA
FALSE
FALSE         _-_-_-_-_-_-_-_-_-_- Layer  2 _-_-_-_-_-_-_-_-_-_-
FALSE                        Raw (upper)
FALSE Adjusted (lower) Adult   Calf   Old
FALSE            Adult    NA -0.028 0.202
FALSE            Calf  0.050     NA 0.017
FALSE            Old   0.202  0.050    NA
FALSE
FALSE         _-_-_-_-_-_-_-_-_-_- Layer  3 _-_-_-_-_-_-_-_-_-_-
FALSE                        Raw (upper)
FALSE Adjusted (lower) Adult   Calf   Old
FALSE            Adult    NA -0.770 0.049
FALSE            Calf  0.770     NA 0.037
FALSE            Old   0.112  0.112    NA
FALSE
FALSE         _-_-_-_-_-_-_-_-_-_- Layer  4 _-_-_-_-_-_-_-_-_-_-
FALSE                        Raw (upper)
FALSE Adjusted (lower) Adult   Calf   Old
FALSE            Adult    NA -0.462 0.126
FALSE            Calf  0.462     NA 0.096
FALSE            Old   0.287  0.287    NA
FALSE
FALSE         _-_-_-_-_-_-_-_-_-_- Layer  5 _-_-_-_-_-_-_-_-_-_-
FALSE                        Raw (upper)
FALSE Adjusted (lower) Adult   Calf   Old
FALSE            Adult    NA -0.820 0.097
FALSE            Calf  0.820     NA 0.015
FALSE            Old   0.097  0.046    NA
FALSE
FALSE         _-_-_-_-_-_-_-_-_-_- Layer  6 _-_-_-_-_-_-_-_-_-_-
FALSE                        Raw (upper)
FALSE Adjusted (lower) Adult   Calf   Old
FALSE            Adult    NA -0.882 0.051
FALSE            Calf  0.882     NA 0.005
FALSE            Old   0.051  0.016    NA
FALSE
FALSE         _-_-_-_-_-_-_-_-_-_- VARIABLE  MajorAxisLength.1_round _-_-_-_-_-_-_-_-_-_-
FALSE
FALSE         _-_-_-_-_-_-_-_-_-_- Layer  1 _-_-_-_-_-_-_-_-_-_-
FALSE                        Raw (upper)
FALSE Adjusted (lower) Adult   Calf   Old
FALSE            Adult    NA -0.003 0.005
FALSE            Calf  0.006     NA 0.002
FALSE            Old   0.006  0.006    NA
FALSE
FALSE         _-_-_-_-_-_-_-_-_-_- Layer  2 _-_-_-_-_-_-_-_-_-_-
FALSE                        Raw (upper)
FALSE Adjusted (lower) Adult   Calf   Old
FALSE            Adult    NA -0.024 0.048
```

```
FALSE            Calf  0.026     NA 0.009
FALSE            Old   0.048  0.026    NA
FALSE
FALSE         _-_-_-_-_-_-_-_-_- Layer  3 _-_-_-_-_-_-_-_-_-
FALSE                        Raw (upper)
FALSE Adjusted (lower) Adult   Calf   Old
FALSE            Adult    NA -0.770 0.011
FALSE            Calf  0.770     NA 0.029
FALSE            Old   0.034  0.034    NA
FALSE
FALSE         _-_-_-_-_-_-_-_-_- Layer  4 _-_-_-_-_-_-_-_-_-
FALSE                        Raw (upper)
FALSE Adjusted (lower) Adult   Calf   Old
FALSE            Adult    NA -0.425 0.041
FALSE            Calf  0.425     NA 0.071
FALSE            Old   0.122  0.122    NA
FALSE
FALSE         _-_-_-_-_-_-_-_-_- Layer  5 _-_-_-_-_-_-_-_-_-
FALSE                        Raw (upper)
FALSE Adjusted (lower) Adult   Calf   Old
FALSE            Adult    NA -0.837 0.008
FALSE            Calf  0.837     NA 0.006
FALSE            Old   0.017  0.017    NA
FALSE
FALSE         _-_-_-_-_-_-_-_-_- Layer  6 _-_-_-_-_-_-_-_-_-
FALSE                        Raw (upper)
FALSE Adjusted (lower) Adult  Calf   Old
FALSE            Adult    NA -0.73 0.007
FALSE            Calf   0.73    NA 0.003
FALSE            Old    0.01  0.01    NA
FALSE
FALSE         _-_-_-_-_-_-_-_-_- VARIABLE  MinorAxisLength.1_round _-_-_-_-_-_-_-_-_-
FALSE
FALSE         _-_-_-_-_-_-_-_-_- Layer  1 _-_-_-_-_-_-_-_-_-
FALSE                        Raw (upper)
FALSE Adjusted (lower) Adult   Calf   Old
FALSE            Adult    NA -0.006 0.004
FALSE            Calf  0.008     NA 0.003
FALSE            Old   0.008  0.008    NA
FALSE
FALSE         _-_-_-_-_-_-_-_-_- Layer  2 _-_-_-_-_-_-_-_-_-
FALSE                        Raw (upper)
FALSE Adjusted (lower) Adult   Calf   Old
FALSE            Adult    NA -0.023 0.068
FALSE            Calf  0.023     NA 0.007
FALSE            Old   0.068  0.020    NA
FALSE
FALSE         _-_-_-_-_-_-_-_-_- Layer  3 _-_-_-_-_-_-_-_-_-
FALSE                        Raw (upper)
FALSE Adjusted (lower) Adult   Calf   Old
FALSE            Adult    NA -0.736 0.019
FALSE            Calf  0.736     NA 0.029
FALSE            Old   0.056  0.056    NA
FALSE
FALSE         _-_-_-_-_-_-_-_-_- Layer  4 _-_-_-_-_-_-_-_-_-
FALSE                        Raw (upper)
FALSE Adjusted (lower) Adult   Calf   Old
FALSE            Adult    NA -0.546 0.041
FALSE            Calf  0.546     NA 0.096
FALSE            Old   0.123  0.123    NA
FALSE
FALSE         _-_-_-_-_-_-_-_-_- Layer  5 _-_-_-_-_-_-_-_-_-
FALSE                        Raw (upper)
FALSE Adjusted (lower) Adult Calf   Old
FALSE            Adult    NA 0.00 0.011
FALSE            Calf  0.000   NA 0.010
FALSE            Old   0.011 0.01    NA
FALSE
FALSE         _-_-_-_-_-_-_-_-_- Layer  6 _-_-_-_-_-_-_-_-_-
FALSE                        Raw (upper)
FALSE Adjusted (lower) Adult   Calf   Old
FALSE            Adult    NA -0.739 0.013
FALSE            Calf  0.739     NA 0.002
FALSE            Old   0.013  0.007    NA
FALSE
FALSE         _-_-_-_-_-_-_-_-_- VARIABLE  Perimeter.1_round _-_-_-_-_-_-_-_-_-
FALSE
FALSE         _-_-_-_-_-_-_-_-_- Layer  1 _-_-_-_-_-_-_-_-_-
```

```
FALSE                         Raw (upper)
FALSE Adjusted (lower) Adult   Calf   Old
FALSE            Adult    NA -0.003 0.004
FALSE            Calf  0.007     NA 0.002
FALSE            Old   0.007  0.007    NA
FALSE
FALSE         _-_-_-_-_-_-_-_-_- Layer  2 _-_-_-_-_-_-_-_-_-
FALSE                         Raw (upper)
FALSE Adjusted (lower) Adult   Calf   Old
FALSE            Adult    NA -0.024 0.070
FALSE            Calf  0.024     NA 0.008
FALSE            Old   0.070  0.023    NA
FALSE
FALSE         _-_-_-_-_-_-_-_-_- Layer  3 _-_-_-_-_-_-_-_-_-
FALSE                         Raw (upper)
FALSE Adjusted (lower) Adult   Calf   Old
FALSE            Adult    NA -0.748 0.024
FALSE            Calf  0.748     NA 0.028
FALSE            Old   0.072  0.072    NA
FALSE
FALSE         _-_-_-_-_-_-_-_-_- Layer  4 _-_-_-_-_-_-_-_-_-
FALSE                         Raw (upper)
FALSE Adjusted (lower) Adult   Calf   Old
FALSE            Adult    NA -0.441 0.038
FALSE            Calf  0.441     NA 0.066
FALSE            Old   0.113  0.113    NA
FALSE
FALSE         _-_-_-_-_-_-_-_-_- Layer  5 _-_-_-_-_-_-_-_-_-
FALSE                         Raw (upper)
FALSE Adjusted (lower) Adult   Calf   Old
FALSE            Adult    NA -0.760 0.012
FALSE            Calf  0.760     NA 0.002
FALSE            Old   0.012  0.007    NA
FALSE
FALSE         _-_-_-_-_-_-_-_-_- Layer  6 _-_-_-_-_-_-_-_-_-
FALSE                         Raw (upper)
FALSE Adjusted (lower) Adult   Calf   Old
FALSE            Adult    NA -0.703 0.009
FALSE            Calf  0.703     NA 0.000
FALSE            Old   0.009  0.001    NA
FALSE
FALSE         _-_-_-_-_-_-_-_-_- VARIABLE  Area.1_pyramidal _-_-_-_-_-_-_-_-_-
FALSE
FALSE         _-_-_-_-_-_-_-_-_- Layer  1 _-_-_-_-_-_-_-_-_-
FALSE                         Raw (upper)
FALSE Adjusted (lower) Adult  Calf   Old
FALSE            Adult    NA 0.400 0.011
FALSE            Calf  0.400    NA 0.293
FALSE            Old   0.034 0.293    NA
FALSE
FALSE         _-_-_-_-_-_-_-_-_- Layer  2 _-_-_-_-_-_-_-_-_-
FALSE                         Raw (upper)
FALSE Adjusted (lower) Adult   Calf   Old
FALSE            Adult    NA -0.030 0.677
FALSE            Calf  0.091     NA 0.036
FALSE            Old   0.677  0.091    NA
FALSE
FALSE         _-_-_-_-_-_-_-_-_- Layer  3 _-_-_-_-_-_-_-_-_-
FALSE                         Raw (upper)
FALSE Adjusted (lower) Adult   Calf   Old
FALSE            Adult    NA -0.989 0.305
FALSE            Calf  0.989     NA 0.354
FALSE            Old   0.914  0.914    NA
FALSE
FALSE         _-_-_-_-_-_-_-_-_- Layer  4 _-_-_-_-_-_-_-_-_-
FALSE                         Raw (upper)
FALSE Adjusted (lower) Adult   Calf   Old
FALSE            Adult    NA -0.146 0.735
FALSE            Calf  0.195     NA 0.065
FALSE            Old   0.735  0.195    NA
FALSE
FALSE         _-_-_-_-_-_-_-_-_- Layer  5 _-_-_-_-_-_-_-_-_-
FALSE                         Raw (upper)
FALSE Adjusted (lower) Adult   Calf    Old
FALSE            Adult    NA -0.037 -0.576
FALSE            Calf  0.110     NA  0.120
FALSE            Old   0.576  0.120     NA
FALSE
```

```
FALSE         _-_-_-_-_-_-_-_-_-_- Layer  6 _-_-_-_-_-_-_-_-_-_-
FALSE                         Raw (upper)
FALSE Adjusted (lower) Adult   Calf   Old
FALSE            Adult    NA -0.064 0.663
FALSE            Calf  0.192     NA 0.113
FALSE            Old   0.663  0.192    NA
FALSE
FALSE         _-_-_-_-_-_-_-_-_-_- VARIABLE  MajorAxisLength.1_pyramidal _-_-_-_-_-_-_-_-_-_-
FALSE
FALSE         _-_-_-_-_-_-_-_-_-_- Layer  1 _-_-_-_-_-_-_-_-_-_-
FALSE                         Raw (upper)
FALSE Adjusted (lower) Adult  Calf   Old
FALSE            Adult    NA 0.361 0.118
FALSE            Calf  0.361    NA 0.796
FALSE            Old   0.355 0.796    NA
FALSE
FALSE         _-_-_-_-_-_-_-_-_-_- Layer  2 _-_-_-_-_-_-_-_-_-_-
FALSE                         Raw (upper)
FALSE Adjusted (lower) Adult   Calf   Old
FALSE            Adult    NA -0.041 0.745
FALSE            Calf  0.114     NA 0.038
FALSE            Old   0.745  0.114    NA
FALSE
FALSE         _-_-_-_-_-_-_-_-_-_- Layer  3 _-_-_-_-_-_-_-_-_-_-
FALSE                         Raw (upper)
FALSE Adjusted (lower) Adult   Calf  Old
FALSE            Adult    NA -0.772 0.39
FALSE            Calf      1     NA 0.37
FALSE            Old       1  1.000   NA
FALSE
FALSE         _-_-_-_-_-_-_-_-_-_- Layer  4 _-_-_-_-_-_-_-_-_-_-
FALSE                         Raw (upper)
FALSE Adjusted (lower) Adult   Calf   Old
FALSE            Adult    NA -0.244 0.693
FALSE            Calf  0.421     NA 0.140
FALSE            Old   0.693  0.421    NA
FALSE
FALSE         _-_-_-_-_-_-_-_-_-_- Layer  5 _-_-_-_-_-_-_-_-_-_-
FALSE                         Raw (upper)
FALSE Adjusted (lower) Adult   Calf    Old
FALSE            Adult    NA -0.016 -0.670
FALSE            Calf  0.049     NA  0.139
FALSE            Old   0.670  0.139     NA
FALSE
FALSE         _-_-_-_-_-_-_-_-_-_- Layer  6 _-_-_-_-_-_-_-_-_-_-
FALSE                         Raw (upper)
FALSE Adjusted (lower) Adult   Calf   Old
FALSE            Adult    NA -0.027 0.952
FALSE            Calf  0.080     NA 0.128
FALSE            Old   0.952  0.128    NA
FALSE
FALSE         _-_-_-_-_-_-_-_-_-_- VARIABLE  MinorAxisLength.1_pyramidal _-_-_-_-_-_-_-_-_-_-
FALSE
FALSE         _-_-_-_-_-_-_-_-_-_- Layer  1 _-_-_-_-_-_-_-_-_-_-
FALSE                         Raw (upper)
FALSE Adjusted (lower) Adult  Calf   Old
FALSE            Adult    NA 0.525 0.000
FALSE            Calf  0.525    NA 0.061
FALSE            Old   0.001 0.061    NA
FALSE
FALSE         _-_-_-_-_-_-_-_-_-_- Layer  2 _-_-_-_-_-_-_-_-_-_-
FALSE                         Raw (upper)
FALSE Adjusted (lower) Adult   Calf   Old
FALSE            Adult    NA -0.032 0.366
FALSE            Calf  0.060     NA 0.020
FALSE            Old   0.366  0.060    NA
FALSE
FALSE         _-_-_-_-_-_-_-_-_-_- Layer  3 _-_-_-_-_-_-_-_-_-_-
FALSE                         Raw (upper)
FALSE Adjusted (lower) Adult  Calf   Old
FALSE            Adult    NA 0.717 0.109
FALSE            Calf  0.717    NA 0.317
FALSE            Old   0.327 0.327    NA
FALSE
FALSE         _-_-_-_-_-_-_-_-_-_- Layer  4 _-_-_-_-_-_-_-_-_-_-
FALSE                         Raw (upper)
FALSE Adjusted (lower) Adult   Calf   Old
FALSE            Adult    NA -0.327 0.613
```

```
FALSE            Calf  0.327     NA 0.101
FALSE            Old   0.613  0.302    NA
FALSE
FALSE         _-_-_-_-_-_-_-_-_- Layer  5 _-_-_-_-_-_-_-_-_-
FALSE                        Raw (upper)
FALSE Adjusted (lower) Adult   Calf   Old
FALSE            Adult    NA -0.185 0.864
FALSE            Calf  0.514     NA 0.171
FALSE            Old   0.864  0.514    NA
FALSE
FALSE         _-_-_-_-_-_-_-_-_- Layer  6 _-_-_-_-_-_-_-_-_-
FALSE                        Raw (upper)
FALSE Adjusted (lower) Adult   Calf   Old
FALSE            Adult    NA -0.447 0.263
FALSE            Calf  0.457     NA 0.152
FALSE            Old   0.457  0.457    NA
FALSE
FALSE         _-_-_-_-_-_-_-_-_- VARIABLE  Perimeter.1_pyramidal _-_-_-_-_-_-_-_-_-
FALSE
FALSE         _-_-_-_-_-_-_-_-_- Layer  1 _-_-_-_-_-_-_-_-_-
FALSE                        Raw (upper)
FALSE Adjusted (lower) Adult  Calf   Old
FALSE            Adult    NA 0.426 0.035
FALSE            Calf  0.426    NA 0.469
FALSE            Old   0.104 0.469    NA
FALSE
FALSE         _-_-_-_-_-_-_-_-_- Layer  2 _-_-_-_-_-_-_-_-_-
FALSE                        Raw (upper)
FALSE Adjusted (lower) Adult   Calf   Old
FALSE            Adult    NA -0.025 0.737
FALSE            Calf  0.074     NA 0.026
FALSE            Old   0.737  0.074    NA
FALSE
FALSE         _-_-_-_-_-_-_-_-_- Layer  3 _-_-_-_-_-_-_-_-_-
FALSE                        Raw (upper)
FALSE Adjusted (lower) Adult   Calf   Old
FALSE            Adult    NA -0.668 0.430
FALSE            Calf  0.897     NA 0.299
FALSE            Old   0.897  0.897    NA
FALSE
FALSE         _-_-_-_-_-_-_-_-_- Layer  4 _-_-_-_-_-_-_-_-_-
FALSE                        Raw (upper)
FALSE Adjusted (lower) Adult   Calf   Old
FALSE            Adult    NA -0.252 0.621
FALSE            Calf  0.297     NA 0.099
FALSE            Old   0.621  0.297    NA
FALSE
FALSE         _-_-_-_-_-_-_-_-_- Layer  5 _-_-_-_-_-_-_-_-_-
FALSE                        Raw (upper)
FALSE Adjusted (lower) Adult   Calf    Old
FALSE            Adult    NA -0.037 -0.678
FALSE            Calf  0.110     NA  0.161
FALSE            Old   0.678  0.161     NA
FALSE
FALSE         _-_-_-_-_-_-_-_-_- Layer  6 _-_-_-_-_-_-_-_-_-
FALSE                        Raw (upper)
FALSE Adjusted (lower) Adult   Calf   Old
FALSE            Adult    NA -0.037 0.685
FALSE            Calf  0.110     NA 0.119
FALSE            Old   0.685  0.119    NA
FALSE
FALSE         _-_-_-_-_-_-_-_-_- VARIABLE  Area.1_complex _-_-_-_-_-_-_-_-_-
FALSE
FALSE         _-_-_-_-_-_-_-_-_- Layer  1 _-_-_-_-_-_-_-_-_-
FALSE                        Raw (upper)
FALSE Adjusted (lower) Adult   Calf    Old
FALSE            Adult    NA -0.141 -0.444
FALSE            Calf  0.422     NA  0.349
FALSE            Old   0.444  0.422     NA
FALSE
FALSE         _-_-_-_-_-_-_-_-_- Layer  2 _-_-_-_-_-_-_-_-_-
FALSE                        Raw (upper)
FALSE Adjusted (lower) Adult   Calf    Old
FALSE            Adult    NA -0.024 -0.305
FALSE            Calf  0.073     NA  0.074
FALSE            Old   0.305  0.074     NA
FALSE
FALSE         _-_-_-_-_-_-_-_-_- Layer  3 _-_-_-_-_-_-_-_-_-
```

```
FALSE                        Raw (upper)
FALSE Adjusted (lower) Adult   Calf    Old
FALSE            Adult    NA -0.096 -0.482
FALSE            Calf  0.289     NA  0.119
FALSE            Old   0.482  0.289     NA
FALSE
FALSE         _-_-_-_-_-_-_-_-_- Layer  4 _-_-_-_-_-_-_-_-_-
FALSE                        Raw (upper)
FALSE Adjusted (lower) Adult   Calf   Old
FALSE            Adult    NA -0.237 0.913
FALSE            Calf  0.710     NA 0.263
FALSE            Old   0.913  0.710    NA
FALSE
FALSE         _-_-_-_-_-_-_-_-_- Layer  5 _-_-_-_-_-_-_-_-_-
FALSE                        Raw (upper)
FALSE Adjusted (lower) Adult   Calf    Old
FALSE            Adult    NA -0.020 -0.679
FALSE            Calf  0.028     NA  0.009
FALSE            Old   0.679  0.028     NA
FALSE
FALSE         _-_-_-_-_-_-_-_-_- Layer  6 _-_-_-_-_-_-_-_-_-
FALSE                        Raw (upper)
FALSE Adjusted (lower) Adult   Calf    Old
FALSE            Adult    NA -0.102 -0.956
FALSE            Calf  0.191     NA  0.064
FALSE            Old   0.956  0.191     NA
FALSE
FALSE         _-_-_-_-_-_-_-_-_- VARIABLE  MajorAxisLength.1_complex _-_-_-_-_-_-_-_-_-
FALSE
FALSE         _-_-_-_-_-_-_-_-_- Layer  1 _-_-_-_-_-_-_-_-_-
FALSE                        Raw (upper)
FALSE Adjusted (lower) Adult   Calf    Old
FALSE            Adult    NA -0.051 -0.725
FALSE            Calf  0.154     NA  0.147
FALSE            Old   0.725  0.154     NA
FALSE
FALSE         _-_-_-_-_-_-_-_-_- Layer  2 _-_-_-_-_-_-_-_-_-
FALSE                        Raw (upper)
FALSE Adjusted (lower) Adult   Calf    Old
FALSE            Adult    NA -0.025 -0.349
FALSE            Calf  0.076     NA  0.070
FALSE            Old   0.349  0.076     NA
FALSE
FALSE         _-_-_-_-_-_-_-_-_- Layer  3 _-_-_-_-_-_-_-_-_-
FALSE                        Raw (upper)
FALSE Adjusted (lower) Adult   Calf    Old
FALSE            Adult    NA -0.116 -0.497
FALSE            Calf  0.347     NA  0.191
FALSE            Old   0.497  0.347     NA
FALSE
FALSE         _-_-_-_-_-_-_-_-_- Layer  4 _-_-_-_-_-_-_-_-_-
FALSE                        Raw (upper)
FALSE Adjusted (lower) Adult   Calf   Old
FALSE            Adult    NA -0.258 0.982
FALSE            Calf  0.773     NA 0.305
FALSE            Old   0.982  0.773    NA
FALSE
FALSE         _-_-_-_-_-_-_-_-_- Layer  5 _-_-_-_-_-_-_-_-_-
FALSE                        Raw (upper)
FALSE Adjusted (lower) Adult   Calf    Old
FALSE            Adult    NA -0.031 -0.890
FALSE            Calf  0.052     NA  0.017
FALSE            Old   0.890  0.052     NA
FALSE
FALSE         _-_-_-_-_-_-_-_-_- Layer  6 _-_-_-_-_-_-_-_-_-
FALSE                        Raw (upper)
FALSE Adjusted (lower) Adult   Calf   Old
FALSE            Adult    NA -0.136 0.750
FALSE            Calf  0.172     NA 0.057
FALSE            Old   0.750  0.172    NA
FALSE
FALSE         _-_-_-_-_-_-_-_-_- VARIABLE  MinorAxisLength.1_complex _-_-_-_-_-_-_-_-_-
FALSE
FALSE         _-_-_-_-_-_-_-_-_- Layer  1 _-_-_-_-_-_-_-_-_-
FALSE                        Raw (upper)
FALSE Adjusted (lower) Adult   Calf    Old
FALSE            Adult    NA -0.193 -0.792
FALSE            Calf  0.580     NA  0.274
```

```
FALSE           Old   0.792  0.580     NA
FALSE
FALSE        _-_-_-_-_-_-_-_-_- Layer  2 _-_-_-_-_-_-_-_-_-
FALSE                        Raw (upper)
FALSE Adjusted (lower) Adult   Calf    Old
FALSE           Adult    NA -0.027 -0.376
FALSE           Calf  0.082     NA  0.058
FALSE           Old   0.376  0.082     NA
FALSE
FALSE        _-_-_-_-_-_-_-_-_- Layer  3 _-_-_-_-_-_-_-_-_-
FALSE                        Raw (upper)
FALSE Adjusted (lower) Adult   Calf    Old
FALSE           Adult    NA -0.099 -0.470
FALSE           Calf  0.297     NA  0.179
FALSE           Old   0.470  0.297     NA
FALSE
FALSE        _-_-_-_-_-_-_-_-_- Layer  4 _-_-_-_-_-_-_-_-_-
FALSE                        Raw (upper)
FALSE Adjusted (lower) Adult   Calf   Old
FALSE           Adult    NA -0.279 0.867
FALSE           Calf  0.838     NA 0.322
FALSE           Old   0.867  0.838    NA
FALSE
FALSE        _-_-_-_-_-_-_-_-_- Layer  5 _-_-_-_-_-_-_-_-_-
FALSE                        Raw (upper)
FALSE Adjusted (lower) Adult   Calf    Old
FALSE           Adult    NA -0.024 -0.880
FALSE           Calf  0.056     NA  0.019
FALSE           Old   0.880  0.056     NA
FALSE
FALSE        _-_-_-_-_-_-_-_-_- Layer  6 _-_-_-_-_-_-_-_-_-
FALSE                        Raw (upper)
FALSE Adjusted (lower) Adult   Calf   Old
FALSE           Adult    NA -0.155 0.763
FALSE           Calf  0.236     NA 0.079
FALSE           Old   0.763  0.236    NA
FALSE
FALSE        _-_-_-_-_-_-_-_-_- VARIABLE  Perimeter.1_complex _-_-_-_-_-_-_-_-_-
FALSE
FALSE        _-_-_-_-_-_-_-_-_- Layer  1 _-_-_-_-_-_-_-_-_-
FALSE                        Raw (upper)
FALSE Adjusted (lower) Adult   Calf    Old
FALSE           Adult    NA -0.132 -0.736
FALSE           Calf  0.395     NA  0.216
FALSE           Old   0.736  0.395     NA
FALSE
FALSE        _-_-_-_-_-_-_-_-_- Layer  2 _-_-_-_-_-_-_-_-_-
FALSE                        Raw (upper)
FALSE Adjusted (lower) Adult   Calf    Old
FALSE           Adult    NA -0.018 -0.396
FALSE           Calf  0.053     NA  0.067
FALSE           Old   0.396  0.067     NA
FALSE
FALSE        _-_-_-_-_-_-_-_-_- Layer  3 _-_-_-_-_-_-_-_-_-
FALSE                        Raw (upper)
FALSE Adjusted (lower) Adult   Calf    Old
FALSE           Adult    NA -0.089 -0.353
FALSE           Calf  0.266     NA  0.212
FALSE           Old   0.353  0.266     NA
FALSE
FALSE        _-_-_-_-_-_-_-_-_- Layer  4 _-_-_-_-_-_-_-_-_-
FALSE                        Raw (upper)
FALSE Adjusted (lower) Adult   Calf   Old
FALSE           Adult    NA -0.223 0.954
FALSE           Calf  0.668     NA 0.259
FALSE           Old   0.954  0.668    NA
FALSE
FALSE        _-_-_-_-_-_-_-_-_- Layer  5 _-_-_-_-_-_-_-_-_-
FALSE                        Raw (upper)
FALSE Adjusted (lower) Adult   Calf    Old
FALSE           Adult    NA -0.032 -0.747
FALSE           Calf  0.097     NA  0.034
FALSE           Old   0.747  0.097     NA
FALSE
FALSE        _-_-_-_-_-_-_-_-_- Layer  6 _-_-_-_-_-_-_-_-_-
FALSE                        Raw (upper)
FALSE Adjusted (lower) Adult   Calf   Old
FALSE           Adult    NA -0.114 0.829
```

FALSE	Calf	0.188	NA	0.063
FALSE	Old	0.829	0.188	NA
