# Supplementary material for: Age-related changes in the primary auditory cortex of newborn, adults and aging bottlenose dolphins (Tursiops truncatus) are located in the upper cortical layers
Source: Front Neuroanat. 2024 Jan 5;17:1330384. doi: 10.3389/fnana.2023.1330384 (PMC10796513; doi:10.3389/fnana.2023.1330384)
Supplement: Supplementary file 2 [file Data_Sheet_2.pdf]

Analysis Shape

- Descriprives
  - means by subject
    - Eccentricity
    - Solidity
    - Extent
    - InvAR
    - ConvexCircularity
  - Radar Plot
  - Density
  - Principal Components
    - Layer 1
    - Layer 2
    - Layer 3
    - Layer 4
    - Layer 5
    - Layer 6
- Inferential Analysis
  - Univariate
  - Cobined by aspects
  - Cobined by Shape and Layer
  - Cobined by Layer
  - Pairwise comparisons

Descriprives

means by subject

Eccentricity

Population	Shape	Layer_ID	mean	sd
Adult	ellipsoid	1	0.78	0.00
Calf	ellipsoid	1	0.78	0.00
Old	ellipsoid	1	0.78	0.00
Adult	ellipsoid	2	0.78	0.00
Calf	ellipsoid	2	0.78	0.00
Old	ellipsoid	2	0.78	0.00
Adult	ellipsoid	3	0.78	0.00
Calf	ellipsoid	3	0.78	0.00
Old	ellipsoid	3	0.78	0.00
Adult	ellipsoid	4	0.79	0.01
Calf	ellipsoid	4	0.78	0.01
Old	ellipsoid	4	0.78	0.00
Adult	ellipsoid	5	0.78	0.00
Calf	ellipsoid	5	0.78	0.00
Old	ellipsoid	5	0.78	0.00
Adult	ellipsoid	6	0.78	0.00
Calf	ellipsoid	6	0.79	0.00
Old	ellipsoid	6	0.79	0.00
Adult	round	1	0.58	0.01
Calf	round	1	0.55	0.03
Old	round	1	0.59	0.01
Adult	round	2	0.59	0.01

Population	Shape	Layer_ID	mean	sd
Calf	round	2	0.58	0.01
Old	round	2	0.60	0.01
Adult	round	3	0.59	0.01
Calf	round	3	0.58	0.01
Old	round	3	0.60	0.01
Adult	round	4	0.59	0.02
Calf	round	4	0.58	0.02
Old	round	4	0.59	0.01
Adult	round	5	0.58	0.01
Calf	round	5	0.57	0.02
Old	round	5	0.59	0.01
Adult	round	6	0.58	0.02
Calf	round	6	0.57	0.02
Old	round	6	0.59	0.01
Adult	pyramidal	1	0.87	0.01
Calf	pyramidal	1	0.88	0.01
Old	pyramidal	1	0.86	0.01
Adult	pyramidal	2	0.87	0.01
Calf	pyramidal	2	0.87	0.01
Old	pyramidal	2	0.87	0.01
Adult	pyramidal	3	0.87	0.01
Calf	pyramidal	3	0.86	0.00
Old	pyramidal	3	0.86	0.01
Adult	pyramidal	4	0.87	0.01
Calf	pyramidal	4	0.86	0.01
Old	pyramidal	4	0.86	0.01
Adult	pyramidal	5	0.87	0.01
Calf	pyramidal	5	0.86	0.01
Old	pyramidal	5	0.86	0.01
Adult	pyramidal	6	0.87	0.01
Calf	pyramidal	6	0.87	0.00
Old	pyramidal	6	0.86	0.01
Adult	complex	1	0.66	0.01
Calf	complex	1	0.65	0.02
Old	complex	1	0.66	0.03
Adult	complex	2	0.66	0.01
Calf	complex	2	0.67	0.01
Old	complex	2	0.66	0.01
Adult	complex	3	0.65	0.01
Calf	complex	3	0.66	0.01
Old	complex	3	0.65	0.00
Adult	complex	4	0.67	0.02
Calf	complex	4	0.66	0.03
Old	complex	4	0.66	0.03
Adult	complex	5	0.65	0.01
Calf	complex	5	0.66	0.01

Population	Shape	Layer_ID	mean	sd
Old	complex	5	0.65	0.02
Adult	complex	6	0.66	0.01
Calf	complex	6	0.65	0.01
Old	complex	6	0.66	0.01

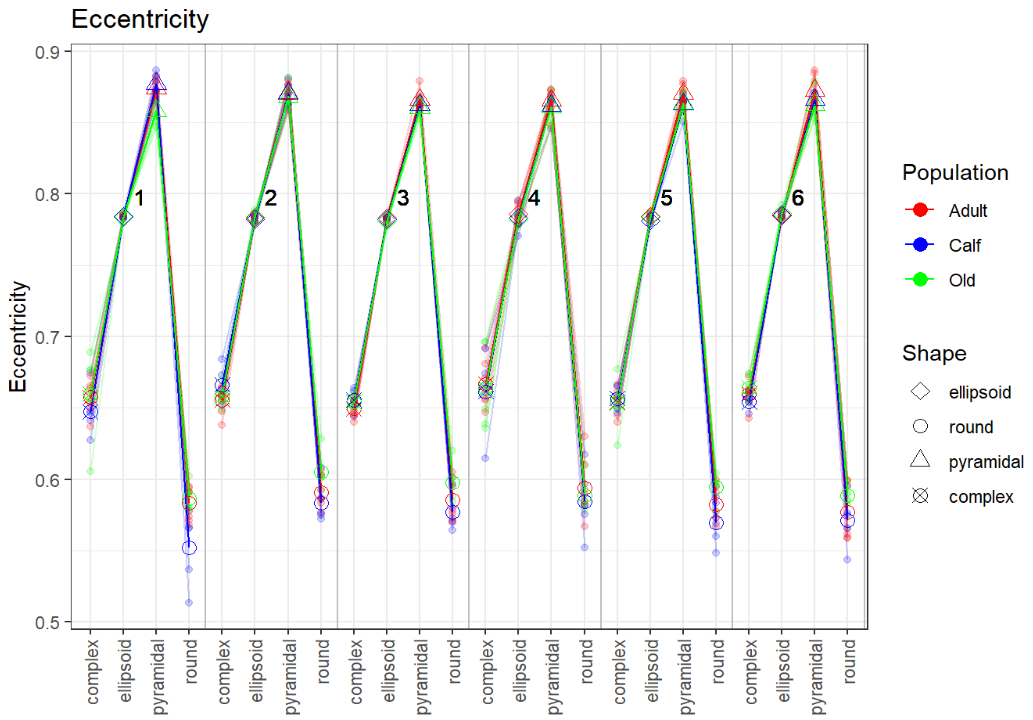

Solidity

Population	Shape	Layer_ID	mean	sd
Adult	ellipsoid	1	0.95	0.00
Calf	ellipsoid	1	0.96	0.01
Old	ellipsoid	1	0.95	0.01
Adult	ellipsoid	2	0.94	0.01
Calf	ellipsoid	2	0.95	0.01
Old	ellipsoid	2	0.94	0.01
Adult	ellipsoid	3	0.95	0.01
Calf	ellipsoid	3	0.95	0.01
Old	ellipsoid	3	0.94	0.01
Adult	ellipsoid	4	0.94	0.02
Calf	ellipsoid	4	0.94	0.01
Old	ellipsoid	4	0.94	0.01
Adult	ellipsoid	5	0.94	0.01
Calf	ellipsoid	5	0.95	0.01
Old	ellipsoid	5	0.94	0.01
Adult	ellipsoid	6	0.95	0.01
Calf	ellipsoid	6	0.95	0.01
Old	ellipsoid	6	0.94	0.01
Adult	round	1	0.96	0.00
Calf	round	1	0.97	0.00
Old	round	1	0.96	0.00
Adult	round	2	0.95	0.00
Calf	round	2	0.96	0.01

Population	Shape	Layer_ID	mean	sd
Old	round	2	0.95	0.01
Adult	round	3	0.95	0.00
Calf	round	3	0.96	0.01
Old	round	3	0.95	0.01
Adult	round	4	0.96	0.01
Calf	round	4	0.96	0.01
Old	round	4	0.95	0.01
Adult	round	5	0.96	0.00
Calf	round	5	0.96	0.00
Old	round	5	0.95	0.00
Adult	round	6	0.96	0.00
Calf	round	6	0.96	0.00
Old	round	6	0.95	0.00
Adult	pyramidal	1	0.90	0.01
Calf	pyramidal	1	0.91	0.01
Old	pyramidal	1	0.90	0.01
Adult	pyramidal	2	0.87	0.01
Calf	pyramidal	2	0.90	0.01
Old	pyramidal	2	0.87	0.01
Adult	pyramidal	3	0.88	0.01
Calf	pyramidal	3	0.90	0.01
Old	pyramidal	3	0.88	0.01
Adult	pyramidal	4	0.88	0.02
Calf	pyramidal	4	0.88	0.02
Old	pyramidal	4	0.88	0.01
Adult	pyramidal	5	0.87	0.01
Calf	pyramidal	5	0.89	0.02
Old	pyramidal	5	0.88	0.01
Adult	pyramidal	6	0.89	0.01
Calf	pyramidal	6	0.90	0.02
Old	pyramidal	6	0.89	0.01
Adult	complex	1	0.89	0.01
Calf	complex	1	0.89	0.02
Old	complex	1	0.89	0.01
Adult	complex	2	0.87	0.01
Calf	complex	2	0.89	0.01
Old	complex	2	0.87	0.01
Adult	complex	3	0.88	0.01
Calf	complex	3	0.90	0.01
Old	complex	3	0.89	0.01
Adult	complex	4	0.88	0.02
Calf	complex	4	0.89	0.01
Old	complex	4	0.88	0.02
Adult	complex	5	0.87	0.01
Calf	complex	5	0.89	0.02
Old	complex	5	0.88	0.01

Population	Shape	Layer_ID	mean	sd
Adult	complex	6	0.88	0.01
Calf	complex	6	0.89	0.02
Old	complex	6	0.88	0.01

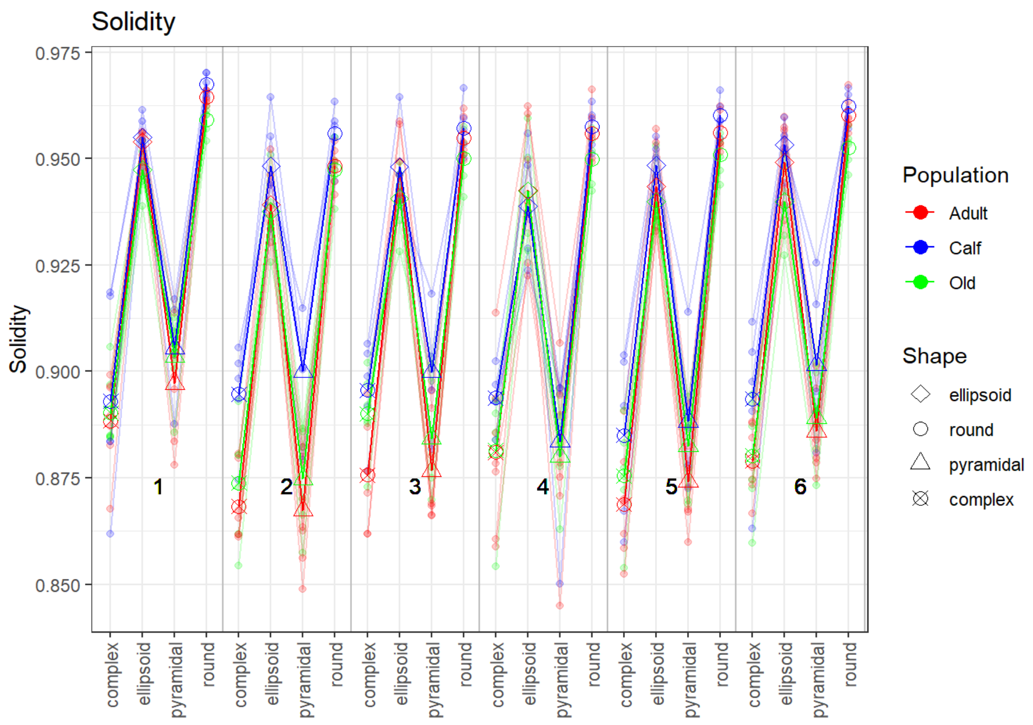

Extent

Population	Shape	Layer_ID	mean	sd
Adult	ellipsoid	1	0.71	0.01
Calf	ellipsoid	1	0.72	0.01
Old	ellipsoid	1	0.71	0.01
Adult	ellipsoid	2	0.69	0.01
Calf	ellipsoid	2	0.70	0.01
Old	ellipsoid	2	0.70	0.01
Adult	ellipsoid	3	0.71	0.00
Calf	ellipsoid	3	0.70	0.01
Old	ellipsoid	3	0.70	0.01
Adult	ellipsoid	4	0.70	0.03
Calf	ellipsoid	4	0.69	0.02
Old	ellipsoid	4	0.70	0.03
Adult	ellipsoid	5	0.70	0.01
Calf	ellipsoid	5	0.71	0.01
Old	ellipsoid	5	0.69	0.01
Adult	ellipsoid	6	0.71	0.01
Calf	ellipsoid	6	0.71	0.01
Old	ellipsoid	6	0.69	0.01
Adult	round	1	0.75	0.00
Calf	round	1	0.76	0.01
Old	round	1	0.73	0.01
Adult	round	2	0.72	0.01
Calf	round	2	0.74	0.02
Old	round	2	0.72	0.01

Population	Shape	Layer_ID	mean	sd
Adult	round	3	0.73	0.01
Calf	round	3	0.74	0.01
Old	round	3	0.72	0.01
Adult	round	4	0.73	0.01
Calf	round	4	0.74	0.01
Old	round	4	0.72	0.01
Adult	round	5	0.74	0.01
Calf	round	5	0.74	0.01
Old	round	5	0.72	0.00
Adult	round	6	0.74	0.01
Calf	round	6	0.74	0.01
Old	round	6	0.73	0.01
Adult	pyramidal	1	0.60	0.01
Calf	pyramidal	1	0.60	0.02
Old	pyramidal	1	0.62	0.01
Adult	pyramidal	2	0.56	0.02
Calf	pyramidal	2	0.59	0.03
Old	pyramidal	2	0.59	0.01
Adult	pyramidal	3	0.57	0.01
Calf	pyramidal	3	0.60	0.03
Old	pyramidal	3	0.59	0.01
Adult	pyramidal	4	0.58	0.03
Calf	pyramidal	4	0.59	0.03
Old	pyramidal	4	0.59	0.01
Adult	pyramidal	5	0.57	0.01
Calf	pyramidal	5	0.60	0.02
Old	pyramidal	5	0.59	0.01
Adult	pyramidal	6	0.59	0.02
Calf	pyramidal	6	0.60	0.03
Old	pyramidal	6	0.60	0.01
Adult	complex	1	0.62	0.01
Calf	complex	1	0.63	0.02
Old	complex	1	0.62	0.01
Adult	complex	2	0.60	0.01
Calf	complex	2	0.62	0.01
Old	complex	2	0.60	0.02
Adult	complex	3	0.60	0.01
Calf	complex	3	0.62	0.01
Old	complex	3	0.61	0.01
Adult	complex	4	0.61	0.03
Calf	complex	4	0.62	0.02
Old	complex	4	0.60	0.02
Adult	complex	5	0.60	0.02
Calf	complex	5	0.61	0.01
Old	complex	5	0.60	0.01
Adult	complex	6	0.61	0.02

Population	Shape	Layer_ID	mean	sd
Calf	complex	6	0.62	0.02
Old	complex	6	0.61	0.01

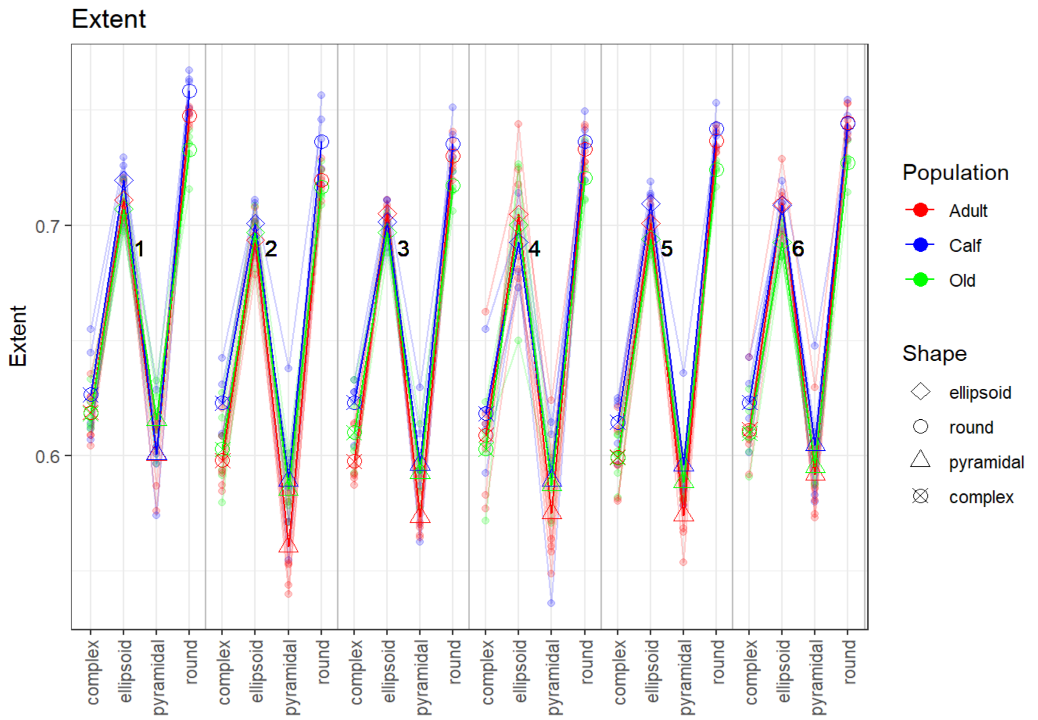

InvAR

Population	Shape	Layer_ID	mean	sd
Adult	ellipsoid	1	0.62	0.00
Calf	ellipsoid	1	0.62	0.00
Old	ellipsoid	1	0.62	0.00
Adult	ellipsoid	2	0.62	0.00
Calf	ellipsoid	2	0.62	0.00
Old	ellipsoid	2	0.62	0.00
Adult	ellipsoid	3	0.62	0.00
Calf	ellipsoid	3	0.62	0.00
Old	ellipsoid	3	0.62	0.00
Adult	ellipsoid	4	0.62	0.01
Calf	ellipsoid	4	0.62	0.01
Old	ellipsoid	4	0.62	0.00
Adult	ellipsoid	5	0.62	0.00
Calf	ellipsoid	5	0.62	0.00
Old	ellipsoid	5	0.62	0.00
Adult	ellipsoid	6	0.62	0.00
Calf	ellipsoid	6	0.62	0.00
Old	ellipsoid	6	0.62	0.00
Adult	round	1	0.80	0.01
Calf	round	1	0.82	0.02
Old	round	1	0.80	0.01
Adult	round	2	0.79	0.01
Calf	round	2	0.80	0.01
Old	round	2	0.78	0.01
Adult	round	3	0.80	0.01

Population	Shape	Layer_ID	mean	sd
Calf	round	3	0.80	0.01
Old	round	3	0.79	0.01
Adult	round	4	0.79	0.01
Calf	round	4	0.80	0.01
Old	round	4	0.80	0.01
Adult	round	5	0.80	0.01
Calf	round	5	0.81	0.01
Old	round	5	0.79	0.01
Adult	round	6	0.80	0.01
Calf	round	6	0.81	0.01
Old	round	6	0.79	0.00
Adult	pyramidal	1	0.47	0.01
Calf	pyramidal	1	0.46	0.02
Old	pyramidal	1	0.50	0.02
Adult	pyramidal	2	0.48	0.02
Calf	pyramidal	2	0.48	0.02
Old	pyramidal	2	0.48	0.02
Adult	pyramidal	3	0.48	0.01
Calf	pyramidal	3	0.49	0.00
Old	pyramidal	3	0.50	0.01
Adult	pyramidal	4	0.49	0.02
Calf	pyramidal	4	0.49	0.02
Old	pyramidal	4	0.49	0.02
Adult	pyramidal	5	0.48	0.01
Calf	pyramidal	5	0.49	0.01
Old	pyramidal	5	0.49	0.01
Adult	pyramidal	6	0.47	0.02
Calf	pyramidal	6	0.49	0.01
Old	pyramidal	6	0.49	0.02
Adult	complex	1	0.74	0.01
Calf	complex	1	0.75	0.01
Old	complex	1	0.74	0.02
Adult	complex	2	0.75	0.01
Calf	complex	2	0.74	0.01
Old	complex	2	0.74	0.00
Adult	complex	3	0.75	0.01
Calf	complex	3	0.74	0.01
Old	complex	3	0.75	0.00
Adult	complex	4	0.74	0.01
Calf	complex	4	0.74	0.02
Old	complex	4	0.74	0.02
Adult	complex	5	0.75	0.01
Calf	complex	5	0.74	0.01
Old	complex	5	0.75	0.01
Adult	complex	6	0.74	0.01
Calf	complex	6	0.75	0.01

Population	Shape	Layer_ID	mean	sd
Old	complex	6	0.74	0.01

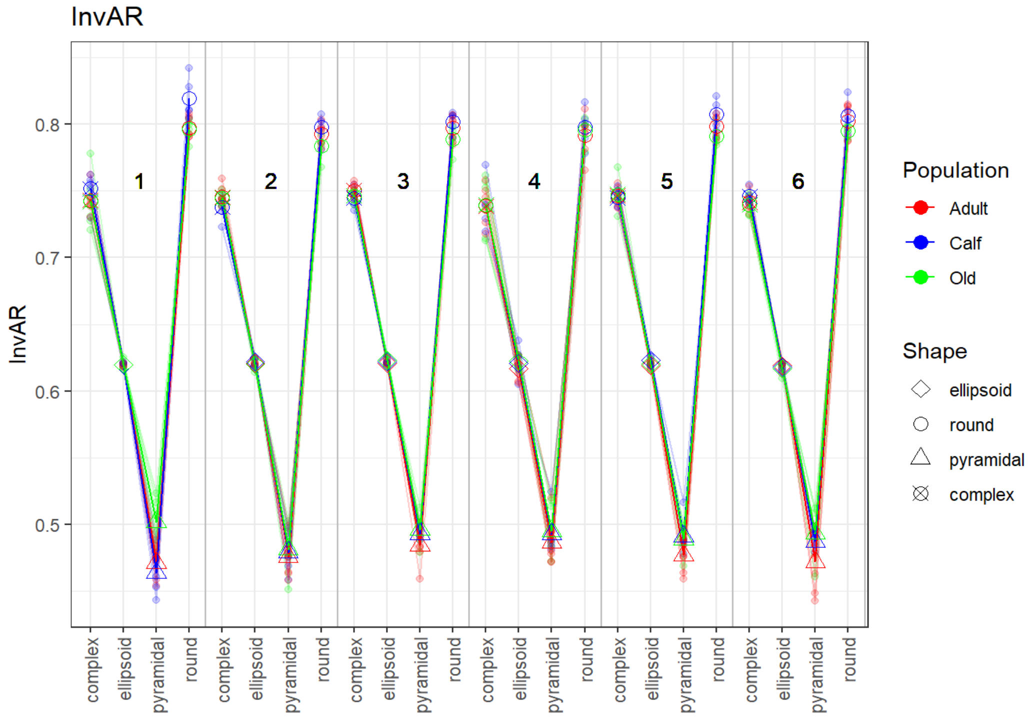

ConvexCircularity

Population	Shape	Layer_ID	mean	sd
Adult	ellipsoid	1	0.89	0.00
Calf	ellipsoid	1	0.89	0.00
Old	ellipsoid	1	0.89	0.00
Adult	ellipsoid	2	0.89	0.00
Calf	ellipsoid	2	0.89	0.00
Old	ellipsoid	2	0.89	0.00
Adult	ellipsoid	3	0.89	0.00
Calf	ellipsoid	3	0.89	0.00
Old	ellipsoid	3	0.89	0.00
Adult	ellipsoid	4	0.89	0.00
Calf	ellipsoid	4	0.89	0.01
Old	ellipsoid	4	0.89	0.00
Adult	ellipsoid	5	0.89	0.00
Calf	ellipsoid	5	0.89	0.00
Old	ellipsoid	5	0.89	0.00
Adult	ellipsoid	6	0.89	0.00
Calf	ellipsoid	6	0.89	0.00
Old	ellipsoid	6	0.89	0.00
Adult	round	1	0.94	0.00
Calf	round	1	0.94	0.01
Old	round	1	0.93	0.00
Adult	round	2	0.92	0.00
Calf	round	2	0.93	0.01
Old	round	2	0.92	0.00
Adult	round	3	0.93	0.00
Calf	round	3	0.93	0.00

Population	Shape	Layer_ID	mean	sd
Old	round	3	0.92	0.00
Adult	round	4	0.93	0.00
Calf	round	4	0.93	0.00
Old	round	4	0.93	0.00
Adult	round	5	0.93	0.00
Calf	round	5	0.93	0.00
Old	round	5	0.93	0.00
Adult	round	6	0.94	0.00
Calf	round	6	0.94	0.00
Old	round	6	0.93	0.00
Adult	pyramidal	1	0.77	0.01
Calf	pyramidal	1	0.76	0.02
Old	pyramidal	1	0.79	0.01
Adult	pyramidal	2	0.75	0.02
Calf	pyramidal	2	0.76	0.02
Old	pyramidal	2	0.76	0.02
Adult	pyramidal	3	0.76	0.01
Calf	pyramidal	3	0.77	0.01
Old	pyramidal	3	0.77	0.02
Adult	pyramidal	4	0.77	0.01
Calf	pyramidal	4	0.77	0.02
Old	pyramidal	4	0.77	0.02
Adult	pyramidal	5	0.76	0.01
Calf	pyramidal	5	0.77	0.02
Old	pyramidal	5	0.77	0.01
Adult	pyramidal	6	0.76	0.02
Calf	pyramidal	6	0.77	0.01
Old	pyramidal	6	0.78	0.01
Adult	complex	1	0.85	0.00
Calf	complex	1	0.85	0.01
Old	complex	1	0.85	0.00
Adult	complex	2	0.84	0.01
Calf	complex	2	0.84	0.00
Old	complex	2	0.84	0.00
Adult	complex	3	0.83	0.00
Calf	complex	3	0.84	0.00
Old	complex	3	0.84	0.01
Adult	complex	4	0.84	0.01
Calf	complex	4	0.84	0.01
Old	complex	4	0.84	0.01
Adult	complex	5	0.84	0.01
Calf	complex	5	0.84	0.01
Old	complex	5	0.84	0.00
Adult	complex	6	0.84	0.00
Calf	complex	6	0.84	0.00
Old	complex	6	0.84	0.01

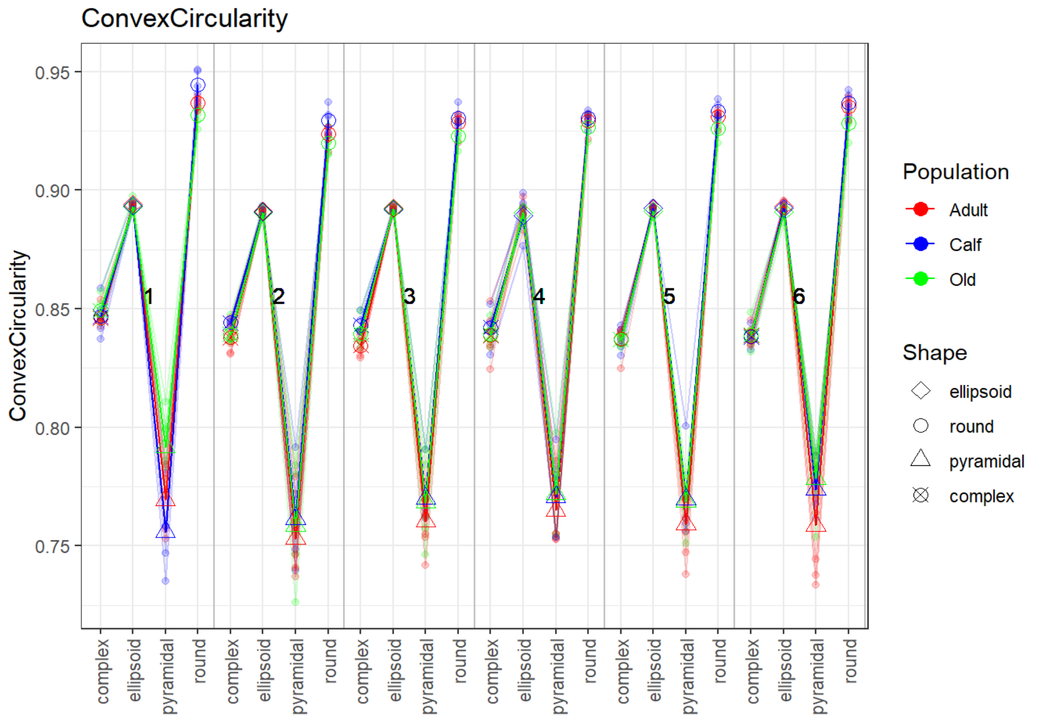

Radar Plot

Density

Principal Components

Layer 1

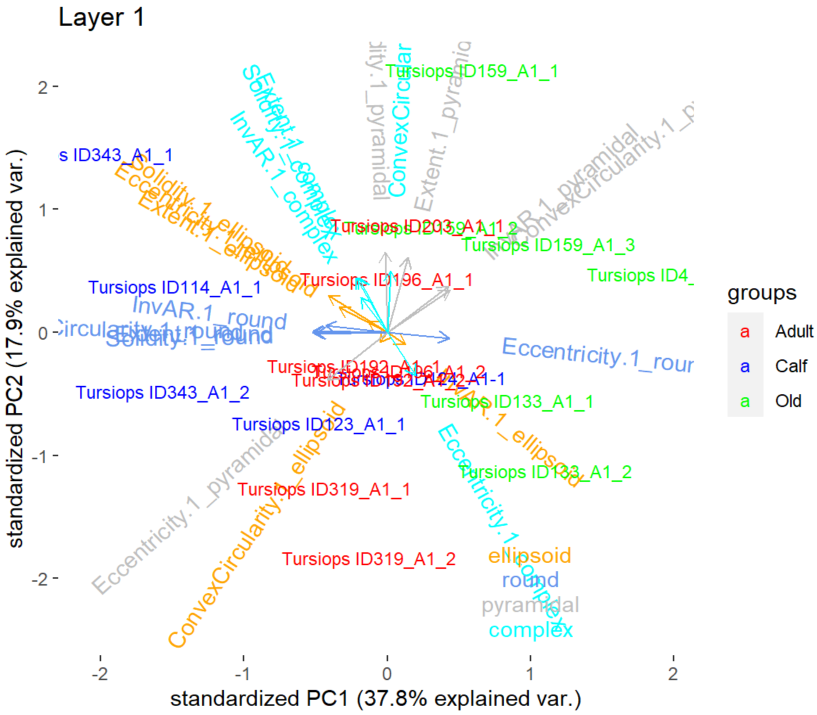

Layer 2

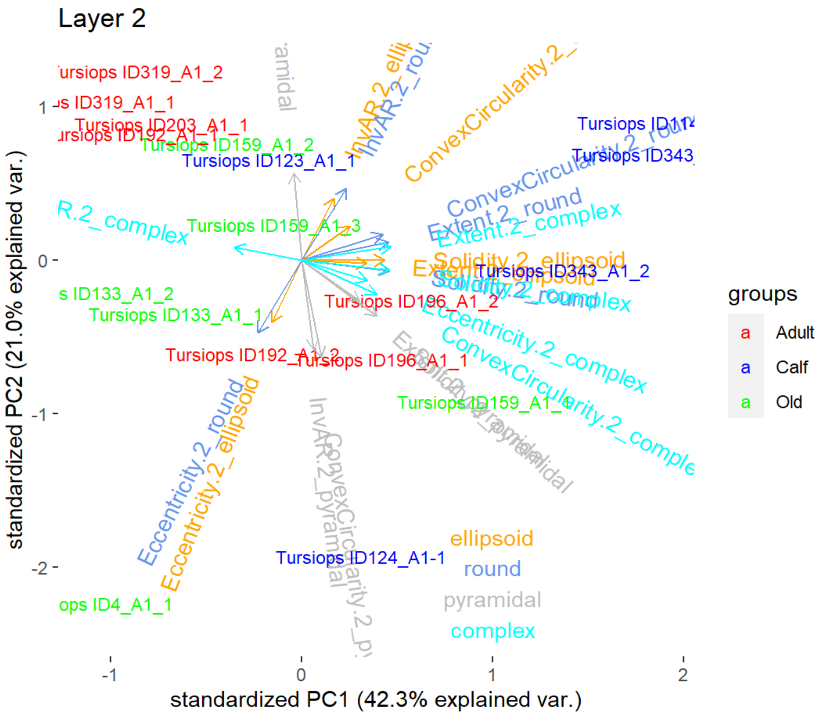

Layer 3

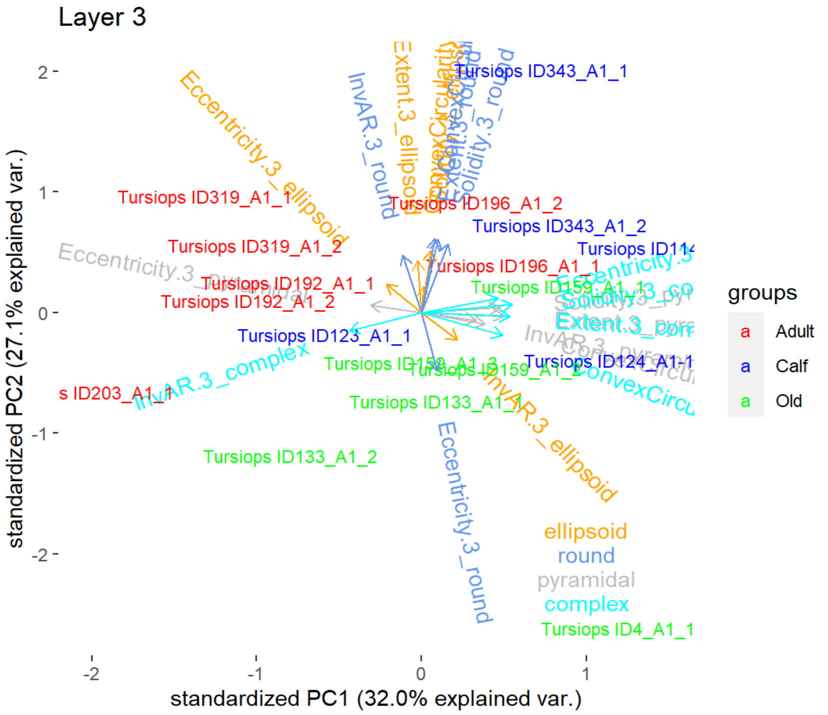

Layer 4

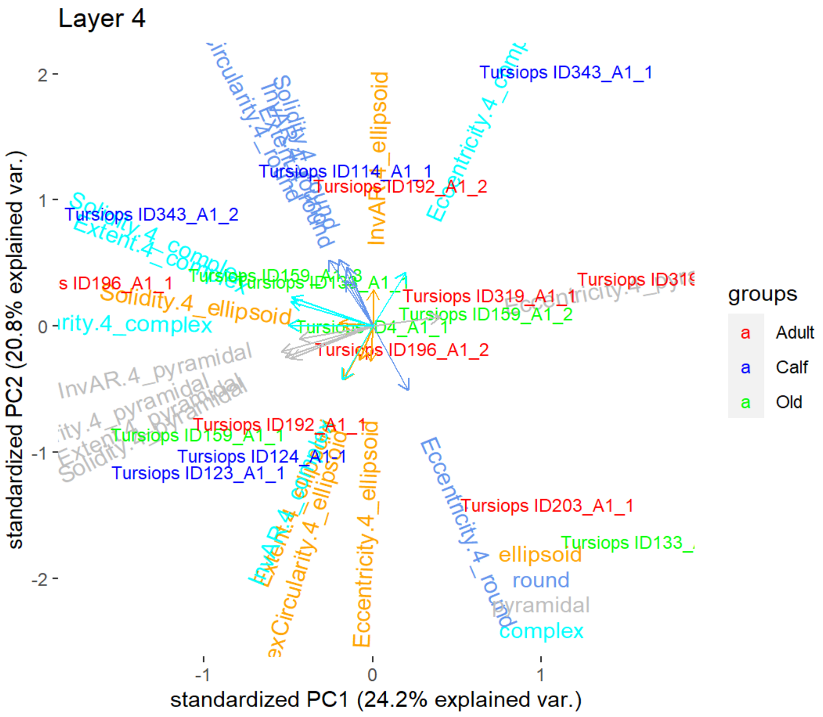

Layer 5

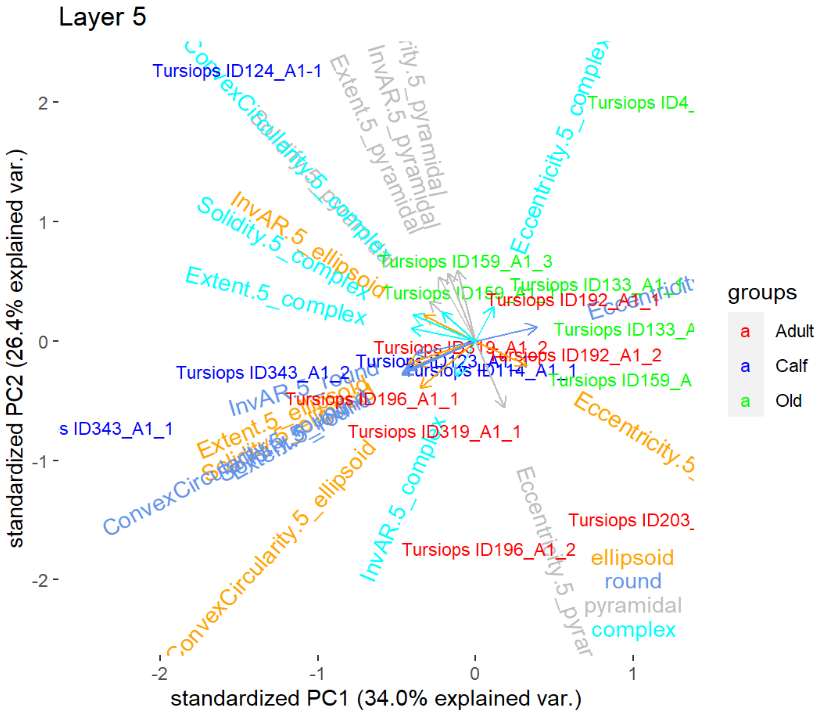

Layer 6

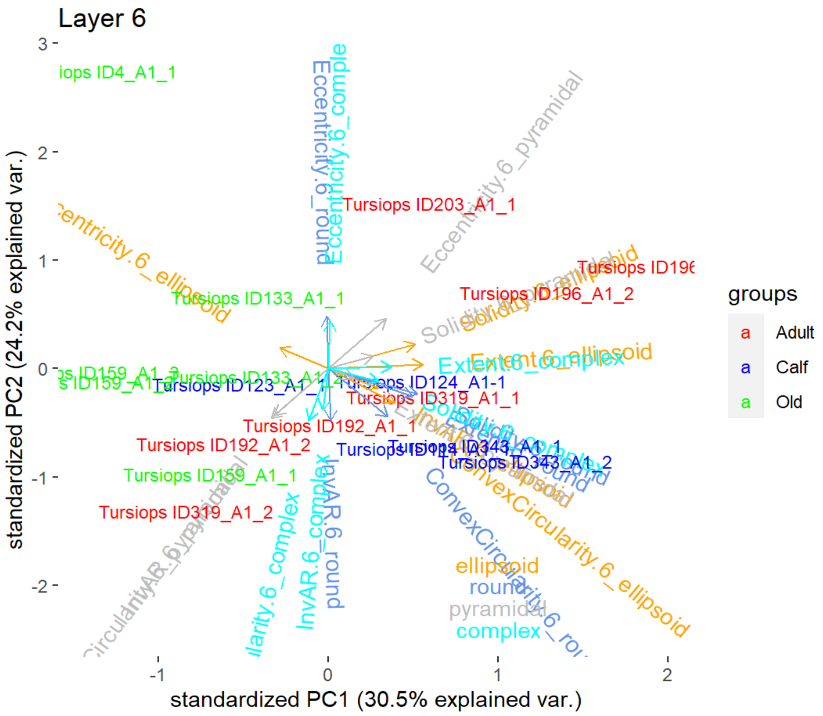

Inferential Analysis

Univariate

```
FALSE  Call:
FALSE  flip(Y = . ~ Population, data = DW, perms = nperms)
FALSE 4999 permutations.
FALSE
FALSE                                                     Test    Stat tail
FALSE Eccentricity.1_ellipsoid_|_Population.Calf.         t  0.2656   ><
FALSE Eccentricity.1_ellipsoid_|_Population.Old.          t -0.2740   ><
FALSE Solidity.1_ellipsoid_|_Population.Calf.             t  1.4413   ><
FALSE Solidity.1_ellipsoid_|_Population.Old.              t -3.0838   ><
FALSE Extent.1_ellipsoid_|_Population.Calf.               t  2.1990   ><
FALSE Extent.1_ellipsoid_|_Population.Old.                t -1.5647   ><
FALSE InvAR.1_ellipsoid_|_Population.Calf.                t -0.2231   ><
FALSE InvAR.1_ellipsoid_|_Population.Old.                 t  0.1988   ><
FALSE ConvexCircularity.1_ellipsoid_|_Population.Calf.    t -0.2851   ><
FALSE ConvexCircularity.1_ellipsoid_|_Population.Old.     t -1.0658   ><
FALSE Eccentricity.2_ellipsoid_|_Population.Calf.         t -0.9723   ><
FALSE Eccentricity.2_ellipsoid_|_Population.Old.          t  0.8726   ><
FALSE Solidity.2_ellipsoid_|_Population.Calf.             t  2.0513   ><
FALSE Solidity.2_ellipsoid_|_Population.Old.              t -1.0775   ><
FALSE Extent.2_ellipsoid_|_Population.Calf.               t  1.0952   ><
FALSE Extent.2_ellipsoid_|_Population.Old.                t  0.0369   ><
FALSE InvAR.2_ellipsoid_|_Population.Calf.                t  1.0372   ><
FALSE InvAR.2_ellipsoid_|_Population.Old.                 t -0.8442   ><
FALSE ConvexCircularity.2_ellipsoid_|_Population.Calf.    t -0.0247   ><
FALSE ConvexCircularity.2_ellipsoid_|_Population.Old.     t -0.8324   ><
FALSE Eccentricity.3_ellipsoid_|_Population.Calf.         t -0.0284   ><
FALSE Eccentricity.3_ellipsoid_|_Population.Old.          t -1.6332   ><
FALSE Solidity.3_ellipsoid_|_Population.Calf.             t  0.7397   ><
FALSE Solidity.3_ellipsoid_|_Population.Old.              t -1.8144   ><
FALSE Extent.3_ellipsoid_|_Population.Calf.               t  0.0191   ><
FALSE Extent.3_ellipsoid_|_Population.Old.                t -1.9424   ><
FALSE InvAR.3_ellipsoid_|_Population.Calf.                t  0.0556   ><
FALSE InvAR.3_ellipsoid_|_Population.Old.                 t  1.5334   ><
FALSE ConvexCircularity.3_ellipsoid_|_Population.Calf.    t -0.4695   ><
FALSE ConvexCircularity.3_ellipsoid_|_Population.Old.     t -0.3354   ><
FALSE Eccentricity.4_ellipsoid_|_Population.Calf.         t -0.3588   ><
FALSE Eccentricity.4_ellipsoid_|_Population.Old.          t -0.7266   ><
FALSE Solidity.4_ellipsoid_|_Population.Calf.             t -0.4963   ><
FALSE Solidity.4_ellipsoid_|_Population.Old.              t  0.1891   ><
FALSE Extent.4_ellipsoid_|_Population.Calf.               t -0.7796   ><
FALSE Extent.4_ellipsoid_|_Population.Old.                t  0.0330   ><
FALSE InvAR.4_ellipsoid_|_Population.Calf.                t  0.3681   ><
FALSE InvAR.4_ellipsoid_|_Population.Old.                 t  0.7129   ><
FALSE ConvexCircularity.4_ellipsoid_|_Population.Calf.    t -0.4989   ><
FALSE ConvexCircularity.4_ellipsoid_|_Population.Old.     t  0.1704   ><
FALSE Eccentricity.5_ellipsoid_|_Population.Calf.         t -2.8174   ><
FALSE Eccentricity.5_ellipsoid_|_Population.Old.          t  0.4006   ><
FALSE Solidity.5_ellipsoid_|_Population.Calf.             t  1.5700   ><
FALSE Solidity.5_ellipsoid_|_Population.Old.              t -1.3830   ><
FALSE Extent.5_ellipsoid_|_Population.Calf.               t  2.7533   ><
FALSE Extent.5_ellipsoid_|_Population.Old.                t -2.5521   ><
FALSE InvAR.5_ellipsoid_|_Population.Calf.                t  2.8935   ><
FALSE InvAR.5_ellipsoid_|_Population.Old.                 t -0.3768   ><
FALSE ConvexCircularity.5_ellipsoid_|_Population.Calf.    t  1.2185   ><
FALSE ConvexCircularity.5_ellipsoid_|_Population.Old.     t -2.0753   ><
FALSE Eccentricity.6_ellipsoid_|_Population.Calf.         t  0.0868   ><
FALSE Eccentricity.6_ellipsoid_|_Population.Old.          t  0.9156   ><
FALSE Solidity.6_ellipsoid_|_Population.Calf.             t  1.6672   ><
FALSE Solidity.6_ellipsoid_|_Population.Old.              t -2.5090   ><
FALSE Extent.6_ellipsoid_|_Population.Calf.               t  1.1293   ><
FALSE Extent.6_ellipsoid_|_Population.Old.                t -3.5644   ><
FALSE InvAR.6_ellipsoid_|_Population.Calf.                t -0.0300   ><
FALSE InvAR.6_ellipsoid_|_Population.Old.                 t -0.9208   ><
FALSE ConvexCircularity.6_ellipsoid_|_Population.Calf.    t  0.0965   ><
FALSE ConvexCircularity.6_ellipsoid_|_Population.Old.     t -1.7958   ><
FALSE Eccentricity.1_round_|_Population.Calf.             t -3.8475   ><
FALSE Eccentricity.1_round_|_Population.Old.              t  1.5256   ><
FALSE Solidity.1_round_|_Population.Calf.                 t  3.0261   ><
FALSE Solidity.1_round_|_Population.Old.                  t -4.7223   ><
FALSE Extent.1_round_|_Population.Calf.                   t  3.4041   ><
FALSE Extent.1_round_|_Population.Old.                    t -4.5112   ><
FALSE InvAR.1_round_|_Population.Calf.                    t  4.0825   ><
FALSE InvAR.1_round_|_Population.Old.                     t -1.4889   ><
FALSE ConvexCircularity.1_round_|_Population.Calf.        t  4.0524   ><
FALSE ConvexCircularity.1_round_|_Population.Old.         t -3.2603   ><
FALSE Eccentricity.2_round_|_Population.Calf.             t -1.8611   ><
FALSE Eccentricity.2_round_|_Population.Old.              t  2.6811   ><
FALSE Solidity.2_round_|_Population.Calf.                 t  2.7829   ><
```

```
FALSE Solidity.2_round_|_Population.Old.                t -1.2632   ><
FALSE Extent.2_round_|_Population.Calf.                 t  3.4690   ><
FALSE Extent.2_round_|_Population.Old.                  t -1.6115   ><
FALSE InvAR.2_round_|_Population.Calf.                  t  1.8226   ><
FALSE InvAR.2_round_|_Population.Old.                   t -2.4768   ><
FALSE ConvexCircularity.2_round_|_Population.Calf.      t  3.0031   ><
FALSE ConvexCircularity.2_round_|_Population.Old.       t -2.3914   ><
FALSE Eccentricity.3_round_|_Population.Calf.           t -1.8489   ><
FALSE Eccentricity.3_round_|_Population.Old.            t  2.3101   ><
FALSE Solidity.3_round_|_Population.Calf.               t  1.4738   ><
FALSE Solidity.3_round_|_Population.Old.                t -2.0799   ><
FALSE Extent.3_round_|_Population.Calf.                 t  2.0559   ><
FALSE Extent.3_round_|_Population.Old.                  t -3.4501   ><
FALSE InvAR.3_round_|_Population.Calf.                  t  1.7046   ><
FALSE InvAR.3_round_|_Population.Old.                   t -2.3041   ><
FALSE ConvexCircularity.3_round_|_Population.Calf.      t  1.8372   ><
FALSE ConvexCircularity.3_round_|_Population.Old.       t -3.1593   ><
FALSE Eccentricity.4_round_|_Population.Calf.           t -0.6922   ><
FALSE Eccentricity.4_round_|_Population.Old.            t -0.1096   ><
FALSE Solidity.4_round_|_Population.Calf.               t  1.2012   ><
FALSE Solidity.4_round_|_Population.Old.                t -2.0312   ><
FALSE Extent.4_round_|_Population.Calf.                 t  1.4145   ><
FALSE Extent.4_round_|_Population.Old.                  t -2.4888   ><
FALSE InvAR.4_round_|_Population.Calf.                  t  0.6131   ><
FALSE InvAR.4_round_|_Population.Old.                   t  0.2016   ><
FALSE ConvexCircularity.4_round_|_Population.Calf.      t  1.2624   ><
FALSE ConvexCircularity.4_round_|_Population.Old.       t -1.4961   ><
FALSE Eccentricity.5_round_|_Population.Calf.           t -2.5321   ><
FALSE Eccentricity.5_round_|_Population.Old.            t  2.5166   ><
FALSE Solidity.5_round_|_Population.Calf.               t  2.4136   ><
FALSE Solidity.5_round_|_Population.Old.                t -2.8805   ><
FALSE Extent.5_round_|_Population.Calf.                 t  2.6611   ><
FALSE Extent.5_round_|_Population.Old.                  t -5.0035   ><
FALSE InvAR.5_round_|_Population.Calf.                  t  2.5533   ><
FALSE InvAR.5_round_|_Population.Old.                   t -2.3147   ><
FALSE ConvexCircularity.5_round_|_Population.Calf.      t  2.1404   ><
FALSE ConvexCircularity.5_round_|_Population.Old.       t -3.5720   ><
FALSE Eccentricity.6_round_|_Population.Calf.           t -1.2629   ><
FALSE Eccentricity.6_round_|_Population.Old.            t  1.6950   ><
FALSE Solidity.6_round_|_Population.Calf.               t  2.0154   ><
FALSE Solidity.6_round_|_Population.Old.                t -4.2672   ><
FALSE Extent.6_round_|_Population.Calf.                 t  1.4144   ><
FALSE Extent.6_round_|_Population.Old.                  t -5.1544   ><
FALSE InvAR.6_round_|_Population.Calf.                  t  1.2922   ><
FALSE InvAR.6_round_|_Population.Old.                   t -1.7378   ><
FALSE ConvexCircularity.6_round_|_Population.Calf.      t  1.7949   ><
FALSE ConvexCircularity.6_round_|_Population.Old.       t -3.6417   ><
FALSE Eccentricity.1_pyramidal_|_Population.Calf.       t  1.8942   ><
FALSE Eccentricity.1_pyramidal_|_Population.Old.        t -4.4392   ><
FALSE Solidity.1_pyramidal_|_Population.Calf.           t  0.8737   ><
FALSE Solidity.1_pyramidal_|_Population.Old.            t  0.4692   ><
FALSE Extent.1_pyramidal_|_Population.Calf.             t -0.7715   ><
FALSE Extent.1_pyramidal_|_Population.Old.              t  1.9629   ><
FALSE InvAR.1_pyramidal_|_Population.Calf.              t -1.9781   ><
FALSE InvAR.1_pyramidal_|_Population.Old.               t  4.4196   ><
FALSE ConvexCircularity.1_pyramidal_|_Population.Calf.  t -2.6394   ><
FALSE ConvexCircularity.1_pyramidal_|_Population.Old.   t  3.7061   ><
FALSE Eccentricity.2_pyramidal_|_Population.Calf.       t  0.1366   ><
FALSE Eccentricity.2_pyramidal_|_Population.Old.        t -0.5654   ><
FALSE Solidity.2_pyramidal_|_Population.Calf.           t  4.2657   ><
FALSE Solidity.2_pyramidal_|_Population.Old.            t -0.6498   ><
FALSE Extent.2_pyramidal_|_Population.Calf.             t  1.3957   ><
FALSE Extent.2_pyramidal_|_Population.Old.              t  1.0609   ><
FALSE InvAR.2_pyramidal_|_Population.Calf.              t  0.0863   ><
FALSE InvAR.2_pyramidal_|_Population.Old.               t  0.5235   ><
FALSE ConvexCircularity.2_pyramidal_|_Population.Calf.  t  0.5840   ><
FALSE ConvexCircularity.2_pyramidal_|_Population.Old.   t  0.1814   ><
FALSE Eccentricity.3_pyramidal_|_Population.Calf.       t -0.3205   ><
FALSE Eccentricity.3_pyramidal_|_Population.Old.        t -1.6340   ><
FALSE Solidity.3_pyramidal_|_Population.Calf.           t  2.8326   ><
FALSE Solidity.3_pyramidal_|_Population.Old.            t -0.2613   ><
FALSE Extent.3_pyramidal_|_Population.Calf.             t  1.4583   ><
FALSE Extent.3_pyramidal_|_Population.Old.              t  1.0157   ><
FALSE InvAR.3_pyramidal_|_Population.Calf.              t  0.4511   ><
FALSE InvAR.3_pyramidal_|_Population.Old.               t  1.4323   ><
FALSE ConvexCircularity.3_pyramidal_|_Population.Calf.  t  0.8099   ><
FALSE ConvexCircularity.3_pyramidal_|_Population.Old.   t  0.5523   ><
FALSE Eccentricity.4_pyramidal_|_Population.Calf.       t -0.3250   ><
```

```
FALSE Eccentricity.4_pyramidal_|_Population.Old.          t -0.5748   ><
FALSE Solidity.4_pyramidal_|_Population.Calf.             t  0.3849   ><
FALSE Solidity.4_pyramidal_|_Population.Old.              t -0.1548   ><
FALSE Extent.4_pyramidal_|_Population.Calf.               t  0.6791   ><
FALSE Extent.4_pyramidal_|_Population.Old.                t  0.4438   ><
FALSE InvAR.4_pyramidal_|_Population.Calf.                t  0.2771   ><
FALSE InvAR.4_pyramidal_|_Population.Old.                 t  0.6069   ><
FALSE ConvexCircularity.4_pyramidal_|_Population.Calf.    t  0.3148   ><
FALSE ConvexCircularity.4_pyramidal_|_Population.Old.     t  0.5044   ><
FALSE Eccentricity.5_pyramidal_|_Population.Calf.         t -1.1958   ><
FALSE Eccentricity.5_pyramidal_|_Population.Old.          t -0.9743   ><
FALSE Solidity.5_pyramidal_|_Population.Calf.             t  1.5087   ><
FALSE Solidity.5_pyramidal_|_Population.Old.              t  0.3592   ><
FALSE Extent.5_pyramidal_|_Population.Calf.               t  1.7816   ><
FALSE Extent.5_pyramidal_|_Population.Old.                t  0.6013   ><
FALSE InvAR.5_pyramidal_|_Population.Calf.                t  1.1576   ><
FALSE InvAR.5_pyramidal_|_Population.Old.                 t  0.8894   ><
FALSE ConvexCircularity.5_pyramidal_|_Population.Calf.    t  0.8029   ><
FALSE ConvexCircularity.5_pyramidal_|_Population.Old.     t  0.7244   ><
FALSE Eccentricity.6_pyramidal_|_Population.Calf.         t -0.3690   ><
FALSE Eccentricity.6_pyramidal_|_Population.Old.          t -1.6550   ><
FALSE Solidity.6_pyramidal_|_Population.Calf.             t  2.0630   ><
FALSE Solidity.6_pyramidal_|_Population.Old.              t -0.4583   ><
FALSE Extent.6_pyramidal_|_Population.Calf.               t  1.1131   ><
FALSE Extent.6_pyramidal_|_Population.Old.                t -0.1876   ><
FALSE InvAR.6_pyramidal_|_Population.Calf.                t  0.5085   ><
FALSE InvAR.6_pyramidal_|_Population.Old.                 t  1.6293   ><
FALSE ConvexCircularity.6_pyramidal_|_Population.Calf.    t  0.6447   ><
FALSE ConvexCircularity.6_pyramidal_|_Population.Old.     t  1.5624   ><
FALSE Eccentricity.1_complex_|_Population.Calf.           t -1.0242   ><
FALSE Eccentricity.1_complex_|_Population.Old.            t  0.6231   ><
FALSE Solidity.1_complex_|_Population.Calf.               t  0.4626   ><
FALSE Solidity.1_complex_|_Population.Old.                t -0.0115   ><
FALSE Extent.1_complex_|_Population.Calf.                 t  1.0497   ><
FALSE Extent.1_complex_|_Population.Old.                  t -0.5031   ><
FALSE InvAR.1_complex_|_Population.Calf.                  t  1.1427   ><
FALSE InvAR.1_complex_|_Population.Old.                   t -0.5714   ><
FALSE ConvexCircularity.1_complex_|_Population.Calf.      t -0.2749   ><
FALSE ConvexCircularity.1_complex_|_Population.Old.       t  1.0859   ><
FALSE Eccentricity.2_complex_|_Population.Calf.           t  1.8756   ><
FALSE Eccentricity.2_complex_|_Population.Old.            t -0.3899   ><
FALSE Solidity.2_complex_|_Population.Calf.               t  3.8566   ><
FALSE Solidity.2_complex_|_Population.Old.                t -0.6720   ><
FALSE Extent.2_complex_|_Population.Calf.                 t  2.8463   ><
FALSE Extent.2_complex_|_Population.Old.                  t -0.5858   ><
FALSE InvAR.2_complex_|_Population.Calf.                  t -1.8810   ><
FALSE InvAR.2_complex_|_Population.Old.                   t  0.5597   ><
FALSE ConvexCircularity.2_complex_|_Population.Calf.      t  2.4202   ><
FALSE ConvexCircularity.2_complex_|_Population.Old.       t -0.4677   ><
FALSE                                                p-value sig.
FALSE Eccentricity.1_ellipsoid_|_Population.Calf.       0.7866
FALSE Eccentricity.1_ellipsoid_|_Population.Old.        0.7924
FALSE Solidity.1_ellipsoid_|_Population.Calf.           0.1602
FALSE Solidity.1_ellipsoid_|_Population.Old.            0.0054   **
FALSE Extent.1_ellipsoid_|_Population.Calf.             0.0428    *
FALSE Extent.1_ellipsoid_|_Population.Old.              0.1276
FALSE InvAR.1_ellipsoid_|_Population.Calf.              0.8256
FALSE InvAR.1_ellipsoid_|_Population.Old.               0.8454
FALSE ConvexCircularity.1_ellipsoid_|_Population.Calf.  0.7728
FALSE ConvexCircularity.1_ellipsoid_|_Population.Old.   0.2884
FALSE Eccentricity.2_ellipsoid_|_Population.Calf.       0.3272
FALSE Eccentricity.2_ellipsoid_|_Population.Old.        0.3856
FALSE Solidity.2_ellipsoid_|_Population.Calf.           0.0488    *
FALSE Solidity.2_ellipsoid_|_Population.Old.            0.2906
FALSE Extent.2_ellipsoid_|_Population.Calf.             0.2818
FALSE Extent.2_ellipsoid_|_Population.Old.              0.9756
FALSE InvAR.2_ellipsoid_|_Population.Calf.              0.3020
FALSE InvAR.2_ellipsoid_|_Population.Old.               0.3972
FALSE ConvexCircularity.2_ellipsoid_|_Population.Calf.  0.9772
FALSE ConvexCircularity.2_ellipsoid_|_Population.Old.   0.3874
FALSE Eccentricity.3_ellipsoid_|_Population.Calf.       0.9756
FALSE Eccentricity.3_ellipsoid_|_Population.Old.        0.1090
FALSE Solidity.3_ellipsoid_|_Population.Calf.           0.4610
FALSE Solidity.3_ellipsoid_|_Population.Old.            0.0796
FALSE Extent.3_ellipsoid_|_Population.Calf.             0.9864
FALSE Extent.3_ellipsoid_|_Population.Old.              0.0654
FALSE InvAR.3_ellipsoid_|_Population.Calf.              0.9580
FALSE InvAR.3_ellipsoid_|_Population.Old.               0.1310
```

```
FALSE ConvexCircularity.3_ellipsoid_|_Population.Calf.  0.6482
FALSE ConvexCircularity.3_ellipsoid_|_Population.Old.   0.7316
FALSE Eccentricity.4_ellipsoid_|_Population.Calf.       0.7176
FALSE Eccentricity.4_ellipsoid_|_Population.Old.        0.4814
FALSE Solidity.4_ellipsoid_|_Population.Calf.           0.6196
FALSE Solidity.4_ellipsoid_|_Population.Old.            0.8580
FALSE Extent.4_ellipsoid_|_Population.Calf.             0.4254
FALSE Extent.4_ellipsoid_|_Population.Old.              0.9752
FALSE InvAR.4_ellipsoid_|_Population.Calf.              0.7092
FALSE InvAR.4_ellipsoid_|_Population.Old.               0.4864
FALSE ConvexCircularity.4_ellipsoid_|_Population.Calf.  0.6196
FALSE ConvexCircularity.4_ellipsoid_|_Population.Old.   0.8636
FALSE Eccentricity.5_ellipsoid_|_Population.Calf.       0.0090   **
FALSE Eccentricity.5_ellipsoid_|_Population.Old.        0.6734
FALSE Solidity.5_ellipsoid_|_Population.Calf.           0.1280
FALSE Solidity.5_ellipsoid_|_Population.Old.            0.1724
FALSE Extent.5_ellipsoid_|_Population.Calf.             0.0116    *
FALSE Extent.5_ellipsoid_|_Population.Old.              0.0224    *
FALSE InvAR.5_ellipsoid_|_Population.Calf.              0.0076   **
FALSE InvAR.5_ellipsoid_|_Population.Old.               0.6894
FALSE ConvexCircularity.5_ellipsoid_|_Population.Calf.  0.2224
FALSE ConvexCircularity.5_ellipsoid_|_Population.Old.   0.0462    *
FALSE Eccentricity.6_ellipsoid_|_Population.Calf.       0.9358
FALSE Eccentricity.6_ellipsoid_|_Population.Old.        0.3630
FALSE Solidity.6_ellipsoid_|_Population.Calf.           0.1096
FALSE Solidity.6_ellipsoid_|_Population.Old.            0.0208    *
FALSE Extent.6_ellipsoid_|_Population.Calf.             0.2630
FALSE Extent.6_ellipsoid_|_Population.Old.              0.0034   **
FALSE InvAR.6_ellipsoid_|_Population.Calf.              0.9762
FALSE InvAR.6_ellipsoid_|_Population.Old.               0.3580
FALSE ConvexCircularity.6_ellipsoid_|_Population.Calf.  0.9222
FALSE ConvexCircularity.6_ellipsoid_|_Population.Old.   0.0802
FALSE Eccentricity.1_round_|_Population.Calf.           0.0012   **
FALSE Eccentricity.1_round_|_Population.Old.            0.1254
FALSE Solidity.1_round_|_Population.Calf.               0.0066   **
FALSE Solidity.1_round_|_Population.Old.                0.0004  ***
FALSE Extent.1_round_|_Population.Calf.                 0.0016   **
FALSE Extent.1_round_|_Population.Old.                  0.0004  ***
FALSE InvAR.1_round_|_Population.Calf.                  0.0008  ***
FALSE InvAR.1_round_|_Population.Old.                   0.1390
FALSE ConvexCircularity.1_round_|_Population.Calf.      0.0010  ***
FALSE ConvexCircularity.1_round_|_Population.Old.       0.0040   **
FALSE Eccentricity.2_round_|_Population.Calf.           0.0722
FALSE Eccentricity.2_round_|_Population.Old.            0.0162    *
FALSE Solidity.2_round_|_Population.Calf.               0.0144    *
FALSE Solidity.2_round_|_Population.Old.                0.2216
FALSE Extent.2_round_|_Population.Calf.                 0.0052   **
FALSE Extent.2_round_|_Population.Old.                  0.1180
FALSE InvAR.2_round_|_Population.Calf.                  0.0790
FALSE InvAR.2_round_|_Population.Old.                   0.0248    *
FALSE ConvexCircularity.2_round_|_Population.Calf.      0.0062   **
FALSE ConvexCircularity.2_round_|_Population.Old.       0.0246    *
FALSE Eccentricity.3_round_|_Population.Calf.           0.0702
FALSE Eccentricity.3_round_|_Population.Old.            0.0332    *
FALSE Solidity.3_round_|_Population.Calf.               0.1524
FALSE Solidity.3_round_|_Population.Old.                0.0530
FALSE Extent.3_round_|_Population.Calf.                 0.0488    *
FALSE Extent.3_round_|_Population.Old.                  0.0028   **
FALSE InvAR.3_round_|_Population.Calf.                  0.0932
FALSE InvAR.3_round_|_Population.Old.                   0.0334    *
FALSE ConvexCircularity.3_round_|_Population.Calf.      0.0792
FALSE ConvexCircularity.3_round_|_Population.Old.       0.0044   **
FALSE Eccentricity.4_round_|_Population.Calf.           0.4752
FALSE Eccentricity.4_round_|_Population.Old.            0.9042
FALSE Solidity.4_round_|_Population.Calf.               0.2400
FALSE Solidity.4_round_|_Population.Old.                0.0604
FALSE Extent.4_round_|_Population.Calf.                 0.1734
FALSE Extent.4_round_|_Population.Old.                  0.0234    *
FALSE InvAR.4_round_|_Population.Calf.                  0.5360
FALSE InvAR.4_round_|_Population.Old.                   0.8374
FALSE ConvexCircularity.4_round_|_Population.Calf.      0.2172
FALSE ConvexCircularity.4_round_|_Population.Old.       0.1474
FALSE Eccentricity.5_round_|_Population.Calf.           0.0180    *
FALSE Eccentricity.5_round_|_Population.Old.            0.0182    *
FALSE Solidity.5_round_|_Population.Calf.               0.0248    *
FALSE Solidity.5_round_|_Population.Old.                0.0118    *
FALSE Extent.5_round_|_Population.Calf.                 0.0130    *
FALSE Extent.5_round_|_Population.Old.                  0.0004  ***
```

FALSE	InvAR.5_round_|_Population.Calf.	0.0164	*
FALSE	InvAR.5_round_|_Population.Old.	0.0296	*
FALSE	ConvexCircularity.5_round_|_Population.Calf.	0.0438	*
FALSE	ConvexCircularity.5_round_|_Population.Old.	0.0008	***
FALSE	Eccentricity.6_round_|_Population.Calf.	0.2126	
FALSE	Eccentricity.6_round_|_Population.Old.	0.1056	
FALSE	Solidity.6_round_|_Population.Calf.	0.0546	
FALSE	Solidity.6_round_|_Population.Old.	0.0008	***
FALSE	Extent.6_round_|_Population.Calf.	0.1652	
FALSE	Extent.6_round_|_Population.Old.	0.0004	***
FALSE	InvAR.6_round_|_Population.Calf.	0.2006	
FALSE	InvAR.6_round_|_Population.Old.	0.0980	
FALSE	ConvexCircularity.6_round_|_Population.Calf.	0.0814	
FALSE	ConvexCircularity.6_round_|_Population.Old.	0.0012	**
FALSE	Eccentricity.1_pyramidal_|_Population.Calf.	0.0734	
FALSE	Eccentricity.1_pyramidal_|_Population.Old.	0.0008	***
FALSE	Solidity.1_pyramidal_|_Population.Calf.	0.3880	
FALSE	Solidity.1_pyramidal_|_Population.Old.	0.6384	
FALSE	Extent.1_pyramidal_|_Population.Calf.	0.4420	
FALSE	Extent.1_pyramidal_|_Population.Old.	0.0608	
FALSE	InvAR.1_pyramidal_|_Population.Calf.	0.0636	
FALSE	InvAR.1_pyramidal_|_Population.Old.	0.0008	***
FALSE	ConvexCircularity.1_pyramidal_|_Population.Calf.	0.0142	*
FALSE	ConvexCircularity.1_pyramidal_|_Population.Old.	0.0026	**
FALSE	Eccentricity.2_pyramidal_|_Population.Calf.	0.8846	
FALSE	Eccentricity.2_pyramidal_|_Population.Old.	0.5634	
FALSE	Solidity.2_pyramidal_|_Population.Calf.	0.0020	**
FALSE	Solidity.2_pyramidal_|_Population.Old.	0.5084	
FALSE	Extent.2_pyramidal_|_Population.Calf.	0.1754	
FALSE	Extent.2_pyramidal_|_Population.Old.	0.2916	
FALSE	InvAR.2_pyramidal_|_Population.Calf.	0.9256	
FALSE	InvAR.2_pyramidal_|_Population.Old.	0.5916	
FALSE	ConvexCircularity.2_pyramidal_|_Population.Calf.	0.5540	
FALSE	ConvexCircularity.2_pyramidal_|_Population.Old.	0.8594	
FALSE	Eccentricity.3_pyramidal_|_Population.Calf.	0.7324	
FALSE	Eccentricity.3_pyramidal_|_Population.Old.	0.1140	
FALSE	Solidity.3_pyramidal_|_Population.Calf.	0.0138	*
FALSE	Solidity.3_pyramidal_|_Population.Old.	0.7956	
FALSE	Extent.3_pyramidal_|_Population.Calf.	0.1570	
FALSE	Extent.3_pyramidal_|_Population.Old.	0.3170	
FALSE	InvAR.3_pyramidal_|_Population.Calf.	0.6390	
FALSE	InvAR.3_pyramidal_|_Population.Old.	0.1660	
FALSE	ConvexCircularity.3_pyramidal_|_Population.Calf.	0.4160	
FALSE	ConvexCircularity.3_pyramidal_|_Population.Old.	0.5696	
FALSE	Eccentricity.4_pyramidal_|_Population.Calf.	0.7360	
FALSE	Eccentricity.4_pyramidal_|_Population.Old.	0.5608	
FALSE	Solidity.4_pyramidal_|_Population.Calf.	0.7078	
FALSE	Solidity.4_pyramidal_|_Population.Old.	0.8818	
FALSE	Extent.4_pyramidal_|_Population.Calf.	0.4934	
FALSE	Extent.4_pyramidal_|_Population.Old.	0.6526	
FALSE	InvAR.4_pyramidal_|_Population.Calf.	0.7592	
FALSE	InvAR.4_pyramidal_|_Population.Old.	0.5382	
FALSE	ConvexCircularity.4_pyramidal_|_Population.Calf.	0.7642	
FALSE	ConvexCircularity.4_pyramidal_|_Population.Old.	0.5980	
FALSE	Eccentricity.5_pyramidal_|_Population.Calf.	0.2238	
FALSE	Eccentricity.5_pyramidal_|_Population.Old.	0.3228	
FALSE	Solidity.5_pyramidal_|_Population.Calf.	0.1402	
FALSE	Solidity.5_pyramidal_|_Population.Old.	0.7088	
FALSE	Extent.5_pyramidal_|_Population.Calf.	0.0858	
FALSE	Extent.5_pyramidal_|_Population.Old.	0.5702	
FALSE	InvAR.5_pyramidal_|_Population.Calf.	0.2384	
FALSE	InvAR.5_pyramidal_|_Population.Old.	0.3690	
FALSE	ConvexCircularity.5_pyramidal_|_Population.Calf.	0.4084	
FALSE	ConvexCircularity.5_pyramidal_|_Population.Old.	0.4762	
FALSE	Eccentricity.6_pyramidal_|_Population.Calf.	0.7152	
FALSE	Eccentricity.6_pyramidal_|_Population.Old.	0.1126	
FALSE	Solidity.6_pyramidal_|_Population.Calf.	0.0478	*
FALSE	Solidity.6_pyramidal_|_Population.Old.	0.6450	
FALSE	Extent.6_pyramidal_|_Population.Calf.	0.2718	
FALSE	Extent.6_pyramidal_|_Population.Old.	0.8612	
FALSE	InvAR.6_pyramidal_|_Population.Calf.	0.6116	
FALSE	InvAR.6_pyramidal_|_Population.Old.	0.1198	
FALSE	ConvexCircularity.6_pyramidal_|_Population.Calf.	0.5114	
FALSE	ConvexCircularity.6_pyramidal_|_Population.Old.	0.1224	
FALSE	Eccentricity.1_complex_|_Population.Calf.	0.3124	
FALSE	Eccentricity.1_complex_|_Population.Old.	0.5188	
FALSE	Solidity.1_complex_|_Population.Calf.	0.6638	
FALSE	Solidity.1_complex_|_Population.Old.	0.9934	

```
FALSE Extent.1_complex_|_Population.Calf.             0.2980
FALSE Extent.1_complex_|_Population.Old.              0.6196
FALSE InvAR.1_complex_|_Population.Calf.              0.2600
FALSE InvAR.1_complex_|_Population.Old.               0.5528
FALSE ConvexCircularity.1_complex_|_Population.Calf.    0.7860
FALSE ConvexCircularity.1_complex_|_Population.Old.     0.2862
FALSE Eccentricity.2_complex_|_Population.Calf.         0.0792
FALSE Eccentricity.2_complex_|_Population.Old.          0.6998
FALSE Solidity.2_complex_|_Population.Calf.           0.0026   **
FALSE Solidity.2_complex_|_Population.Old.            0.4928
FALSE Extent.2_complex_|_Population.Calf.             0.0124    *
FALSE Extent.2_complex_|_Population.Old.              0.5516
FALSE InvAR.2_complex_|_Population.Calf.              0.0794
FALSE InvAR.2_complex_|_Population.Old.               0.5794
FALSE ConvexCircularity.2_complex_|_Population.Calf.    0.0274    *
FALSE ConvexCircularity.2_complex_|_Population.Old.     0.6266
FALSE  [ reached 'max' / getOption("max.print") -- omitted 40 rows ]
```

Cobined by aspects

```
FALSE  Call:
FALSE  npc(permTP = res, subsets = ss_asp)
FALSE  permutations.
FALSE
FALSE                   comb.funct nVar   Stat p-value Adjust:maxT sig.
FALSE Eccentricity          Fisher   48  77.44  0.0084      0.0098   **
FALSE Solidity              Fisher   48 108.81  0.0098      0.0098   **
FALSE Extent                Fisher   48 118.40  0.0004      0.0060   **
FALSE InvAR                 Fisher   48  75.60  0.0106      0.0106    *
FALSE ConvexCircularity     Fisher   48  91.73  0.0012      0.0098   **
```

Cobined by Shape and Layer

```
FALSE  Call:
FALSE  npc(permTP = res, subsets = ids)
FALSE  permutations.
FALSE
FALSE             comb.funct nVar   Stat p-value Adjust:maxT sig.
FALSE ellipsoid_1     Fisher   10 14.596  0.1426      0.8028
FALSE ellipsoid_2     Fisher   10 10.709  0.3650      0.9216
FALSE ellipsoid_3     Fisher   10 11.109  0.3406      0.9216
FALSE ellipsoid_4     Fisher   10  4.264  0.9728      0.9960
FALSE ellipsoid_5     Fisher   10 27.005  0.0038      0.1110
FALSE ellipsoid_6     Fisher   10 17.839  0.0754      0.6144
FALSE round_1         Fisher   10 57.442  0.0002      0.0002  ***
FALSE round_2         Fisher   10 34.918  0.0036      0.0240    *
FALSE round_3         Fisher   10 33.513  0.0038      0.0294    *
FALSE round_4         Fisher   10 14.828  0.1490      0.8028
FALSE round_5         Fisher   10 46.217  0.0004      0.0012   **
FALSE round_6         Fisher   10 36.623  0.0014      0.0158    *
FALSE pyramidal_1     Fisher   10 34.848  0.0020      0.0240    *
FALSE pyramidal_2     Fisher   10 11.905  0.2842      0.9094
FALSE pyramidal_3     Fisher   10 13.679  0.1894      0.8314
FALSE pyramidal_4     Fisher   10  4.168  0.9158      0.9960
FALSE pyramidal_5     Fisher   10 12.022  0.2750      0.9094
FALSE pyramidal_6     Fisher   10 12.835  0.2310      0.8836
FALSE complex_1       Fisher   10  7.357  0.6998      0.9932
FALSE complex_2       Fisher   10 21.681  0.0502      0.3326
FALSE complex_3       Fisher   10 15.896  0.1474      0.7564
FALSE complex_4       Fisher   10  5.781  0.8086      0.9938
FALSE complex_5       Fisher   10  7.613  0.6620      0.9932
FALSE complex_6       Fisher   10 15.122  0.1132      0.7976
```

Cobined by Layer

```
FALSE  Call:
FALSE  npc(permTP = res, subsets = ss_Layers)
FALSE  permutations.
FALSE
FALSE   comb.funct nVar   Stat p-value Adjust:maxT sig.
FALSE 1     Fisher   40 114.24  0.0002      0.0004  ***
FALSE 2     Fisher   40  79.21  0.0160      0.0186    *
FALSE 3     Fisher   40  74.20  0.0096      0.0186    *
FALSE 4     Fisher   40  29.04  0.8854      0.8854
FALSE 5     Fisher   40  92.86  0.0010      0.0050   **
FALSE 6     Fisher   40  82.42  0.0028      0.0172    *
```

Pairwise comparisons

```
FALSE
FALSE         _-_-_-_-_-_-_-_-_-_- VARIABLE  Eccentricity.1_ellipsoid _-_-_-_-_-_-_-_-_-_-
FALSE
FALSE         _-_-_-_-_-_-_-_-_-_- Layer  1 _-_-_-_-_-_-_-_-_-_-
FALSE                        Raw (upper)
FALSE Adjusted (lower) Adult  Calf    Old
FALSE            Adult    NA 0.842 -0.881
FALSE            Calf      1    NA -0.797
FALSE            Old       1 1.000     NA
FALSE
FALSE         _-_-_-_-_-_-_-_-_-_- Layer  2 _-_-_-_-_-_-_-_-_-_-
FALSE                        Raw (upper)
FALSE Adjusted (lower) Adult   Calf   Old
FALSE            Adult    NA -0.458 0.623
FALSE            Calf      1     NA 0.385
FALSE            Old       1  1.000    NA
FALSE
FALSE         _-_-_-_-_-_-_-_-_-_- Layer  3 _-_-_-_-_-_-_-_-_-_-
FALSE                        Raw (upper)
FALSE Adjusted (lower) Adult   Calf    Old
FALSE            Adult    NA -0.204 -0.118
FALSE            Calf  0.353     NA -0.461
FALSE            Old   0.353  0.461     NA
FALSE
FALSE         _-_-_-_-_-_-_-_-_-_- Layer  4 _-_-_-_-_-_-_-_-_-_-
FALSE                        Raw (upper)
FALSE Adjusted (lower) Adult   Calf    Old
FALSE            Adult    NA -0.528 -0.234
FALSE            Calf  0.701     NA -0.855
FALSE            Old   0.701  0.855     NA
FALSE
FALSE         _-_-_-_-_-_-_-_-_-_- Layer  5 _-_-_-_-_-_-_-_-_-_-
FALSE                        Raw (upper)
FALSE Adjusted (lower) Adult   Calf    Old
FALSE            Adult    NA -0.025 -0.356
FALSE            Calf  0.074     NA  0.080
FALSE            Old   0.356  0.080     NA
FALSE
FALSE         _-_-_-_-_-_-_-_-_-_- Layer  6 _-_-_-_-_-_-_-_-_-_-
FALSE                        Raw (upper)
FALSE Adjusted (lower) Adult  Calf  Old
FALSE            Adult    NA 0.513 0.34
FALSE            Calf      1    NA 0.71
FALSE            Old       1 1.000   NA
FALSE
FALSE         _-_-_-_-_-_-_-_-_-_- VARIABLE  Solidity.1_ellipsoid _-_-_-_-_-_-_-_-_-_-
FALSE
FALSE         _-_-_-_-_-_-_-_-_-_- Layer  1 _-_-_-_-_-_-_-_-_-_-
FALSE                        Raw (upper)
FALSE Adjusted (lower) Adult  Calf    Old
FALSE            Adult    NA 0.637 -0.016
FALSE            Calf  0.637    NA -0.041
FALSE            Old   0.048 0.048     NA
FALSE
FALSE         _-_-_-_-_-_-_-_-_-_- Layer  2 _-_-_-_-_-_-_-_-_-_-
FALSE                        Raw (upper)
FALSE Adjusted (lower) Adult  Calf    Old
FALSE            Adult    NA 0.133 -0.725
FALSE            Calf  0.351    NA -0.117
FALSE            Old   0.725 0.351     NA
FALSE
FALSE         _-_-_-_-_-_-_-_-_-_- Layer  3 _-_-_-_-_-_-_-_-_-_-
FALSE                        Raw (upper)
FALSE Adjusted (lower) Adult  Calf    Old
FALSE            Adult    NA 0.000 -0.117
FALSE            Calf  0.000    NA -0.222
FALSE            Old   0.117 0.222     NA
FALSE
FALSE         _-_-_-_-_-_-_-_-_-_- Layer  4 _-_-_-_-_-_-_-_-_-_-
FALSE                        Raw (upper)
FALSE Adjusted (lower) Adult  Calf    Old
FALSE            Adult    NA -0.66 -0.975
FALSE            Calf      1    NA  0.633
FALSE            Old       1  1.00     NA
FALSE
FALSE         _-_-_-_-_-_-_-_-_-_- Layer  5 _-_-_-_-_-_-_-_-_-_-
FALSE                        Raw (upper)
FALSE Adjusted (lower) Adult  Calf    Old
```

```
FALSE           Adult    NA 0.326 -0.447
FALSE           Calf  0.326    NA -0.057
FALSE           Old   0.447 0.171     NA
FALSE
FALSE         _-_-_-_-_-_-_-_- Layer  6 _-_-_-_-_-_-_-_-
FALSE                         Raw (upper)
FALSE Adjusted (lower) Adult  Calf    Old
FALSE           Adult    NA 0.411 -0.107
FALSE           Calf  0.411    NA -0.021
FALSE           Old   0.107 0.064     NA
FALSE
FALSE         _-_-_-_-_-_-_-_- VARIABLE  Extent.1_ellipsoid _-_-_-_-_-_-_-_-
FALSE
FALSE         _-_-_-_-_-_-_-_- Layer  1 _-_-_-_-_-_-_-_-
FALSE                         Raw (upper)
FALSE Adjusted (lower) Adult  Calf    Old
FALSE           Adult    NA 0.128 -0.362
FALSE           Calf  0.239    NA -0.080
FALSE           Old   0.362 0.239     NA
FALSE
FALSE         _-_-_-_-_-_-_-_- Layer  2 _-_-_-_-_-_-_-_-
FALSE                         Raw (upper)
FALSE Adjusted (lower) Adult  Calf    Old
FALSE           Adult    NA 0.240  0.562
FALSE           Calf  0.721    NA -0.513
FALSE           Old   0.721 0.721     NA
FALSE
FALSE         _-_-_-_-_-_-_-_- Layer  3 _-_-_-_-_-_-_-_-
FALSE                         Raw (upper)
FALSE Adjusted (lower) Adult   Calf    Old
FALSE           Adult    NA -0.369 -0.022
FALSE           Calf  0.369     NA -0.348
FALSE           Old   0.065  0.348     NA
FALSE
FALSE         _-_-_-_-_-_-_-_- Layer  4 _-_-_-_-_-_-_-_-
FALSE                         Raw (upper)
FALSE Adjusted (lower) Adult   Calf    Old
FALSE           Adult    NA -0.376 -0.757
FALSE           Calf      1     NA  0.627
FALSE           Old       1  1.000     NA
FALSE
FALSE         _-_-_-_-_-_-_-_- Layer  5 _-_-_-_-_-_-_-_-
FALSE                         Raw (upper)
FALSE Adjusted (lower) Adult  Calf    Old
FALSE           Adult    NA 0.107 -0.087
FALSE           Calf  0.107    NA -0.009
FALSE           Old   0.087 0.028     NA
FALSE
FALSE         _-_-_-_-_-_-_-_- Layer  6 _-_-_-_-_-_-_-_-
FALSE                         Raw (upper)
FALSE Adjusted (lower) Adult   Calf    Old
FALSE           Adult    NA -0.929 -0.009
FALSE           Calf  0.929     NA -0.011
FALSE           Old   0.028  0.028     NA
FALSE
FALSE         _-_-_-_-_-_-_-_- VARIABLE  InvAR.1_ellipsoid _-_-_-_-_-_-_-_-
FALSE
FALSE         _-_-_-_-_-_-_-_- Layer  1 _-_-_-_-_-_-_-_-
FALSE                         Raw (upper)
FALSE Adjusted (lower) Adult   Calf   Old
FALSE           Adult    NA -0.854 0.928
FALSE           Calf      1     NA 0.847
FALSE           Old       1  1.000    NA
FALSE
FALSE         _-_-_-_-_-_-_-_- Layer  2 _-_-_-_-_-_-_-_-
FALSE                         Raw (upper)
FALSE Adjusted (lower) Adult  Calf    Old
FALSE           Adult    NA 0.394 -0.645
FALSE           Calf      1    NA -0.357
FALSE           Old       1 1.000     NA
FALSE
FALSE         _-_-_-_-_-_-_-_- Layer  3 _-_-_-_-_-_-_-_-
FALSE                         Raw (upper)
FALSE Adjusted (lower) Adult  Calf   Old
FALSE           Adult    NA 0.240 0.127
FALSE           Calf  0.382    NA 0.492
FALSE           Old   0.382 0.492    NA
FALSE
```

```
FALSE         _-_-_-_-_-_-_-_-_- Layer  4 _-_-_-_-_-_-_-_-_-
FALSE                          Raw (upper)
FALSE Adjusted (lower) Adult  Calf   Old
FALSE            Adult    NA 0.508 0.240
FALSE            Calf  0.719    NA 0.867
FALSE            Old   0.719 0.867    NA
FALSE
FALSE         _-_-_-_-_-_-_-_-_- Layer  5 _-_-_-_-_-_-_-_-_-
FALSE                          Raw (upper)
FALSE Adjusted (lower) Adult  Calf    Old
FALSE            Adult    NA 0.020  0.326
FALSE            Calf  0.059    NA -0.082
FALSE            Old   0.326 0.082     NA
FALSE
FALSE         _-_-_-_-_-_-_-_-_- Layer  6 _-_-_-_-_-_-_-_-_-
FALSE                          Raw (upper)
FALSE Adjusted (lower) Adult   Calf    Old
FALSE            Adult    NA -0.535 -0.342
FALSE            Calf      1     NA -0.697
FALSE            Old       1  1.000     NA
FALSE
FALSE         _-_-_-_-_-_-_-_-_- VARIABLE  ConvexCircularity.1_ellipsoid _-_-_-_-_-_-_-_-_-
FALSE
FALSE         _-_-_-_-_-_-_-_-_- Layer  1 _-_-_-_-_-_-_-_-_-
FALSE                          Raw (upper)
FALSE Adjusted (lower) Adult   Calf    Old
FALSE            Adult    NA -0.201 -0.227
FALSE            Calf  0.602     NA -0.756
FALSE            Old   0.602  0.756     NA
FALSE
FALSE         _-_-_-_-_-_-_-_-_- Layer  2 _-_-_-_-_-_-_-_-_-
FALSE                          Raw (upper)
FALSE Adjusted (lower) Adult   Calf    Old
FALSE            Adult    NA -0.636 -0.311
FALSE            Calf  0.934     NA -0.670
FALSE            Old   0.934  0.934     NA
FALSE
FALSE         _-_-_-_-_-_-_-_-_- Layer  3 _-_-_-_-_-_-_-_-_-
FALSE                          Raw (upper)
FALSE Adjusted (lower) Adult   Calf    Old
FALSE            Adult    NA -0.469 -0.519
FALSE            Calf      1     NA  0.931
FALSE            Old       1  1.000     NA
FALSE
FALSE         _-_-_-_-_-_-_-_-_- Layer  4 _-_-_-_-_-_-_-_-_-
FALSE                          Raw (upper)
FALSE Adjusted (lower) Adult   Calf    Old
FALSE            Adult    NA -0.707 -0.942
FALSE            Calf      1     NA  0.742
FALSE            Old       1  1.000     NA
FALSE
FALSE         _-_-_-_-_-_-_-_-_- Layer  5 _-_-_-_-_-_-_-_-_-
FALSE                          Raw (upper)
FALSE Adjusted (lower) Adult  Calf    Old
FALSE            Adult    NA 0.658 -0.091
FALSE            Calf  0.658    NA -0.078
FALSE            Old   0.235 0.235     NA
FALSE
FALSE         _-_-_-_-_-_-_-_-_- Layer  6 _-_-_-_-_-_-_-_-_-
FALSE                          Raw (upper)
FALSE Adjusted (lower) Adult   Calf    Old
FALSE            Adult    NA -0.384 -0.086
FALSE            Calf  0.384     NA -0.387
FALSE            Old   0.259  0.387     NA
FALSE
FALSE         _-_-_-_-_-_-_-_-_- VARIABLE  Eccentricity.1_round _-_-_-_-_-_-_-_-_-
FALSE
FALSE         _-_-_-_-_-_-_-_-_- Layer  1 _-_-_-_-_-_-_-_-_-
FALSE                          Raw (upper)
FALSE Adjusted (lower) Adult   Calf   Old
FALSE            Adult    NA -0.013 0.602
FALSE            Calf  0.013     NA 0.000
FALSE            Old   0.602  0.001    NA
FALSE
FALSE         _-_-_-_-_-_-_-_-_- Layer  2 _-_-_-_-_-_-_-_-_-
FALSE                          Raw (upper)
FALSE Adjusted (lower) Adult   Calf   Old
FALSE            Adult    NA -0.354 0.043
```

```
FALSE           Calf  0.354     NA 0.033
FALSE           Old   0.098  0.098    NA
FALSE
FALSE         _-_-_-_-_-_-_-_-_-_- Layer  3 _-_-_-_-_-_-_-_-_-_-
FALSE                          Raw (upper)
FALSE Adjusted (lower) Adult   Calf   Old
FALSE           Adult    NA -0.313 0.128
FALSE           Calf  0.313     NA 0.031
FALSE           Old   0.128  0.092    NA
FALSE
FALSE         _-_-_-_-_-_-_-_-_-_- Layer  4 _-_-_-_-_-_-_-_-_-_-
FALSE                          Raw (upper)
FALSE Adjusted (lower) Adult   Calf    Old
FALSE           Adult    NA -0.487 -0.603
FALSE           Calf      1     NA  0.726
FALSE           Old       1  1.000     NA
FALSE
FALSE         _-_-_-_-_-_-_-_-_-_- Layer  5 _-_-_-_-_-_-_-_-_-_-
FALSE                          Raw (upper)
FALSE Adjusted (lower) Adult   Calf   Old
FALSE           Adult    NA -0.162 0.079
FALSE           Calf  0.162     NA 0.011
FALSE           Old   0.079  0.032    NA
FALSE
FALSE         _-_-_-_-_-_-_-_-_-_- Layer  6 _-_-_-_-_-_-_-_-_-_-
FALSE                          Raw (upper)
FALSE Adjusted (lower) Adult   Calf   Old
FALSE           Adult    NA -0.586 0.185
FALSE           Calf  0.586     NA 0.037
FALSE           Old   0.185  0.112    NA
FALSE
FALSE         _-_-_-_-_-_-_-_-_-_- VARIABLE  Solidity.1_round _-_-_-_-_-_-_-_-_-_-
FALSE
FALSE         _-_-_-_-_-_-_-_-_-_- Layer  1 _-_-_-_-_-_-_-_-_-_-
FALSE                          Raw (upper)
FALSE Adjusted (lower) Adult  Calf    Old
FALSE           Adult    NA 0.033  0.000
FALSE           Calf  0.033    NA -0.002
FALSE           Old   0.001 0.002     NA
FALSE
FALSE         _-_-_-_-_-_-_-_-_-_- Layer  2 _-_-_-_-_-_-_-_-_-_-
FALSE                          Raw (upper)
FALSE Adjusted (lower) Adult  Calf    Old
FALSE           Adult    NA 0.041 -0.743
FALSE           Calf  0.123    NA -0.052
FALSE           Old   0.743 0.123     NA
FALSE
FALSE         _-_-_-_-_-_-_-_-_-_- Layer  3 _-_-_-_-_-_-_-_-_-_-
FALSE                          Raw (upper)
FALSE Adjusted (lower) Adult  Calf    Old
FALSE           Adult    NA 0.469 -0.120
FALSE           Calf  0.469    NA -0.087
FALSE           Old   0.260 0.260     NA
FALSE
FALSE         _-_-_-_-_-_-_-_-_-_- Layer  4 _-_-_-_-_-_-_-_-_-_-
FALSE                          Raw (upper)
FALSE Adjusted (lower) Adult  Calf    Old
FALSE           Adult    NA 0.639 -0.147
FALSE           Calf  0.639    NA -0.086
FALSE           Old   0.257 0.257     NA
FALSE
FALSE         _-_-_-_-_-_-_-_-_-_- Layer  5 _-_-_-_-_-_-_-_-_-_-
FALSE                          Raw (upper)
FALSE Adjusted (lower) Adult  Calf    Old
FALSE           Adult    NA 0.142 -0.069
FALSE           Calf  0.142    NA -0.010
FALSE           Old   0.069 0.029     NA
FALSE
FALSE         _-_-_-_-_-_-_-_-_-_- Layer  6 _-_-_-_-_-_-_-_-_-_-
FALSE                          Raw (upper)
FALSE Adjusted (lower) Adult  Calf    Old
FALSE           Adult    NA 0.359 -0.002
FALSE           Calf  0.359    NA -0.007
FALSE           Old   0.007 0.007     NA
FALSE
FALSE         _-_-_-_-_-_-_-_-_-_- VARIABLE  Extent.1_round _-_-_-_-_-_-_-_-_-_-
FALSE
FALSE         _-_-_-_-_-_-_-_-_-_- Layer  1 _-_-_-_-_-_-_-_-_-_-
```

```
FALSE                         Raw (upper)
FALSE Adjusted (lower) Adult  Calf    Old
FALSE            Adult    NA 0.011  0.000
FALSE            Calf  0.011    NA -0.002
FALSE            Old   0.001 0.002     NA
FALSE
FALSE         _-_-_-_-_-_-_-_-_- Layer  2 _-_-_-_-_-_-_-_-_-
FALSE                         Raw (upper)
FALSE Adjusted (lower) Adult  Calf    Old
FALSE            Adult    NA 0.019 -0.442
FALSE            Calf  0.046    NA -0.015
FALSE            Old   0.442 0.046     NA
FALSE
FALSE         _-_-_-_-_-_-_-_-_- Layer  3 _-_-_-_-_-_-_-_-_-
FALSE                         Raw (upper)
FALSE Adjusted (lower) Adult  Calf    Old
FALSE            Adult    NA 0.350 -0.012
FALSE            Calf  0.350    NA -0.011
FALSE            Old   0.033 0.033     NA
FALSE
FALSE         _-_-_-_-_-_-_-_-_- Layer  4 _-_-_-_-_-_-_-_-_-
FALSE                         Raw (upper)
FALSE Adjusted (lower) Adult  Calf    Old
FALSE            Adult    NA 0.556 -0.062
FALSE            Calf  0.556    NA -0.065
FALSE            Old   0.187 0.187     NA
FALSE
FALSE         _-_-_-_-_-_-_-_-_- Layer  5 _-_-_-_-_-_-_-_-_-
FALSE                         Raw (upper)
FALSE Adjusted (lower) Adult  Calf    Old
FALSE            Adult    NA 0.151 -0.001
FALSE            Calf  0.151    NA -0.002
FALSE            Old   0.002 0.002     NA
FALSE
FALSE         _-_-_-_-_-_-_-_-_- Layer  6 _-_-_-_-_-_-_-_-_-
FALSE                         Raw (upper)
FALSE Adjusted (lower) Adult   Calf    Old
FALSE            Adult    NA -0.944  0.000
FALSE            Calf  0.944     NA -0.003
FALSE            Old   0.001  0.003     NA
FALSE
FALSE         _-_-_-_-_-_-_-_-_- VARIABLE  InvAR.1_round _-_-_-_-_-_-_-_-_-
FALSE
FALSE         _-_-_-_-_-_-_-_-_- Layer  1 _-_-_-_-_-_-_-_-_-
FALSE                         Raw (upper)
FALSE Adjusted (lower) Adult  Calf    Old
FALSE            Adult    NA 0.005 -0.724
FALSE            Calf  0.005    NA -0.001
FALSE            Old   0.724 0.004     NA
FALSE
FALSE         _-_-_-_-_-_-_-_-_- Layer  2 _-_-_-_-_-_-_-_-_-
FALSE                         Raw (upper)
FALSE Adjusted (lower) Adult  Calf    Old
FALSE            Adult    NA 0.334 -0.067
FALSE            Calf  0.334    NA -0.040
FALSE            Old   0.120 0.120     NA
FALSE
FALSE         _-_-_-_-_-_-_-_-_- Layer  3 _-_-_-_-_-_-_-_-_-
FALSE                         Raw (upper)
FALSE Adjusted (lower) Adult  Calf    Old
FALSE            Adult    NA 0.377 -0.120
FALSE            Calf  0.377    NA -0.025
FALSE            Old   0.120 0.075     NA
FALSE
FALSE         _-_-_-_-_-_-_-_-_- Layer  4 _-_-_-_-_-_-_-_-_-
FALSE                         Raw (upper)
FALSE Adjusted (lower) Adult  Calf    Old
FALSE            Adult    NA 0.499  0.604
FALSE            Calf      1    NA -0.763
FALSE            Old       1 1.000     NA
FALSE
FALSE         _-_-_-_-_-_-_-_-_- Layer  5 _-_-_-_-_-_-_-_-_-
FALSE                         Raw (upper)
FALSE Adjusted (lower) Adult  Calf    Old
FALSE            Adult    NA 0.135 -0.123
FALSE            Calf  0.135    NA -0.018
FALSE            Old   0.123 0.055     NA
FALSE
```

```
FALSE         _-_-_-_-_-_-_-_-_- Layer  6 _-_-_-_-_-_-_-_-_-
FALSE                          Raw (upper)
FALSE Adjusted (lower) Adult  Calf    Old
FALSE             Adult    NA 0.605 -0.205
FALSE             Calf  0.605    NA -0.029
FALSE             Old   0.205 0.087     NA
FALSE
FALSE         _-_-_-_-_-_-_-_-_- VARIABLE  ConvexCircularity.1_round _-_-_-_-_-_-_-_-_-
FALSE
FALSE         _-_-_-_-_-_-_-_-_- Layer  1 _-_-_-_-_-_-_-_-_-
FALSE                          Raw (upper)
FALSE Adjusted (lower) Adult  Calf    Old
FALSE             Adult    NA 0.010 -0.008
FALSE             Calf  0.010    NA -0.001
FALSE             Old   0.008 0.004     NA
FALSE
FALSE         _-_-_-_-_-_-_-_-_- Layer  2 _-_-_-_-_-_-_-_-_-
FALSE                          Raw (upper)
FALSE Adjusted (lower) Adult  Calf    Old
FALSE             Adult    NA 0.056 -0.116
FALSE             Calf  0.056    NA -0.013
FALSE             Old   0.116 0.038     NA
FALSE
FALSE         _-_-_-_-_-_-_-_-_- Layer  3 _-_-_-_-_-_-_-_-_-
FALSE                          Raw (upper)
FALSE Adjusted (lower) Adult  Calf    Old
FALSE             Adult    NA 0.454 -0.039
FALSE             Calf  0.454    NA -0.010
FALSE             Old   0.039 0.031     NA
FALSE
FALSE         _-_-_-_-_-_-_-_-_- Layer  4 _-_-_-_-_-_-_-_-_-
FALSE                          Raw (upper)
FALSE Adjusted (lower) Adult  Calf    Old
FALSE             Adult    NA 0.490 -0.317
FALSE             Calf  0.490    NA -0.151
FALSE             Old   0.454 0.454     NA
FALSE
FALSE         _-_-_-_-_-_-_-_-_- Layer  5 _-_-_-_-_-_-_-_-_-
FALSE                          Raw (upper)
FALSE Adjusted (lower) Adult  Calf    Old
FALSE             Adult    NA 0.296 -0.015
FALSE             Calf  0.296    NA -0.006
FALSE             Old   0.017 0.017     NA
FALSE
FALSE         _-_-_-_-_-_-_-_-_- Layer  6 _-_-_-_-_-_-_-_-_-
FALSE                          Raw (upper)
FALSE Adjusted (lower) Adult  Calf    Old
FALSE             Adult    NA 0.475 -0.009
FALSE             Calf  0.475    NA -0.008
FALSE             Old   0.024 0.024     NA
FALSE
FALSE         _-_-_-_-_-_-_-_-_- VARIABLE  Eccentricity.1_pyramidal _-_-_-_-_-_-_-_-_-
FALSE
FALSE         _-_-_-_-_-_-_-_-_- Layer  1 _-_-_-_-_-_-_-_-_-
FALSE                          Raw (upper)
FALSE Adjusted (lower) Adult  Calf   Old
FALSE             Adult    NA 0.448  0.00
FALSE             Calf  0.448    NA -0.01
FALSE             Old   0.001 0.010    NA
FALSE
FALSE         _-_-_-_-_-_-_-_-_- Layer  2 _-_-_-_-_-_-_-_-_-
FALSE                          Raw (upper)
FALSE Adjusted (lower) Adult   Calf    Old
FALSE             Adult    NA -0.908 -0.573
FALSE             Calf      1     NA -0.695
FALSE             Old       1  1.000     NA
FALSE
FALSE         _-_-_-_-_-_-_-_-_- Layer  3 _-_-_-_-_-_-_-_-_-
FALSE                          Raw (upper)
FALSE Adjusted (lower) Adult   Calf    Old
FALSE             Adult    NA -0.254 -0.094
FALSE             Calf  0.281     NA -0.386
FALSE             Old   0.281  0.386     NA
FALSE
FALSE         _-_-_-_-_-_-_-_-_- Layer  4 _-_-_-_-_-_-_-_-_-
FALSE                          Raw (upper)
FALSE Adjusted (lower) Adult   Calf    Old
FALSE             Adult    NA -0.517 -0.395
```

```
FALSE           Calf      1     NA -0.938
FALSE           Old       1  1.000     NA
FALSE
FALSE        _-_-_-_-_-_-_-_- Layer  5 _-_-_-_-_-_-_-_-
FALSE                        Raw (upper)
FALSE Adjusted (lower) Adult   Calf    Old
FALSE           Adult    NA -0.076 -0.071
FALSE           Calf  0.213     NA  0.833
FALSE           Old   0.213  0.833     NA
FALSE
FALSE        _-_-_-_-_-_-_-_- Layer  6 _-_-_-_-_-_-_-_-
FALSE                        Raw (upper)
FALSE Adjusted (lower) Adult   Calf    Old
FALSE           Adult    NA -0.209 -0.081
FALSE           Calf  0.243     NA -0.451
FALSE           Old   0.243  0.451     NA
FALSE
FALSE        _-_-_-_-_-_-_-_- VARIABLE  Solidity.1_pyramidal _-_-_-_-_-_-_-_-
FALSE
FALSE        _-_-_-_-_-_-_-_- Layer  1 _-_-_-_-_-_-_-_-
FALSE                        Raw (upper)
FALSE Adjusted (lower) Adult  Calf    Old
FALSE           Adult    NA 0.256  0.337
FALSE           Calf  0.768    NA -0.733
FALSE           Old   0.768 0.768     NA
FALSE
FALSE        _-_-_-_-_-_-_-_- Layer  2 _-_-_-_-_-_-_-_-
FALSE                        Raw (upper)
FALSE Adjusted (lower) Adult  Calf    Old
FALSE           Adult    NA 0.006  0.317
FALSE           Calf  0.018    NA -0.019
FALSE           Old   0.317 0.019     NA
FALSE
FALSE        _-_-_-_-_-_-_-_- Layer  3 _-_-_-_-_-_-_-_-
FALSE                        Raw (upper)
FALSE Adjusted (lower) Adult  Calf    Old
FALSE           Adult    NA 0.014  0.292
FALSE           Calf  0.041    NA -0.080
FALSE           Old   0.292 0.080     NA
FALSE
FALSE        _-_-_-_-_-_-_-_- Layer  4 _-_-_-_-_-_-_-_-
FALSE                        Raw (upper)
FALSE Adjusted (lower) Adult  Calf    Old
FALSE           Adult    NA 0.764  0.990
FALSE           Calf      1    NA -0.707
FALSE           Old       1 1.000     NA
FALSE
FALSE        _-_-_-_-_-_-_-_- Layer  5 _-_-_-_-_-_-_-_-
FALSE                        Raw (upper)
FALSE Adjusted (lower) Adult  Calf    Old
FALSE           Adult    NA 0.100  0.190
FALSE           Calf  0.299    NA -0.499
FALSE           Old   0.299 0.499     NA
FALSE
FALSE        _-_-_-_-_-_-_-_- Layer  6 _-_-_-_-_-_-_-_-
FALSE                        Raw (upper)
FALSE Adjusted (lower) Adult  Calf    Old
FALSE           Adult    NA 0.090  0.584
FALSE           Calf  0.271    NA -0.198
FALSE           Old   0.584 0.271     NA
FALSE
FALSE        _-_-_-_-_-_-_-_- VARIABLE  Extent.1_pyramidal _-_-_-_-_-_-_-_-
FALSE
FALSE        _-_-_-_-_-_-_-_- Layer  1 _-_-_-_-_-_-_-_-
FALSE                        Raw (upper)
FALSE Adjusted (lower) Adult  Calf   Old
FALSE           Adult    NA 0.988 0.076
FALSE           Calf  0.988    NA 0.164
FALSE           Old   0.227 0.227    NA
FALSE
FALSE        _-_-_-_-_-_-_-_- Layer  2 _-_-_-_-_-_-_-_-
FALSE                        Raw (upper)
FALSE Adjusted (lower) Adult  Calf    Old
FALSE           Adult    NA 0.053  0.018
FALSE           Calf  0.054    NA -0.781
FALSE           Old   0.054 0.781     NA
FALSE
FALSE        _-_-_-_-_-_-_-_- Layer  3 _-_-_-_-_-_-_-_-
```

```
FALSE                          Raw (upper)
FALSE Adjusted (lower) Adult  Calf    Old
FALSE            Adult    NA 0.041  0.011
FALSE            Calf  0.041    NA -0.734
FALSE            Old   0.032 0.734     NA
FALSE
FALSE         _-_-_-_-_-_-_-_-_- Layer  4 _-_-_-_-_-_-_-_-_-
FALSE                          Raw (upper)
FALSE Adjusted (lower) Adult  Calf    Old
FALSE            Adult    NA 0.415  0.348
FALSE            Calf      1    NA -0.899
FALSE            Old       1 1.000     NA
FALSE
FALSE         _-_-_-_-_-_-_-_-_- Layer  5 _-_-_-_-_-_-_-_-_-
FALSE                          Raw (upper)
FALSE Adjusted (lower) Adult  Calf    Old
FALSE            Adult    NA 0.032  0.025
FALSE            Calf  0.075    NA -0.632
FALSE            Old   0.075 0.632     NA
FALSE
FALSE         _-_-_-_-_-_-_-_-_- Layer  6 _-_-_-_-_-_-_-_-_-
FALSE                          Raw (upper)
FALSE Adjusted (lower) Adult  Calf    Old
FALSE            Adult    NA 0.362  0.707
FALSE            Calf      1    NA -0.503
FALSE            Old       1 1.000     NA
FALSE
FALSE         _-_-_-_-_-_-_-_-_- VARIABLE  InvAR.1_pyramidal _-_-_-_-_-_-_-_-_-
FALSE
FALSE         _-_-_-_-_-_-_-_-_- Layer  1 _-_-_-_-_-_-_-_-_-
FALSE                          Raw (upper)
FALSE Adjusted (lower) Adult   Calf   Old
FALSE            Adult    NA -0.384 0.001
FALSE            Calf  0.384     NA 0.009
FALSE            Old   0.004  0.009    NA
FALSE
FALSE         _-_-_-_-_-_-_-_-_- Layer  2 _-_-_-_-_-_-_-_-_-
FALSE                          Raw (upper)
FALSE Adjusted (lower) Adult Calf   Old
FALSE            Adult    NA 0.71 0.541
FALSE            Calf      1   NA 0.823
FALSE            Old       1 1.00    NA
FALSE
FALSE         _-_-_-_-_-_-_-_-_- Layer  3 _-_-_-_-_-_-_-_-_-
FALSE                          Raw (upper)
FALSE Adjusted (lower) Adult  Calf   Old
FALSE            Adult    NA 0.223 0.118
FALSE            Calf  0.355    NA 0.559
FALSE            Old   0.355 0.559    NA
FALSE
FALSE         _-_-_-_-_-_-_-_-_- Layer  4 _-_-_-_-_-_-_-_-_-
FALSE                          Raw (upper)
FALSE Adjusted (lower) Adult  Calf   Old
FALSE            Adult    NA 0.525 0.403
FALSE            Calf      1    NA 0.901
FALSE            Old       1 1.000    NA
FALSE
FALSE         _-_-_-_-_-_-_-_-_- Layer  5 _-_-_-_-_-_-_-_-_-
FALSE                          Raw (upper)
FALSE Adjusted (lower) Adult  Calf    Old
FALSE            Adult    NA 0.115  0.096
FALSE            Calf  0.289    NA -0.810
FALSE            Old   0.289 0.810     NA
FALSE
FALSE         _-_-_-_-_-_-_-_-_- Layer  6 _-_-_-_-_-_-_-_-_-
FALSE                          Raw (upper)
FALSE Adjusted (lower) Adult  Calf   Old
FALSE            Adult    NA 0.164 0.078
FALSE            Calf  0.235    NA 0.498
FALSE            Old   0.235 0.498    NA
FALSE
FALSE         _-_-_-_-_-_-_-_-_- VARIABLE  ConvexCircularity.1_pyramidal _-_-_-_-_-_-_-_-_-
FALSE
FALSE         _-_-_-_-_-_-_-_-_- Layer  1 _-_-_-_-_-_-_-_-_-
FALSE                          Raw (upper)
FALSE Adjusted (lower) Adult   Calf   Old
FALSE            Adult    NA -0.127 0.007
FALSE            Calf  0.127     NA 0.008
```

```
FALSE           Old   0.022  0.022    NA
FALSE
FALSE         _-_-_-_-_-_-_-_-_-_- Layer  2 _-_-_-_-_-_-_-_-_-_-
FALSE                          Raw (upper)
FALSE Adjusted (lower) Adult  Calf    Old
FALSE           Adult    NA 0.458  0.617
FALSE           Calf      1    NA -0.797
FALSE           Old       1 1.000     NA
FALSE
FALSE         _-_-_-_-_-_-_-_-_-_- Layer  3 _-_-_-_-_-_-_-_-_-_-
FALSE                          Raw (upper)
FALSE Adjusted (lower) Adult  Calf    Old
FALSE           Adult    NA 0.209  0.331
FALSE           Calf  0.628    NA -0.811
FALSE           Old   0.628 0.811     NA
FALSE
FALSE         _-_-_-_-_-_-_-_-_-_- Layer  4 _-_-_-_-_-_-_-_-_-_-
FALSE                          Raw (upper)
FALSE Adjusted (lower) Adult Calf   Old
FALSE           Adult    NA 0.57 0.465
FALSE           Calf      1   NA 0.890
FALSE           Old       1 1.00    NA
FALSE
FALSE         _-_-_-_-_-_-_-_-_-_- Layer  5 _-_-_-_-_-_-_-_-_-_-
FALSE                          Raw (upper)
FALSE Adjusted (lower) Adult  Calf    Old
FALSE           Adult    NA 0.303  0.166
FALSE           Calf  0.499    NA -0.930
FALSE           Old   0.499 0.930     NA
FALSE
FALSE         _-_-_-_-_-_-_-_-_-_- Layer  6 _-_-_-_-_-_-_-_-_-_-
FALSE                          Raw (upper)
FALSE Adjusted (lower) Adult  Calf   Old
FALSE           Adult    NA 0.157 0.068
FALSE           Calf  0.203    NA 0.546
FALSE           Old   0.203 0.546    NA
FALSE
FALSE         _-_-_-_-_-_-_-_-_-_- VARIABLE  Eccentricity.1_complex _-_-_-_-_-_-_-_-_-_-
FALSE
FALSE         _-_-_-_-_-_-_-_-_-_- Layer  1 _-_-_-_-_-_-_-_-_-_-
FALSE                          Raw (upper)
FALSE Adjusted (lower) Adult   Calf   Old
FALSE           Adult    NA -0.256 0.867
FALSE           Calf  0.769     NA 0.412
FALSE           Old   0.867  0.769    NA
FALSE
FALSE         _-_-_-_-_-_-_-_-_-_- Layer  2 _-_-_-_-_-_-_-_-_-_-
FALSE                          Raw (upper)
FALSE Adjusted (lower) Adult  Calf    Old
FALSE           Adult    NA 0.132  0.637
FALSE           Calf  0.395    NA -0.192
FALSE           Old   0.637 0.395     NA
FALSE
FALSE         _-_-_-_-_-_-_-_-_-_- Layer  3 _-_-_-_-_-_-_-_-_-_-
FALSE                          Raw (upper)
FALSE Adjusted (lower) Adult Calf    Old
FALSE           Adult    NA 0.19  0.143
FALSE           Calf  0.428   NA -0.740
FALSE           Old   0.428 0.74     NA
FALSE
FALSE         _-_-_-_-_-_-_-_-_-_- Layer  4 _-_-_-_-_-_-_-_-_-_-
FALSE                          Raw (upper)
FALSE Adjusted (lower) Adult   Calf    Old
FALSE           Adult    NA -0.721 -0.772
FALSE           Calf      1     NA  0.904
FALSE           Old       1  1.000     NA
FALSE
FALSE         _-_-_-_-_-_-_-_-_-_- Layer  5 _-_-_-_-_-_-_-_-_-_-
FALSE                          Raw (upper)
FALSE Adjusted (lower) Adult  Calf    Old
FALSE           Adult    NA 0.653 -0.957
FALSE           Calf      1    NA -0.751
FALSE           Old       1 1.000     NA
FALSE
FALSE         _-_-_-_-_-_-_-_-_-_- Layer  6 _-_-_-_-_-_-_-_-_-_-
FALSE                          Raw (upper)
FALSE Adjusted (lower) Adult   Calf   Old
FALSE           Adult    NA -0.253 0.429
```

```
FALSE           Calf  0.253     NA 0.047
FALSE           Old   0.429  0.142    NA
FALSE
FALSE         _-_-_-_-_-_-_-_-_-_- VARIABLE  Solidity.1_complex _-_-_-_-_-_-_-_-_-_-
FALSE
FALSE         _-_-_-_-_-_-_-_-_-_- Layer  1 _-_-_-_-_-_-_-_-_-_-
FALSE                        Raw (upper)
FALSE Adjusted (lower) Adult  Calf    Old
FALSE           Adult    NA 0.663  0.754
FALSE           Calf      1    NA -0.825
FALSE           Old       1 1.000     NA
FALSE
FALSE         _-_-_-_-_-_-_-_-_-_- Layer  2 _-_-_-_-_-_-_-_-_-_-
FALSE                        Raw (upper)
FALSE Adjusted (lower) Adult  Calf    Old
FALSE           Adult    NA 0.004  0.417
FALSE           Calf  0.013    NA -0.032
FALSE           Old   0.417 0.032     NA
FALSE
FALSE         _-_-_-_-_-_-_-_-_-_- Layer  3 _-_-_-_-_-_-_-_-_-_-
FALSE                        Raw (upper)
FALSE Adjusted (lower) Adult  Calf    Old
FALSE           Adult    NA 0.023  0.044
FALSE           Calf  0.068    NA -0.408
FALSE           Old   0.068 0.408     NA
FALSE
FALSE         _-_-_-_-_-_-_-_-_-_- Layer  4 _-_-_-_-_-_-_-_-_-_-
FALSE                        Raw (upper)
FALSE Adjusted (lower) Adult  Calf    Old
FALSE           Adult    NA 0.195  0.903
FALSE           Calf  0.240    NA -0.080
FALSE           Old   0.903 0.240     NA
FALSE
FALSE         _-_-_-_-_-_-_-_-_-_- Layer  5 _-_-_-_-_-_-_-_-_-_-
FALSE                        Raw (upper)
FALSE Adjusted (lower) Adult  Calf    Old
FALSE           Adult    NA 0.121  0.364
FALSE           Calf  0.362    NA -0.348
FALSE           Old   0.364 0.362     NA
FALSE
FALSE         _-_-_-_-_-_-_-_-_-_- Layer  6 _-_-_-_-_-_-_-_-_-_-
FALSE                        Raw (upper)
FALSE Adjusted (lower) Adult  Calf    Old
FALSE           Adult    NA 0.094  0.841
FALSE           Calf  0.282    NA -0.195
FALSE           Old   0.841 0.282     NA
FALSE
FALSE         _-_-_-_-_-_-_-_-_-_- VARIABLE  Extent.1_complex _-_-_-_-_-_-_-_-_-_-
FALSE
FALSE         _-_-_-_-_-_-_-_-_-_- Layer  1 _-_-_-_-_-_-_-_-_-_-
FALSE                        Raw (upper)
FALSE Adjusted (lower) Adult Calf    Old
FALSE           Adult    NA 0.44 -0.932
FALSE           Calf      1   NA -0.384
FALSE           Old       1 1.00     NA
FALSE
FALSE         _-_-_-_-_-_-_-_-_-_- Layer  2 _-_-_-_-_-_-_-_-_-_-
FALSE                        Raw (upper)
FALSE Adjusted (lower) Adult  Calf    Old
FALSE           Adult    NA 0.013  0.556
FALSE           Calf  0.038    NA -0.082
FALSE           Old   0.556 0.082     NA
FALSE
FALSE         _-_-_-_-_-_-_-_-_-_- Layer  3 _-_-_-_-_-_-_-_-_-_-
FALSE                        Raw (upper)
FALSE Adjusted (lower) Adult  Calf    Old
FALSE           Adult    NA 0.007  0.121
FALSE           Calf  0.022    NA -0.142
FALSE           Old   0.121 0.142     NA
FALSE
FALSE         _-_-_-_-_-_-_-_-_-_- Layer  4 _-_-_-_-_-_-_-_-_-_-
FALSE                        Raw (upper)
FALSE Adjusted (lower) Adult  Calf    Old
FALSE           Adult    NA 0.540 -0.732
FALSE           Calf  0.892    NA -0.297
FALSE           Old   0.892 0.892     NA
FALSE
FALSE         _-_-_-_-_-_-_-_-_-_- Layer  5 _-_-_-_-_-_-_-_-_-_-
```

```
FALSE                          Raw (upper)
FALSE Adjusted (lower) Adult  Calf    Old
FALSE            Adult    NA 0.116 -0.969
FALSE            Calf  0.186    NA -0.062
FALSE            Old   0.969 0.186     NA
FALSE
FALSE         _-_-_-_-_-_-_-_-_- Layer  6 _-_-_-_-_-_-_-_-_-
FALSE                          Raw (upper)
FALSE Adjusted (lower) Adult  Calf    Old
FALSE            Adult    NA 0.221 -0.848
FALSE            Calf  0.492    NA -0.164
FALSE            Old   0.848 0.492     NA
FALSE
FALSE         _-_-_-_-_-_-_-_-_- VARIABLE  InvAR.1_complex _-_-_-_-_-_-_-_-_-
FALSE
FALSE         _-_-_-_-_-_-_-_-_- Layer  1 _-_-_-_-_-_-_-_-_-
FALSE                          Raw (upper)
FALSE Adjusted (lower) Adult  Calf    Old
FALSE            Adult    NA 0.202 -0.935
FALSE            Calf  0.607    NA -0.391
FALSE            Old   0.935 0.607     NA
FALSE
FALSE         _-_-_-_-_-_-_-_-_- Layer  2 _-_-_-_-_-_-_-_-_-
FALSE                          Raw (upper)
FALSE Adjusted (lower) Adult   Calf    Old
FALSE            Adult    NA -0.157 -0.803
FALSE            Calf  0.462     NA  0.154
FALSE            Old   0.803  0.462     NA
FALSE
FALSE         _-_-_-_-_-_-_-_-_- Layer  3 _-_-_-_-_-_-_-_-_-
FALSE                          Raw (upper)
FALSE Adjusted (lower) Adult   Calf    Old
FALSE            Adult    NA -0.140 -0.162
FALSE            Calf  0.421     NA  0.611
FALSE            Old   0.421  0.611     NA
FALSE
FALSE         _-_-_-_-_-_-_-_-_- Layer  4 _-_-_-_-_-_-_-_-_-
FALSE                          Raw (upper)
FALSE Adjusted (lower) Adult  Calf   Old
FALSE            Adult    NA 0.944 0.928
FALSE            Calf      1    NA 0.990
FALSE            Old       1 1.000    NA
FALSE
FALSE         _-_-_-_-_-_-_-_-_- Layer  5 _-_-_-_-_-_-_-_-_-
FALSE                          Raw (upper)
FALSE Adjusted (lower) Adult   Calf   Old
FALSE            Adult    NA -0.693 0.881
FALSE            Calf      1     NA 0.728
FALSE            Old       1  1.000    NA
FALSE
FALSE         _-_-_-_-_-_-_-_-_- Layer  6 _-_-_-_-_-_-_-_-_-
FALSE                          Raw (upper)
FALSE Adjusted (lower) Adult Calf    Old
FALSE            Adult    NA 0.22 -0.613
FALSE            Calf  0.390   NA -0.130
FALSE            Old   0.613 0.39     NA
FALSE
FALSE         _-_-_-_-_-_-_-_-_- VARIABLE  ConvexCircularity.1_complex _-_-_-_-_-_-_-_-_-
FALSE
FALSE         _-_-_-_-_-_-_-_-_- Layer  1 _-_-_-_-_-_-_-_-_-
FALSE                          Raw (upper)
FALSE Adjusted (lower) Adult  Calf   Old
FALSE            Adult    NA 0.853 0.194
FALSE            Calf  0.853    NA 0.505
FALSE            Old   0.583 0.583    NA
FALSE
FALSE         _-_-_-_-_-_-_-_-_- Layer  2 _-_-_-_-_-_-_-_-_-
FALSE                          Raw (upper)
FALSE Adjusted (lower) Adult  Calf    Old
FALSE            Adult    NA 0.046  0.606
FALSE            Calf  0.130    NA -0.043
FALSE            Old   0.606 0.130     NA
FALSE
FALSE         _-_-_-_-_-_-_-_-_- Layer  3 _-_-_-_-_-_-_-_-_-
FALSE                          Raw (upper)
FALSE Adjusted (lower) Adult  Calf    Old
FALSE            Adult    NA 0.006  0.097
FALSE            Calf  0.017    NA -0.258
```

```
FALSE           Old   0.097 0.258     NA
FALSE
FALSE        _-_-_-_-_-_-_-_- Layer  4 _-_-_-_-_-_-_-_-
FALSE                         Raw (upper)
FALSE Adjusted (lower) Adult  Calf    Old
FALSE           Adult    NA 0.521  0.902
FALSE           Calf      1    NA -0.450
FALSE           Old       1 1.000     NA
FALSE
FALSE        _-_-_-_-_-_-_-_- Layer  5 _-_-_-_-_-_-_-_-
FALSE                         Raw (upper)
FALSE Adjusted (lower) Adult Calf Old
FALSE           Adult    NA    0   0
FALSE           Calf      0   NA   0
FALSE           Old       0    0  NA
FALSE
FALSE        _-_-_-_-_-_-_-_- Layer  6 _-_-_-_-_-_-_-_-
FALSE                         Raw (upper)
FALSE Adjusted (lower) Adult   Calf   Old
FALSE           Adult    NA -0.849 0.903
FALSE           Calf      1     NA 0.800
FALSE           Old       1  1.000    NA
```
