# Supplementary material for: Age-related changes in the primary auditory cortex of newborn, adults and aging bottlenose dolphins (Tursiops truncatus) are located in the upper cortical layers
Source: Front Neuroanat. 2024 Jan 5;17:1330384. doi: 10.3389/fnana.2023.1330384 (PMC10796513; doi:10.3389/fnana.2023.1330384)
Supplement: Supplementary file 3 [file Data_Sheet_3.pdf]

Analysis Density

- Descriprives
  - means by subject
    - Density50
    - Density100
  - Radar Plot
  - Density
  - Principal Components
    - Layer 1
    - Layer 2
    - Layer 3
    - Layer 4
    - Layer 5
    - Layer 6
- Inferential Analysis
  - Univariate
  - Cobined by aspects
  - Cobined by Shape and Layer
  - Cobined by Layer
  - Pairwise comparisons

Descriprives

means by subject

Density 50

Population	Shape	Layer_ID	mean	sd
Adult	ellipsoid	1	5.42	0.80
Calf	ellipsoid	1	5.65	2.52
Old	ellipsoid	1	3.60	1.46
Adult	ellipsoid	2	9.00	1.39
Calf	ellipsoid	2	11.95	3.12
Old	ellipsoid	2	7.41	1.59
Adult	ellipsoid	3	8.53	0.52
Calf	ellipsoid	3	11.20	2.96
Old	ellipsoid	3	6.70	1.07
Adult	ellipsoid	4	2.96	0.23
Calf	ellipsoid	4	3.53	1.02
Old	ellipsoid	4	2.84	0.95
Adult	ellipsoid	5	7.68	0.84
Calf	ellipsoid	5	10.00	2.73
Old	ellipsoid	5	5.70	1.26
Adult	ellipsoid	6	7.49	0.97
Calf	ellipsoid	6	9.81	2.51
Old	ellipsoid	6	5.44	1.65
Adult	round	1	5.44	0.82
Calf	round	1	5.56	2.59
Old	round	1	3.53	1.47
Adult	round	2	8.86	1.25
Calf	round	2	11.53	3.07
Old	round	2	7.31	1.65

Population	Shape	Layer_ID	mean	sd
Adult	round	3	8.51	0.54
Calf	round	3	11.04	2.84
Old	round	3	6.57	1.13
Adult	round	4	2.99	0.37
Calf	round	4	3.33	1.02
Old	round	4	2.92	0.94
Adult	round	5	7.62	0.81
Calf	round	5	9.83	2.52
Old	round	5	5.59	1.22
Adult	round	6	7.48	0.84
Calf	round	6	9.50	2.46
Old	round	6	5.48	1.66
Adult	pyramidal	1	5.42	0.91
Calf	pyramidal	1	5.77	2.47
Old	pyramidal	1	3.63	1.39
Adult	pyramidal	2	9.03	0.97
Calf	pyramidal	2	11.70	2.96
Old	pyramidal	2	7.46	1.73
Adult	pyramidal	3	8.67	0.52
Calf	pyramidal	3	11.32	3.05
Old	pyramidal	3	6.70	1.14
Adult	pyramidal	4	3.08	0.36
Calf	pyramidal	4	3.53	1.16
Old	pyramidal	4	2.97	1.00
Adult	pyramidal	5	7.98	0.79
Calf	pyramidal	5	10.14	2.35
Old	pyramidal	5	5.89	1.16
Adult	pyramidal	6	7.87	0.85
Calf	pyramidal	6	10.08	2.51
Old	pyramidal	6	5.70	1.59
Adult	complex	1	5.32	1.03
Calf	complex	1	5.67	2.66
Old	complex	1	3.63	1.42
Adult	complex	2	9.26	1.01
Calf	complex	2	11.89	3.12
Old	complex	2	7.54	1.77
Adult	complex	3	8.94	0.51
Calf	complex	3	11.30	2.97
Old	complex	3	6.78	1.22
Adult	complex	4	3.19	0.45
Calf	complex	4	3.70	1.24
Old	complex	4	3.02	1.01
Adult	complex	5	8.06	1.02
Calf	complex	5	10.28	2.62
Old	complex	5	5.87	1.29
Adult	complex	6	8.02	0.93

Population	Shape	Layer_ID	mean	sd
Calf	complex	6	10.05	2.57
Old	complex	6	5.78	1.72

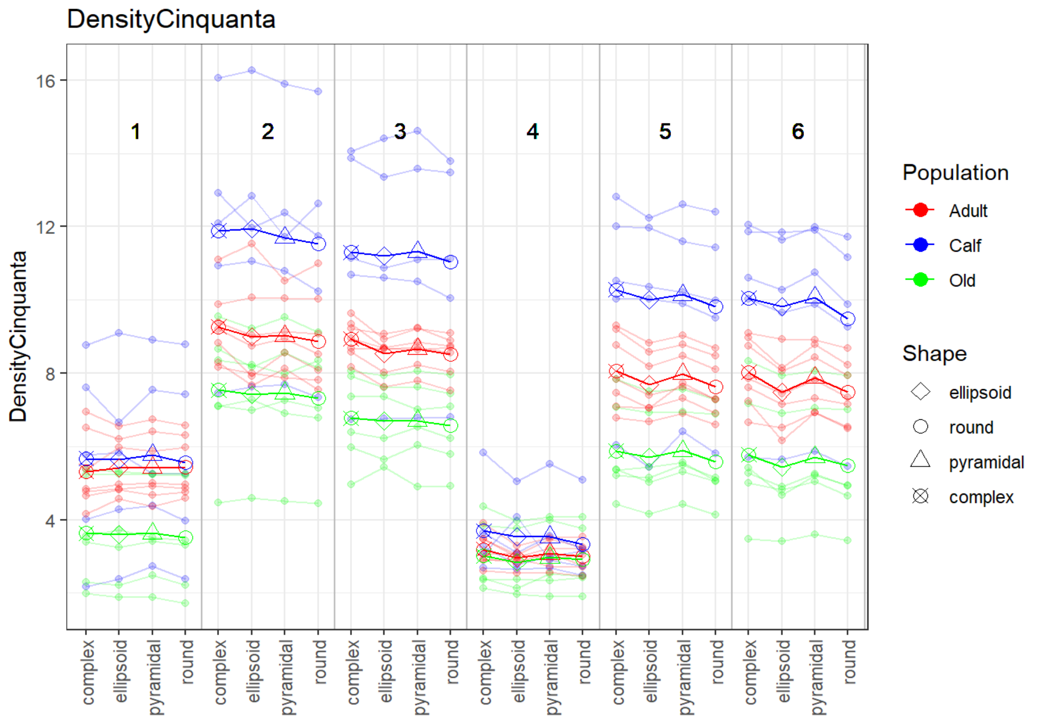

Density100

Population	Shape	Layer_ID	mean	sd
Adult	ellipsoid	1	15.06	2.97
Calf	ellipsoid	1	14.86	8.67
Old	ellipsoid	1	9.27	4.73
Adult	ellipsoid	2	21.87	5.47
Calf	ellipsoid	2	24.85	7.59
Old	ellipsoid	2	16.30	5.35
Adult	ellipsoid	3	25.07	1.83
Calf	ellipsoid	3	29.55	8.35
Old	ellipsoid	3	18.09	3.77
Adult	ellipsoid	4	4.26	0.61
Calf	ellipsoid	4	5.51	2.09
Old	ellipsoid	4	4.24	1.55
Adult	ellipsoid	5	21.68	3.22
Calf	ellipsoid	5	25.84	7.64
Old	ellipsoid	5	14.25	3.90
Adult	ellipsoid	6	21.55	3.45
Calf	ellipsoid	6	26.51	7.33
Old	ellipsoid	6	14.52	5.58
Adult	round	1	15.17	3.04
Calf	round	1	15.03	8.86
Old	round	1	9.06	4.71
Adult	round	2	21.59	4.98
Calf	round	2	24.36	7.76
Old	round	2	16.06	5.46
Adult	round	3	24.85	1.81

Population	Shape	Layer_ID	mean	sd
Calf	round	3	29.10	8.01
Old	round	3	17.69	3.87
Adult	round	4	4.29	0.73
Calf	round	4	5.19	2.24
Old	round	4	4.34	1.80
Adult	round	5	21.63	3.30
Calf	round	5	25.09	7.12
Old	round	5	14.13	3.85
Adult	round	6	21.69	3.05
Calf	round	6	26.09	7.13
Old	round	6	14.71	5.68
Adult	pyramidal	1	14.99	3.18
Calf	pyramidal	1	14.94	8.59
Old	pyramidal	1	9.23	4.59
Adult	pyramidal	2	21.23	4.05
Calf	pyramidal	2	24.40	7.36
Old	pyramidal	2	16.28	5.59
Adult	pyramidal	3	24.92	1.88
Calf	pyramidal	3	29.76	8.49
Old	pyramidal	3	17.76	4.01
Adult	pyramidal	4	4.48	0.69
Calf	pyramidal	4	5.55	2.40
Old	pyramidal	4	4.48	1.83
Adult	pyramidal	5	22.12	3.26
Calf	pyramidal	5	25.40	6.94
Old	pyramidal	5	14.59	3.73
Adult	pyramidal	6	22.27	3.09
Calf	pyramidal	6	26.82	7.48
Old	pyramidal	6	14.96	5.44
Adult	complex	1	14.60	3.29
Calf	complex	1	14.81	9.12
Old	complex	1	9.08	4.63
Adult	complex	2	21.68	4.49
Calf	complex	2	24.25	7.70
Old	complex	2	16.27	5.46
Adult	complex	3	25.40	1.69
Calf	complex	3	29.61	8.64
Old	complex	3	17.84	4.13
Adult	complex	4	4.32	0.97
Calf	complex	4	5.60	2.66
Old	complex	4	4.61	1.71
Adult	complex	5	22.27	3.87
Calf	complex	5	25.96	7.27
Old	complex	5	14.62	3.86
Adult	complex	6	22.47	3.34
Calf	complex	6	26.55	7.46

Population	Shape	Layer_ID	mean	sd
Old	complex	6	15.12	5.72

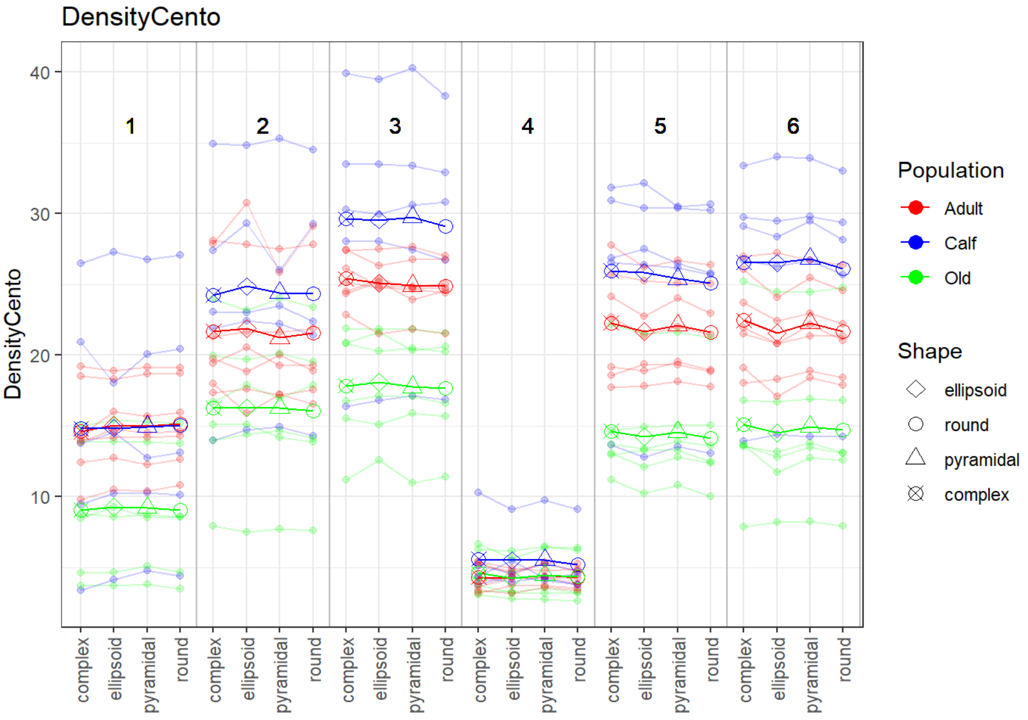

Radar Plot

Density

Principal Components

Layer 1

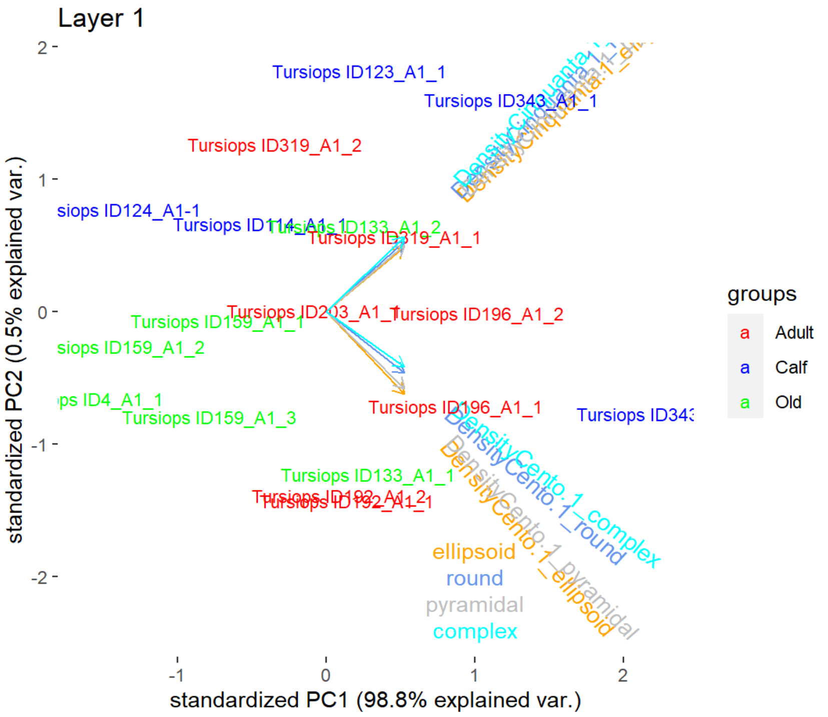

Layer 2

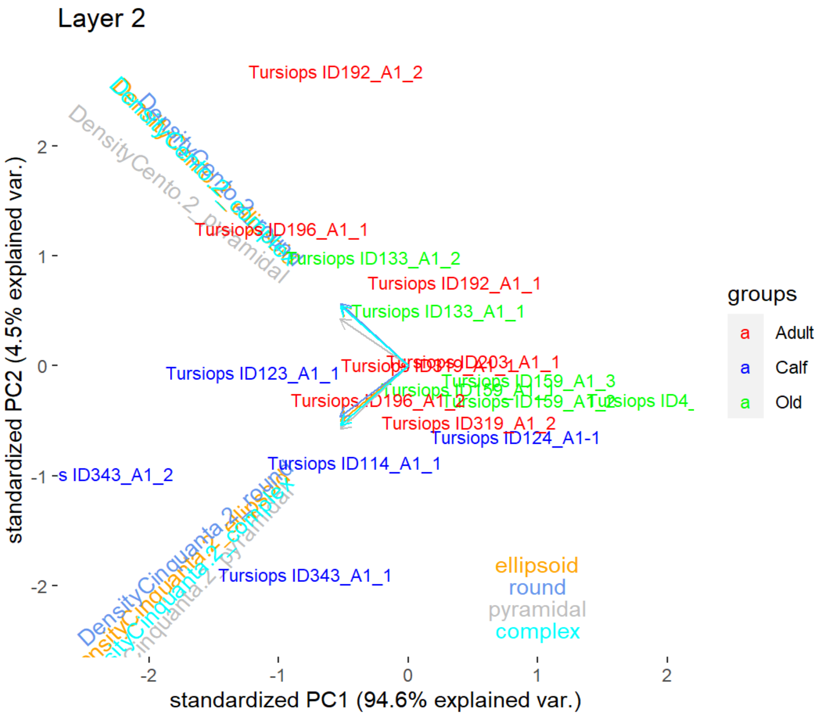

Layer 3

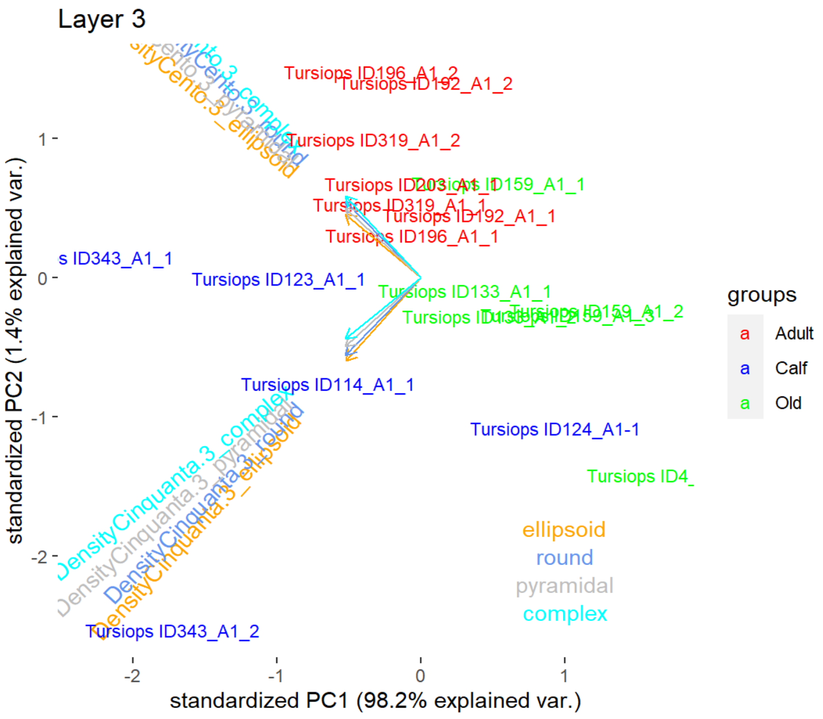

Layer 4

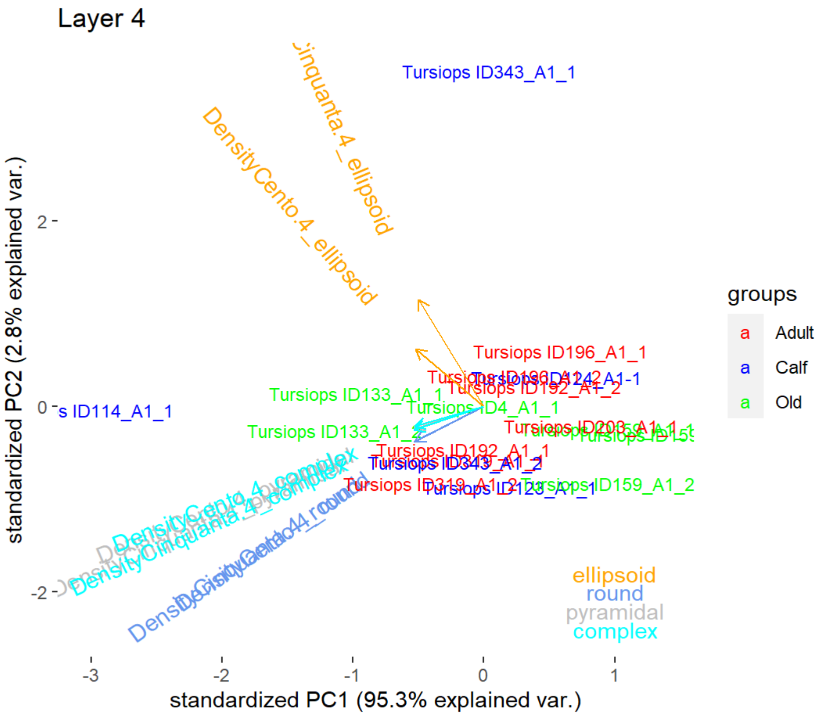

Layer 5

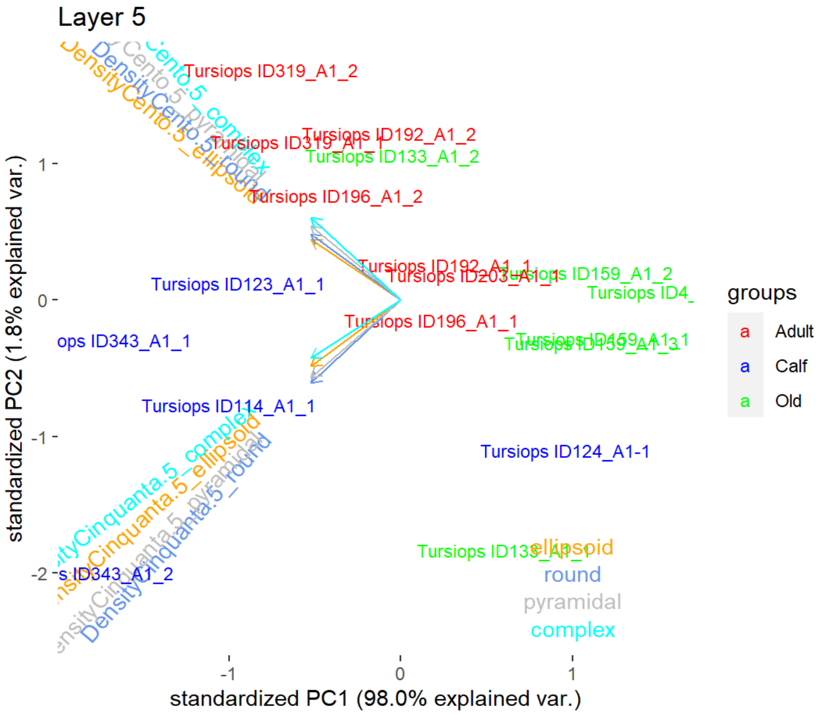

Layer 6

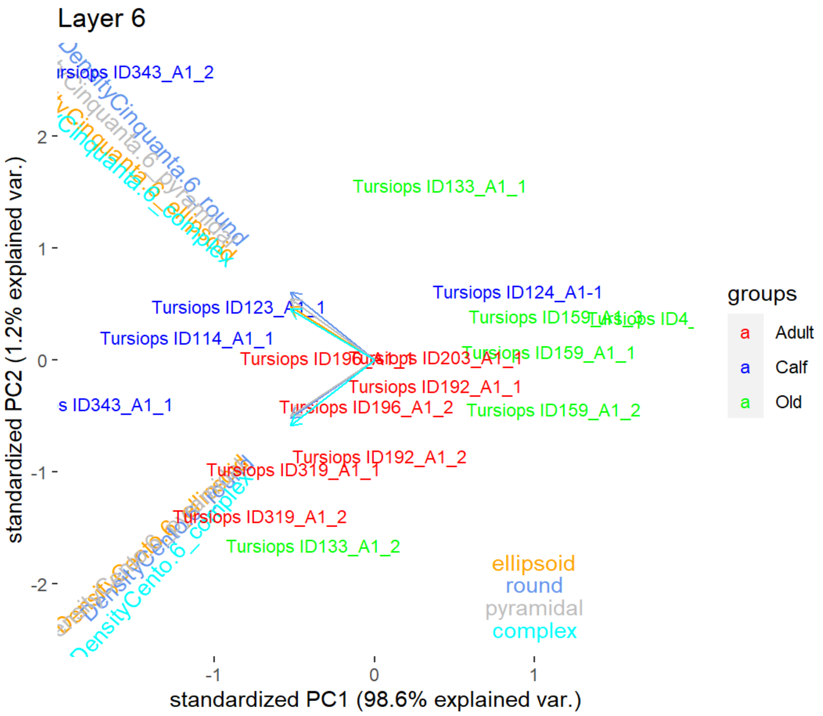

Inferential Analysis

Univariate

```
FALSE  Call:
FALSE  flip(Y = . ~ Population, data = DW, perms = nperms)
FALSE 4999 permutations.
FALSE
FALSE                                              Test    Stat tail
FALSE DensityCinquanta.1_ellipsoid_|_Population.Calf.    t  1.1082   ><
FALSE DensityCinquanta.1_ellipsoid_|_Population.Old.     t -2.3396   ><
FALSE DensityCento.1_ellipsoid_|_Population.Calf.        t  0.7608   ><
FALSE DensityCento.1_ellipsoid_|_Population.Old.         t -2.0513   ><
FALSE DensityCinquanta.2_ellipsoid_|_Population.Calf.    t  3.2189   ><
FALSE DensityCinquanta.2_ellipsoid_|_Population.Old.     t -2.3244   ><
FALSE DensityCento.2_ellipsoid_|_Population.Calf.        t  1.6021   ><
FALSE DensityCento.2_ellipsoid_|_Population.Old.         t -2.1976   ><
FALSE DensityCinquanta.3_ellipsoid_|_Population.Calf.    t  3.5553   ><
FALSE DensityCinquanta.3_ellipsoid_|_Population.Old.     t -2.8685   ><
FALSE DensityCento.3_ellipsoid_|_Population.Calf.        t  2.4718   ><
FALSE DensityCento.3_ellipsoid_|_Population.Old.         t -3.3188   ><
FALSE DensityCinquanta.4_ellipsoid_|_Population.Calf.    t  1.5723   ><
FALSE DensityCinquanta.4_ellipsoid_|_Population.Old.     t -0.8109   ><
FALSE DensityCento.4_ellipsoid_|_Population.Calf.        t  1.6777   ><
FALSE DensityCento.4_ellipsoid_|_Population.Old.         t -0.6291   ><
FALSE DensityCinquanta.5_ellipsoid_|_Population.Calf.    t  3.2201   ><
FALSE DensityCinquanta.5_ellipsoid_|_Population.Old.     t -3.0116   ><
FALSE DensityCento.5_ellipsoid_|_Population.Calf.        t  2.3856   ><
FALSE DensityCento.5_ellipsoid_|_Population.Old.         t -3.4631   ><
FALSE DensityCinquanta.6_ellipsoid_|_Population.Calf.    t  3.1670   ><
FALSE DensityCinquanta.6_ellipsoid_|_Population.Old.     t -3.0187   ><
FALSE DensityCento.6_ellipsoid_|_Population.Calf.        t  2.4677   ><
FALSE DensityCento.6_ellipsoid_|_Population.Old.         t -3.1152   ><
FALSE DensityCinquanta.1_round_|_Population.Calf.        t  1.0179   ><
FALSE DensityCinquanta.1_round_|_Population.Old.         t -2.3609   ><
FALSE DensityCento.1_round_|_Population.Calf.            t  0.8073   ><
FALSE DensityCento.1_round_|_Population.Old.             t -2.1443   ><
FALSE DensityCinquanta.2_round_|_Population.Calf.        t  3.0085   ><
FALSE DensityCinquanta.2_round_|_Population.Old.         t -2.2876   ><
FALSE DensityCento.2_round_|_Population.Calf.            t  1.5556   ><
FALSE DensityCento.2_round_|_Population.Old.             t -2.1895   ><
FALSE DensityCinquanta.3_round_|_Population.Calf.        t  3.4842   ><
FALSE DensityCinquanta.3_round_|_Population.Old.         t -3.0229   ><
FALSE DensityCento.3_round_|_Population.Calf.            t  2.4502   ><
FALSE DensityCento.3_round_|_Population.Old.             t -3.4514   ><
FALSE DensityCinquanta.4_round_|_Population.Calf.        t  0.9062   ><
FALSE DensityCinquanta.4_round_|_Population.Old.         t -0.4880   ><
FALSE DensityCento.4_round_|_Population.Calf.            t  1.0554   ><
FALSE DensityCento.4_round_|_Population.Old.             t -0.3547   ><
FALSE DensityCinquanta.5_round_|_Population.Calf.        t  3.2775   ><
FALSE DensityCinquanta.5_round_|_Population.Old.         t -3.2019   ><
FALSE DensityCento.5_round_|_Population.Calf.            t  2.2311   ><
FALSE DensityCento.5_round_|_Population.Old.             t -3.5717   ><
FALSE DensityCinquanta.6_round_|_Population.Calf.        t  2.9194   ><
FALSE DensityCinquanta.6_round_|_Population.Old.         t -2.9976   ><
FALSE DensityCento.6_round_|_Population.Calf.            t  2.3355   ><
FALSE DensityCento.6_round_|_Population.Old.             t -3.1323   ><
FALSE DensityCinquanta.1_pyramidal_|_Population.Calf.    t  1.2312   ><
FALSE DensityCinquanta.1_pyramidal_|_Population.Old.     t -2.3964   ><
FALSE DensityCento.1_pyramidal_|_Population.Calf.        t  0.8081   ><
FALSE DensityCento.1_pyramidal_|_Population.Old.         t -2.0745   ><
FALSE DensityCinquanta.2_pyramidal_|_Population.Calf.    t  3.1425   ><
FALSE DensityCinquanta.2_pyramidal_|_Population.Old.     t -2.3822   ><
FALSE DensityCento.2_pyramidal_|_Population.Calf.        t  1.7149   ><
FALSE DensityCento.2_pyramidal_|_Population.Old.         t -2.1752   ><
FALSE DensityCinquanta.3_pyramidal_|_Population.Calf.    t  3.4535   ><
FALSE DensityCinquanta.3_pyramidal_|_Population.Old.     t -2.9420   ><
FALSE DensityCento.3_pyramidal_|_Population.Calf.        t  2.5477   ><
FALSE DensityCento.3_pyramidal_|_Population.Old.         t -3.3242   ><
FALSE DensityCinquanta.4_pyramidal_|_Population.Calf.    t  1.1301   ><
FALSE DensityCinquanta.4_pyramidal_|_Population.Old.     t -0.6252   ><
FALSE DensityCento.4_pyramidal_|_Population.Calf.        t  1.2270   ><
FALSE DensityCento.4_pyramidal_|_Population.Old.         t -0.4658   ><
FALSE DensityCinquanta.5_pyramidal_|_Population.Calf.    t  3.3682   ><
FALSE DensityCinquanta.5_pyramidal_|_Population.Old.     t -3.4247   ><
FALSE DensityCento.5_pyramidal_|_Population.Calf.        t  2.2083   ><
FALSE DensityCento.5_pyramidal_|_Population.Old.         t -3.6510   ><
FALSE DensityCinquanta.6_pyramidal_|_Population.Calf.    t  3.1195   ><
FALSE DensityCinquanta.6_pyramidal_|_Population.Old.     t -3.1923   ><
FALSE DensityCento.6_pyramidal_|_Population.Calf.        t  2.3816   ><
FALSE DensityCento.6_pyramidal_|_Population.Old.         t -3.2305   ><
FALSE DensityCinquanta.1_complex_|_Population.Calf.      t  1.1269   ><
```

```
FALSE DensityCinquanta.1_complex_|_Population.Old.      t -2.1128   ><
FALSE DensityCento.1_complex_|_Population.Calf.         t  0.8291   ><
FALSE DensityCento.1_complex_|_Population.Old.          t -1.9351   ><
FALSE DensityCinquanta.2_complex_|_Population.Calf.     t  3.0079   ><
FALSE DensityCinquanta.2_complex_|_Population.Old.      t -2.4343   ><
FALSE DensityCento.2_complex_|_Population.Calf.         t  1.5247   ><
FALSE DensityCento.2_complex_|_Population.Old.          t -2.1942   ><
FALSE DensityCinquanta.3_complex_|_Population.Calf.     t  3.2180   ><
FALSE DensityCinquanta.3_complex_|_Population.Old.      t -3.1349   ><
FALSE DensityCento.3_complex_|_Population.Calf.         t  2.3455   ><
FALSE DensityCento.3_complex_|_Population.Old.          t -3.3978   ><
FALSE DensityCinquanta.4_complex_|_Population.Calf.     t  1.2244   ><
FALSE DensityCinquanta.4_complex_|_Population.Old.      t -0.7501   ><
FALSE DensityCento.4_complex_|_Population.Calf.         t  1.2279   ><
FALSE DensityCento.4_complex_|_Population.Old.          t -0.2327   ><
FALSE DensityCinquanta.5_complex_|_Population.Calf.     t  3.1328   ><
FALSE DensityCinquanta.5_complex_|_Population.Old.      t -3.2131   ><
FALSE DensityCento.5_complex_|_Population.Calf.         t  2.2418   ><
FALSE DensityCento.5_complex_|_Population.Old.          t -3.4971   ><
FALSE DensityCinquanta.6_complex_|_Population.Calf.     t  2.8495   ><
FALSE DensityCinquanta.6_complex_|_Population.Old.      t -3.1370   ><
FALSE DensityCento.6_complex_|_Population.Calf.         t  2.1996   ><
FALSE DensityCento.6_complex_|_Population.Old.          t -3.1359   ><
FALSE                                                p-value sig.
FALSE DensityCinquanta.1_ellipsoid_|_Population.Calf.  0.2674
FALSE DensityCinquanta.1_ellipsoid_|_Population.Old.   0.0250    *
FALSE DensityCento.1_ellipsoid_|_Population.Calf.      0.4330
FALSE DensityCento.1_ellipsoid_|_Population.Old.       0.0454    *
FALSE DensityCinquanta.2_ellipsoid_|_Population.Calf.  0.0028   **
FALSE DensityCinquanta.2_ellipsoid_|_Population.Old.   0.0250    *
FALSE DensityCento.2_ellipsoid_|_Population.Calf.      0.1180
FALSE DensityCento.2_ellipsoid_|_Population.Old.       0.0378    *
FALSE DensityCinquanta.3_ellipsoid_|_Population.Calf.  0.0020   **
FALSE DensityCinquanta.3_ellipsoid_|_Population.Old.   0.0088   **
FALSE DensityCento.3_ellipsoid_|_Population.Calf.      0.0214    *
FALSE DensityCento.3_ellipsoid_|_Population.Old.       0.0028   **
FALSE DensityCinquanta.4_ellipsoid_|_Population.Calf.  0.1140
FALSE DensityCinquanta.4_ellipsoid_|_Population.Old.   0.4146
FALSE DensityCento.4_ellipsoid_|_Population.Calf.      0.0898
FALSE DensityCento.4_ellipsoid_|_Population.Old.       0.5590
FALSE DensityCinquanta.5_ellipsoid_|_Population.Calf.  0.0046   **
FALSE DensityCinquanta.5_ellipsoid_|_Population.Old.   0.0088   **
FALSE DensityCento.5_ellipsoid_|_Population.Calf.      0.0278    *
FALSE DensityCento.5_ellipsoid_|_Population.Old.       0.0044   **
FALSE DensityCinquanta.6_ellipsoid_|_Population.Calf.  0.0054   **
FALSE DensityCinquanta.6_ellipsoid_|_Population.Old.   0.0074   **
FALSE DensityCento.6_ellipsoid_|_Population.Calf.      0.0236    *
FALSE DensityCento.6_ellipsoid_|_Population.Old.       0.0064   **
FALSE DensityCinquanta.1_round_|_Population.Calf.      0.3032
FALSE DensityCinquanta.1_round_|_Population.Old.       0.0240    *
FALSE DensityCento.1_round_|_Population.Calf.          0.4060
FALSE DensityCento.1_round_|_Population.Old.           0.0360    *
FALSE DensityCinquanta.2_round_|_Population.Calf.      0.0038   **
FALSE DensityCinquanta.2_round_|_Population.Old.       0.0254    *
FALSE DensityCento.2_round_|_Population.Calf.          0.1284
FALSE DensityCento.2_round_|_Population.Old.           0.0386    *
FALSE DensityCinquanta.3_round_|_Population.Calf.      0.0016   **
FALSE DensityCinquanta.3_round_|_Population.Old.       0.0056   **
FALSE DensityCento.3_round_|_Population.Calf.          0.0226    *
FALSE DensityCento.3_round_|_Population.Old.           0.0024   **
FALSE DensityCinquanta.4_round_|_Population.Calf.      0.3700
FALSE DensityCinquanta.4_round_|_Population.Old.       0.6196
FALSE DensityCento.4_round_|_Population.Calf.          0.2948
FALSE DensityCento.4_round_|_Population.Old.           0.7426
FALSE DensityCinquanta.5_round_|_Population.Calf.      0.0040   **
FALSE DensityCinquanta.5_round_|_Population.Old.       0.0058   **
FALSE DensityCento.5_round_|_Population.Calf.          0.0386    *
FALSE DensityCento.5_round_|_Population.Old.           0.0036   **
FALSE DensityCinquanta.6_round_|_Population.Calf.      0.0106    *
FALSE DensityCinquanta.6_round_|_Population.Old.       0.0074   **
FALSE DensityCento.6_round_|_Population.Calf.          0.0328    *
FALSE DensityCento.6_round_|_Population.Old.           0.0068   **
FALSE DensityCinquanta.1_pyramidal_|_Population.Calf.  0.2122
FALSE DensityCinquanta.1_pyramidal_|_Population.Old.   0.0220    *
FALSE DensityCento.1_pyramidal_|_Population.Calf.      0.4074
FALSE DensityCento.1_pyramidal_|_Population.Old.       0.0418    *
FALSE DensityCinquanta.2_pyramidal_|_Population.Calf.  0.0032   **
FALSE DensityCinquanta.2_pyramidal_|_Population.Old.   0.0198    *
```

```
FALSE DensityCento.2_pyramidal_|_Population.Calf.      0.0968
FALSE DensityCento.2_pyramidal_|_Population.Old.       0.0372    *
FALSE DensityCinquanta.3_pyramidal_|_Population.Calf.  0.0030   **
FALSE DensityCinquanta.3_pyramidal_|_Population.Old.   0.0076   **
FALSE DensityCento.3_pyramidal_|_Population.Calf.      0.0182    *
FALSE DensityCento.3_pyramidal_|_Population.Old.       0.0028   **
FALSE DensityCinquanta.4_pyramidal_|_Population.Calf.  0.2610
FALSE DensityCinquanta.4_pyramidal_|_Population.Old.   0.5430
FALSE DensityCento.4_pyramidal_|_Population.Calf.      0.2218
FALSE DensityCento.4_pyramidal_|_Population.Old.       0.6748
FALSE DensityCinquanta.5_pyramidal_|_Population.Calf.  0.0036   **
FALSE DensityCinquanta.5_pyramidal_|_Population.Old.   0.0038   **
FALSE DensityCento.5_pyramidal_|_Population.Calf.      0.0398    *
FALSE DensityCento.5_pyramidal_|_Population.Old.       0.0032   **
FALSE DensityCinquanta.6_pyramidal_|_Population.Calf.  0.0074   **
FALSE DensityCinquanta.6_pyramidal_|_Population.Old.   0.0048   **
FALSE DensityCento.6_pyramidal_|_Population.Calf.      0.0292    *
FALSE DensityCento.6_pyramidal_|_Population.Old.       0.0048   **
FALSE DensityCinquanta.1_complex_|_Population.Calf.    0.2540
FALSE DensityCinquanta.1_complex_|_Population.Old.     0.0392    *
FALSE DensityCento.1_complex_|_Population.Calf.        0.3852
FALSE DensityCento.1_complex_|_Population.Old.         0.0608
FALSE DensityCinquanta.2_complex_|_Population.Calf.    0.0034   **
FALSE DensityCinquanta.2_complex_|_Population.Old.     0.0176    *
FALSE DensityCento.2_complex_|_Population.Calf.        0.1342
FALSE DensityCento.2_complex_|_Population.Old.         0.0350    *
FALSE DensityCinquanta.3_complex_|_Population.Calf.    0.0044   **
FALSE DensityCinquanta.3_complex_|_Population.Old.     0.0054   **
FALSE DensityCento.3_complex_|_Population.Calf.        0.0280    *
FALSE DensityCento.3_complex_|_Population.Old.         0.0022   **
FALSE DensityCinquanta.4_complex_|_Population.Calf.    0.2260
FALSE DensityCinquanta.4_complex_|_Population.Old.     0.4638
FALSE DensityCento.4_complex_|_Population.Calf.        0.2204
FALSE DensityCento.4_complex_|_Population.Old.         0.8446
FALSE DensityCinquanta.5_complex_|_Population.Calf.    0.0060   **
FALSE DensityCinquanta.5_complex_|_Population.Old.     0.0058   **
FALSE DensityCento.5_complex_|_Population.Calf.        0.0374    *
FALSE DensityCento.5_complex_|_Population.Old.         0.0044   **
FALSE DensityCinquanta.6_complex_|_Population.Calf.    0.0120    *
FALSE DensityCinquanta.6_complex_|_Population.Old.     0.0054   **
FALSE DensityCento.6_complex_|_Population.Calf.        0.0396    *
FALSE DensityCento.6_complex_|_Population.Old.         0.0058   **
```

Cobined by aspects

```
FALSE  Call:
FALSE  npc(permTP = res, subsets = ss_asp)
FALSE  permutations.
FALSE
FALSE                 comb.funct nVar  Stat p-value Adjust:maxT sig.
FALSE DensityCinquanta     Fisher   48 192.5  0.0008      0.0008  ***
FALSE DensityCento         Fisher   48 154.9  0.0034      0.0034   **
```

Cobined by Shape and Layer

```
FALSE  Call:
FALSE  npc(permTP = res, subsets = ids)
FALSE  permutations.
FALSE
FALSE             comb.funct nVar   Stat p-value Adjust:maxT sig.
FALSE ellipsoid_1     Fisher    4  8.937  0.0676      0.1786
FALSE ellipsoid_2     Fisher    4 14.980  0.0062      0.0284    *
FALSE ellipsoid_3     Fisher    4 20.670  0.0002      0.0044   **
FALSE ellipsoid_4     Fisher    4  6.044  0.1858      0.2750
FALSE ellipsoid_5     Fisher    4 19.124  0.0012      0.0082   **
FALSE ellipsoid_6     Fisher    4 18.926  0.0016      0.0086   **
FALSE round_1         Fisher    4  9.149  0.0646      0.1692
FALSE round_2         Fisher    4 14.553  0.0070      0.0318    *
FALSE round_3         Fisher    4 21.445  0.0002      0.0020   **
FALSE round_4         Fisher    4  2.992  0.5512      0.5512
FALSE round_5         Fisher    4 19.553  0.0010      0.0066   **
FALSE round_6         Fisher    4 17.861  0.0020      0.0110    *
FALSE pyramidal_1     Fisher    4  9.440  0.0568      0.1542
FALSE pyramidal_2     Fisher    4 15.293  0.0044      0.0254    *
FALSE pyramidal_3     Fisher    4 20.573  0.0006      0.0046   **
FALSE pyramidal_4     Fisher    4  3.853  0.4172      0.4646
FALSE pyramidal_5     Fisher    4 20.168  0.0008      0.0048   **
FALSE pyramidal_6     Fisher    4 19.118  0.0016      0.0082   **
FALSE complex_1       Fisher    4  8.364  0.0844      0.2036
FALSE complex_2       Fisher    4 15.085  0.0048      0.0274    *
FALSE complex_3       Fisher    4 20.342  0.0004      0.0048   **
FALSE complex_4       Fisher    4  3.937  0.3988      0.4646
FALSE complex_5       Fisher    4 18.978  0.0012      0.0086   **
FALSE complex_6       Fisher    4 18.023  0.0018      0.0102    *
```

Cobined by Layer

```
FALSE  Call:
FALSE  npc(permTP = res, subsets = ss_Layers)
FALSE  permutations.
FALSE
FALSE   comb.funct nVar  Stat p-value Adjust:maxT sig.
FALSE 1     Fisher   16 35.89  0.0664      0.1212
FALSE 2     Fisher   16 59.91  0.0050      0.0176    *
FALSE 3     Fisher   16 83.03  0.0002      0.0014   **
FALSE 4     Fisher   16 16.83  0.3588      0.3588
FALSE 5     Fisher   16 77.82  0.0010      0.0034   **
FALSE 6     Fisher   16 73.93  0.0020      0.0058   **
```

Pairwise comparisons

```
FALSE
FALSE         _-_-_-_-_-_-_-_-_-_- VARIABLE  DensityCinquanta.1_ellipsoid _-_-_-_-_-_-_-_-_-_-
FALSE
FALSE         _-_-_-_-_-_-_-_-_-_- Layer  1 _-_-_-_-_-_-_-_-_-_-
FALSE                          Raw (upper)
FALSE Adjusted (lower) Adult Calf    Old
FALSE            Adult    NA 0.83 -0.021
FALSE            Calf  0.830   NA -0.130
FALSE            Old   0.064 0.13     NA
FALSE
FALSE         _-_-_-_-_-_-_-_-_-_- Layer  2 _-_-_-_-_-_-_-_-_-_-
FALSE                          Raw (upper)
FALSE Adjusted (lower) Adult  Calf    Old
FALSE            Adult    NA 0.047 -0.077
FALSE            Calf  0.047    NA -0.011
FALSE            Old   0.077 0.033     NA
FALSE
FALSE         _-_-_-_-_-_-_-_-_-_- Layer  3 _-_-_-_-_-_-_-_-_-_-
FALSE                          Raw (upper)
FALSE Adjusted (lower) Adult  Calf    Old
FALSE            Adult    NA 0.037 -0.002
FALSE            Calf  0.037    NA -0.010
FALSE            Old   0.005 0.010     NA
FALSE
FALSE         _-_-_-_-_-_-_-_-_-_- Layer  4 _-_-_-_-_-_-_-_-_-_-
FALSE                          Raw (upper)
FALSE Adjusted (lower) Adult  Calf    Old
FALSE            Adult    NA 0.173 -0.813
FALSE            Calf  0.518    NA -0.277
FALSE            Old   0.813 0.518     NA
FALSE
FALSE         _-_-_-_-_-_-_-_-_-_- Layer  5 _-_-_-_-_-_-_-_-_-_-
FALSE                          Raw (upper)
FALSE Adjusted (lower) Adult  Calf   Old
FALSE            Adult    NA 0.066 -0.01
FALSE            Calf  0.066    NA -0.01
FALSE            Old   0.029 0.029    NA
FALSE
FALSE         _-_-_-_-_-_-_-_-_-_- Layer  6 _-_-_-_-_-_-_-_-_-_-
FALSE                          Raw (upper)
FALSE Adjusted (lower) Adult  Calf    Old
FALSE            Adult    NA 0.052 -0.022
FALSE            Calf  0.052    NA -0.012
FALSE            Old   0.035 0.035     NA
FALSE
FALSE         _-_-_-_-_-_-_-_-_-_- VARIABLE  DensityCento.1_ellipsoid _-_-_-_-_-_-_-_-_-_-
FALSE
FALSE         _-_-_-_-_-_-_-_-_-_- Layer  1 _-_-_-_-_-_-_-_-_-_-
FALSE                          Raw (upper)
FALSE Adjusted (lower) Adult   Calf    Old
FALSE            Adult    NA -0.947 -0.022
FALSE            Calf  0.947     NA -0.204
FALSE            Old   0.066  0.204     NA
FALSE
FALSE         _-_-_-_-_-_-_-_-_-_- Layer  2 _-_-_-_-_-_-_-_-_-_-
FALSE                          Raw (upper)
FALSE Adjusted (lower) Adult  Calf    Old
FALSE            Adult    NA 0.454 -0.100
FALSE            Calf  0.454    NA -0.056
FALSE            Old   0.167 0.167     NA
FALSE
FALSE         _-_-_-_-_-_-_-_-_-_- Layer  3 _-_-_-_-_-_-_-_-_-_-
FALSE                          Raw (upper)
FALSE Adjusted (lower) Adult  Calf    Old
FALSE            Adult    NA 0.196 -0.002
FALSE            Calf  0.196    NA -0.015
FALSE            Old   0.005 0.015     NA
FALSE
FALSE         _-_-_-_-_-_-_-_-_-_- Layer  4 _-_-_-_-_-_-_-_-_-_-
FALSE                          Raw (upper)
FALSE Adjusted (lower) Adult  Calf    Old
FALSE            Adult    NA 0.109  0.932
FALSE            Calf  0.328    NA -0.306
FALSE            Old   0.932 0.328     NA
FALSE
FALSE         _-_-_-_-_-_-_-_-_-_- Layer  5 _-_-_-_-_-_-_-_-_-_-
FALSE                          Raw (upper)
FALSE Adjusted (lower) Adult  Calf    Old
```

```
FALSE            Adult    NA 0.209 -0.007
FALSE            Calf  0.209    NA -0.019
FALSE            Old   0.021 0.021     NA
FALSE
FALSE         _-_-_-_-_-_-_-_- Layer  6 _-_-_-_-_-_-_-_-
FALSE                        Raw (upper)
FALSE Adjusted (lower) Adult  Calf    Old
FALSE            Adult    NA 0.149 -0.015
FALSE            Calf  0.149    NA -0.017
FALSE            Old   0.045 0.045     NA
FALSE
FALSE         _-_-_-_-_-_-_-_- VARIABLE  DensityCinquanta.1_round _-_-_-_-_-_-_-_-
FALSE
FALSE         _-_-_-_-_-_-_-_- Layer  1 _-_-_-_-_-_-_-_-
FALSE                        Raw (upper)
FALSE Adjusted (lower) Adult  Calf    Old
FALSE            Adult    NA 0.887 -0.019
FALSE            Calf  0.887    NA -0.137
FALSE            Old   0.056 0.137     NA
FALSE
FALSE         _-_-_-_-_-_-_-_- Layer  2 _-_-_-_-_-_-_-_-
FALSE                        Raw (upper)
FALSE Adjusted (lower) Adult  Calf    Old
FALSE            Adult    NA 0.062 -0.077
FALSE            Calf  0.062    NA -0.012
FALSE            Old   0.077 0.037     NA
FALSE
FALSE         _-_-_-_-_-_-_-_- Layer  3 _-_-_-_-_-_-_-_-
FALSE                        Raw (upper)
FALSE Adjusted (lower) Adult  Calf    Old
FALSE            Adult    NA 0.039 -0.001
FALSE            Calf  0.039    NA -0.008
FALSE            Old   0.002 0.008     NA
FALSE
FALSE         _-_-_-_-_-_-_-_- Layer  4 _-_-_-_-_-_-_-_-
FALSE                        Raw (upper)
FALSE Adjusted (lower) Adult  Calf    Old
FALSE            Adult    NA 0.525 -0.884
FALSE            Calf      1    NA -0.504
FALSE            Old       1 1.000     NA
FALSE
FALSE         _-_-_-_-_-_-_-_- Layer  5 _-_-_-_-_-_-_-_-
FALSE                        Raw (upper)
FALSE Adjusted (lower) Adult  Calf    Old
FALSE            Adult    NA 0.052 -0.009
FALSE            Calf  0.052    NA -0.010
FALSE            Old   0.026 0.026     NA
FALSE
FALSE         _-_-_-_-_-_-_-_- Layer  6 _-_-_-_-_-_-_-_-
FALSE                        Raw (upper)
FALSE Adjusted (lower) Adult  Calf    Old
FALSE            Adult    NA 0.073 -0.016
FALSE            Calf  0.073    NA -0.021
FALSE            Old   0.049 0.049     NA
FALSE
FALSE         _-_-_-_-_-_-_-_- VARIABLE  DensityCento.1_round _-_-_-_-_-_-_-_-
FALSE
FALSE         _-_-_-_-_-_-_-_- Layer  1 _-_-_-_-_-_-_-_-
FALSE                        Raw (upper)
FALSE Adjusted (lower) Adult   Calf    Old
FALSE            Adult    NA -0.972 -0.017
FALSE            Calf  0.972     NA -0.190
FALSE            Old   0.050  0.190     NA
FALSE
FALSE         _-_-_-_-_-_-_-_- Layer  2 _-_-_-_-_-_-_-_-
FALSE                        Raw (upper)
FALSE Adjusted (lower) Adult  Calf    Old
FALSE            Adult    NA 0.465 -0.094
FALSE            Calf  0.465    NA -0.069
FALSE            Old   0.206 0.206     NA
FALSE
FALSE         _-_-_-_-_-_-_-_- Layer  3 _-_-_-_-_-_-_-_-
FALSE                        Raw (upper)
FALSE Adjusted (lower) Adult  Calf    Old
FALSE            Adult    NA 0.208 -0.002
FALSE            Calf  0.208    NA -0.013
FALSE            Old   0.005 0.013     NA
FALSE
```

```
FALSE         _-_-_-_-_-_-_-_-_-_- Layer  4 _-_-_-_-_-_-_-_-_-_-
FALSE                          Raw (upper)
FALSE Adjusted (lower) Adult Calf    Old
FALSE             Adult    NA  0.4  0.887
FALSE             Calf      1   NA -0.550
FALSE             Old       1  1.0     NA
FALSE
FALSE         _-_-_-_-_-_-_-_-_-_- Layer  5 _-_-_-_-_-_-_-_-_-_-
FALSE                          Raw (upper)
FALSE Adjusted (lower) Adult  Calf    Old
FALSE             Adult    NA 0.272 -0.006
FALSE             Calf  0.272    NA -0.019
FALSE             Old   0.017 0.019     NA
FALSE
FALSE         _-_-_-_-_-_-_-_-_-_- Layer  6 _-_-_-_-_-_-_-_-_-_-
FALSE                          Raw (upper)
FALSE Adjusted (lower) Adult  Calf    Old
FALSE             Adult    NA 0.177 -0.018
FALSE             Calf  0.177    NA -0.022
FALSE             Old   0.053 0.053     NA
FALSE
FALSE         _-_-_-_-_-_-_-_-_-_- VARIABLE  DensityCinquanta.1_pyramidal _-_-_-_-_-_-_-_-_-_-
FALSE
FALSE         _-_-_-_-_-_-_-_-_-_- Layer  1 _-_-_-_-_-_-_-_-_-_-
FALSE                          Raw (upper)
FALSE Adjusted (lower) Adult  Calf    Old
FALSE             Adult    NA 0.735 -0.021
FALSE             Calf  0.735    NA -0.100
FALSE             Old   0.062 0.100     NA
FALSE
FALSE         _-_-_-_-_-_-_-_-_-_- Layer  2 _-_-_-_-_-_-_-_-_-_-
FALSE                          Raw (upper)
FALSE Adjusted (lower) Adult  Calf    Old
FALSE             Adult    NA 0.037 -0.056
FALSE             Calf  0.037    NA -0.011
FALSE             Old   0.056 0.033     NA
FALSE
FALSE         _-_-_-_-_-_-_-_-_-_- Layer  3 _-_-_-_-_-_-_-_-_-_-
FALSE                          Raw (upper)
FALSE Adjusted (lower) Adult  Calf    Old
FALSE             Adult    NA 0.038 -0.001
FALSE             Calf  0.038    NA -0.011
FALSE             Old   0.004 0.011     NA
FALSE
FALSE         _-_-_-_-_-_-_-_-_-_- Layer  4 _-_-_-_-_-_-_-_-_-_-
FALSE                          Raw (upper)
FALSE Adjusted (lower) Adult  Calf    Old
FALSE             Adult    NA 0.429 -0.836
FALSE             Calf      1    NA -0.434
FALSE             Old       1 1.000     NA
FALSE
FALSE         _-_-_-_-_-_-_-_-_-_- Layer  5 _-_-_-_-_-_-_-_-_-_-
FALSE                          Raw (upper)
FALSE Adjusted (lower) Adult  Calf    Old
FALSE             Adult    NA 0.043 -0.005
FALSE             Calf  0.043    NA -0.008
FALSE             Old   0.015 0.015     NA
FALSE
FALSE         _-_-_-_-_-_-_-_-_-_- Layer  6 _-_-_-_-_-_-_-_-_-_-
FALSE                          Raw (upper)
FALSE Adjusted (lower) Adult  Calf    Old
FALSE             Adult    NA 0.062 -0.013
FALSE             Calf  0.062    NA -0.008
FALSE             Old   0.025 0.025     NA
FALSE
FALSE         _-_-_-_-_-_-_-_-_-_- VARIABLE  DensityCento.1_pyramidal _-_-_-_-_-_-_-_-_-_-
FALSE
FALSE         _-_-_-_-_-_-_-_-_-_- Layer  1 _-_-_-_-_-_-_-_-_-_-
FALSE                          Raw (upper)
FALSE Adjusted (lower) Adult   Calf    Old
FALSE             Adult    NA -0.987 -0.022
FALSE             Calf  0.987     NA -0.197
FALSE             Old   0.067  0.197     NA
FALSE
FALSE         _-_-_-_-_-_-_-_-_-_- Layer  2 _-_-_-_-_-_-_-_-_-_-
FALSE                          Raw (upper)
FALSE Adjusted (lower) Adult  Calf    Old
FALSE             Adult    NA 0.378 -0.091
```

```
FALSE            Calf  0.378    NA -0.067
FALSE            Old   0.201 0.201     NA
FALSE
FALSE         _-_-_-_-_-_-_-_- Layer  3 _-_-_-_-_-_-_-_-
FALSE                          Raw (upper)
FALSE Adjusted (lower) Adult  Calf    Old
FALSE            Adult    NA 0.174 -0.001
FALSE            Calf  0.174    NA -0.013
FALSE            Old   0.004 0.013     NA
FALSE
FALSE         _-_-_-_-_-_-_-_- Layer  4 _-_-_-_-_-_-_-_-
FALSE                          Raw (upper)
FALSE Adjusted (lower) Adult  Calf    Old
FALSE            Adult    NA 0.318  0.932
FALSE            Calf  0.953    NA -0.453
FALSE            Old   0.953 0.953     NA
FALSE
FALSE         _-_-_-_-_-_-_-_- Layer  5 _-_-_-_-_-_-_-_-
FALSE                          Raw (upper)
FALSE Adjusted (lower) Adult  Calf    Old
FALSE            Adult    NA 0.296 -0.006
FALSE            Calf  0.296    NA -0.021
FALSE            Old   0.019 0.021     NA
FALSE
FALSE         _-_-_-_-_-_-_-_- Layer  6 _-_-_-_-_-_-_-_-
FALSE                          Raw (upper)
FALSE Adjusted (lower) Adult  Calf    Old
FALSE            Adult    NA 0.186 -0.013
FALSE            Calf  0.186    NA -0.022
FALSE            Old   0.040 0.040     NA
FALSE
FALSE         _-_-_-_-_-_-_-_- VARIABLE  DensityCinquanta.1_complex _-_-_-_-_-_-_-_-
FALSE
FALSE         _-_-_-_-_-_-_-_- Layer  1 _-_-_-_-_-_-_-_-
FALSE                          Raw (upper)
FALSE Adjusted (lower) Adult  Calf    Old
FALSE            Adult    NA 0.749 -0.027
FALSE            Calf  0.749    NA -0.137
FALSE            Old   0.080 0.137     NA
FALSE
FALSE         _-_-_-_-_-_-_-_- Layer  2 _-_-_-_-_-_-_-_-
FALSE                          Raw (upper)
FALSE Adjusted (lower) Adult  Calf    Old
FALSE            Adult    NA 0.056 -0.042
FALSE            Calf  0.056    NA -0.017
FALSE            Old   0.051 0.051     NA
FALSE
FALSE         _-_-_-_-_-_-_-_- Layer  3 _-_-_-_-_-_-_-_-
FALSE                          Raw (upper)
FALSE Adjusted (lower) Adult  Calf    Old
FALSE            Adult    NA 0.069 -0.001
FALSE            Calf  0.069    NA -0.009
FALSE            Old   0.004 0.009     NA
FALSE
FALSE         _-_-_-_-_-_-_-_- Layer  4 _-_-_-_-_-_-_-_-
FALSE                          Raw (upper)
FALSE Adjusted (lower) Adult Calf    Old
FALSE            Adult    NA 0.39 -0.752
FALSE            Calf      1   NA -0.378
FALSE            Old       1 1.00     NA
FALSE
FALSE         _-_-_-_-_-_-_-_- Layer  5 _-_-_-_-_-_-_-_-
FALSE                          Raw (upper)
FALSE Adjusted (lower) Adult  Calf    Old
FALSE            Adult    NA 0.075 -0.011
FALSE            Calf  0.075    NA -0.010
FALSE            Old   0.029 0.029     NA
FALSE
FALSE         _-_-_-_-_-_-_-_- Layer  6 _-_-_-_-_-_-_-_-
FALSE                          Raw (upper)
FALSE Adjusted (lower) Adult  Calf    Old
FALSE            Adult    NA 0.085 -0.013
FALSE            Calf  0.085    NA -0.016
FALSE            Old   0.040 0.040     NA
FALSE
FALSE         _-_-_-_-_-_-_-_- VARIABLE  DensityCento.1_complex _-_-_-_-_-_-_-_-
FALSE
FALSE         _-_-_-_-_-_-_-_- Layer  1 _-_-_-_-_-_-_-_-
```

```
FALSE                          Raw (upper)
FALSE Adjusted (lower) Adult  Calf    Old
FALSE            Adult    NA 0.961 -0.031
FALSE            Calf  0.961    NA -0.220
FALSE            Old   0.094 0.220     NA
FALSE
FALSE         _-_-_-_-_-_-_-_- Layer  2 _-_-_-_-_-_-_-_-
FALSE                          Raw (upper)
FALSE Adjusted (lower) Adult  Calf    Old
FALSE            Adult    NA 0.489 -0.083
FALSE            Calf  0.489    NA -0.079
FALSE            Old   0.237 0.237     NA
FALSE
FALSE         _-_-_-_-_-_-_-_- Layer  3 _-_-_-_-_-_-_-_-
FALSE                          Raw (upper)
FALSE Adjusted (lower) Adult  Calf    Old
FALSE            Adult    NA 0.250  0.000
FALSE            Calf  0.250    NA -0.015
FALSE            Old   0.001 0.015     NA
FALSE
FALSE         _-_-_-_-_-_-_-_- Layer  4 _-_-_-_-_-_-_-_-
FALSE                          Raw (upper)
FALSE Adjusted (lower) Adult  Calf    Old
FALSE            Adult    NA 0.291  0.646
FALSE            Calf  0.872    NA -0.545
FALSE            Old   0.872 0.872     NA
FALSE
FALSE         _-_-_-_-_-_-_-_- Layer  5 _-_-_-_-_-_-_-_-
FALSE                          Raw (upper)
FALSE Adjusted (lower) Adult  Calf    Old
FALSE            Adult    NA 0.268 -0.008
FALSE            Calf  0.268    NA -0.018
FALSE            Old   0.025 0.025     NA
FALSE
FALSE         _-_-_-_-_-_-_-_- Layer  6 _-_-_-_-_-_-_-_-
FALSE                          Raw (upper)
FALSE Adjusted (lower) Adult  Calf    Old
FALSE            Adult    NA 0.221 -0.016
FALSE            Calf  0.221    NA -0.021
FALSE            Old   0.048 0.048     NA
```
